# Genomic dissection of phenological and yield-associated traits in lentil (*Lens culinaris* Medik.) using genome-wide association mapping

**DOI:** 10.1007/s12298-026-01739-x

**Published:** 2026-04-07

**Authors:** Yogesh Dashrath Naik, Vinay Kumar Sharma, Muraleedhar S. Aski, K. M. Shivaprasad, Mangesh Pralhad Jadhav, Manda Sriswathi, Damaris A. Odeny, Kumari Anjani, Somashekhar Punnuri, Rajeev K. Varshney, Himabindu Kudapa, Mahendar Thudi

**Affiliations:** 1 Department of Agricultural Biotechnology and Molecular Biology, Dr. Rajendra Prasad Central Agricultural University (RPCAU), Pusa, Bihar India; 2 Division of Genetics, Indian Council of Agricultural Research (ICAR)-Indian Agricultural Research Institute (IARI), Pusa, New Delhi India; 3 Genetics and Tree Improvement division, Indian Council of Forestry Research and Education (ICFRE)-Institute of Forest Biodiversity, Hyderabad, Telangana India; 4 Department of Molecular Biology and Biotechnology, University of Agricultural Sciences, Dharwad, Karnataka India; 5 Global Research Program-Accelerated Crop Improvement, International Crops Research Institute for the Semi-Arid Tropics (ICRISAT), Patancheru, Hyderabad India; 6 College of Agriculture, Family Sciences and Technology, 1005 State University Dr, Fort Valley State University, Fort Valley, GA USA; 7 WA State Agricultural Biotechnology Centre, Centre for Crop and Food Innovation, Murdoch University, Murdoch, WA 6150 Australia; 8 Centre for Crop Health and School of Agriculture and Environmental Science, University of Southern Queensland, Toowoomba, Australia

**Keywords:** Flowering time, GWAS, Lentil, Marker–trait associations, *MYB* transcription factor, SNPs

## Abstract

Lentil (*Lens culinaris* Medik.), a nutrient-rich legume cultivated worldwide, plays a vital role in combating malnutrition and hidden hunger. Understanding the genetic architecture underlying key phenological and agronomic traits in lentil is crucial for accelerating molecular breeding. In this study, genome-wide association mapping was conducted using 142 genetically diverse lentil accessions, evaluated across two field environments over two years. High-throughput sequencing generated 34,995 high-quality single-nucleotide polymorphisms, which were used for genetic characterization and for the identification of marker-trait associations for phenological and yield-associated traits. Population structure analysis identified three subpopulations (K = 3), with UPGMA clustering showing a similar pattern. Association mapping was performed using multi-locus models and further confirmed through a single-locus generalized linear model. A total of 64 significant associations were identified, of which Chr5_342836807 and Chr6_200603138 were consistently detected across all environments for days to 50% flowering. Putative candidate genes located near these phenology-associated loci such as abscisate β-glucosyltransferase, pentatricopeptide repeat proteins, and transcription factors from the *MYB*, *MADS-box*, and *GRAS* families are likely involved in flowering-time regulation in lentil. These findings reveal novel associations between genetic variants and complex traits and identify putative genes that may be exploited in marker-assisted selection and genomic prediction strategies.

**Supplementary Information:**

The online version contains supplementary material available at 10.1007/s12298-026-01739-x.

## Introduction

Lentil (*Lens culinaris* Medik.) is a diploid (2*n* = 2*x* = 14; 4,063 Mbp) and economically important pulse crop, globally valued for its high protein content, micronutrients, vitamins and dietary fibre (Joehnke et al. 2021). It is one of the major cool-season grain legumes cultivated worldwide and occupies approximately 5.68 million hectares, with an average productivity of 1,245.4 kg ha^−1^ (FAO 2023). Its wide cultivation across diverse agro-climatic regions highlights its adaptability to varied climates and soil types, underscoring its importance as a resilient crop for sustainable agriculture and climate change adaptation (Sarker and Erskine 2006). Globally, the cultivated area expanded from 3.82 million hectares in the early 2000s to 5.68 million hectares in 2023, showcasing increased interest due to its agricultural and nutritional benefits.

Lentil is predominantly cultivated as a rainfed crop that relies on residual soil moisture, often without supplemental irrigation. Its growth cycle thrives in a narrow temperature range, requiring cooler temperatures during the vegetative stage and warmer conditions during maturation, typically between 18 and 30 °C (Choudhury et al. 2012). However, the crop faces significant challenges when exposed to elevated temperatures and moisture stress during its reproductive phase. Heat stress in lentil has been associated with significant oxidative damage, membrane degradation and reduced leaf sugar content, along with a marked decline in pollen fertility (Bhandari et al. 2020; Sarkar et al. 2025). Such physiological disruptions, particularly during the reproductive stage, can severely compromise seed quality and ultimately lead to substantial yield losses (Bourgault et al. 2018; Venugopalan et al. 2021; Bansal et al. 2023). In addition, drought stress negatively impacts lentil growth and development, further contributing to yield reductions (Noor et al. 2024). These challenges are particularly severe in dryland, semi-arid and rice-fallow regions. In rice-fallow systems, delayed sowing of lentil due to late rice harvesting exposes the crop to extreme climatic conditions, leading to yield reductions of up to 49 kg ha^−1^ for every week of delay beyond the optimal planting window (Tefera et al. 2024). In this context, breeding climate-resilient lentil varieties has become a global priority. To address these challenges, early flowering and maturity traits have emerged as critical solutions in mitigating yield losses under heat and moisture stress. These traits allow lentil to shorten its growing season, enabling them to escape adverse conditions during the reproductive stage while also facilitating the feasibility of double cropping in semi-arid regions and rice-fallow areas globally. This dual benefit not only mitigates the risks posed by climatic variability but also enhances agricultural sustainability by promoting legumes in mono-cropped systems (Erskine et al. 2011; Yadav et al. 2015; Yuan et al. 2021). Although early flowering may limit yield potential under optimal conditions, it generally results in lower yield penalties under under stress conditions compared to late-flowering genotypes (Naik et al. 2025). Studies have shown that early flowering lines often carry multiple early alleles, some of which exhibit additive effects on phenology, further highlighting the intricate nature of these traits (Lake et al. 2024).

To better understand complex traits, it is essential to dissect their genetic architecture and identify genomic regions controlling trait expression. Genome-wide association studies (GWAS) provide a powerful approach for uncovering the genetic basis of complex traits. However, a major challenge in GWAS is distinguishing true marker–trait associations (MTAs) from false positives caused by population structure and kinship (Gangurde et al. 2022). Although single-locus mixed linear models reduce spurious associations by accounting for these confounding factors, they may lead to overfitting and false-negative results (Liu et al. 2016). To overcome these limitations, multi-locus GWAS models such as Bayesian-information and Linkage-disequilibrium Iteratively Nested Keyway (BLINK) and the Fixed and Random Model Circulating Probability Unification (FarmCPU) have been developed (Liu et al. 2016; Huang et al. 2019). FarmCPU increases statistical power by iteratively applying fixed and random effect models while simultaneously considering multiple markers (Liu et al. 2016), whereas BLINK further improves efficiency and power by using fixed effects and incorporating linkage disequilibrium (LD) through a Bayesian information criterion-based approach (Huang et al. 2019).

In lentil, substantial progress has been made in mapping flowering time through QTL analyses (Yuan et al. 2021; Haile et al. 2021; Rajandran et al. 2022). However, only a limited number of studies have employed GWAS to dissect the genetic basis of flowering time and maturity traits, which are crucial for adapting lentil to rice-fallow systems and climate-stressed environments (Rajendran et al. 2021). Most previous studies relied on bi-parental populations or single-locus GWAS with limited marker density and population size, resulting in low mapping resolution and the detection of only a few major-effect loci. In the present study, high-density SNP markers combined with multi-locus GWAS models were used to identify robust and biologically meaningful associations. The identified SNPs associated with days to flowering, maturity and key agronomic traits provide valuable resources for marker-assisted selection and molecular breeding aimed at improving lentil resilience.

## Materials and methods

### Plant material and field evaluation

A total of 142 lentil accessions, comprising 88 exotic lines, 12 cultivars, 34 ICARDA accessions and eight advanced breeding lines, were evaluated under field conditions over two consecutive winter seasons (2021–2022 and 2022–2023). The evaluations were conducted at research farms in Pusa (25°58′N, 85°32′E) and Dholi (25°59′N, 85°36′E), both of which are part of Dr. Rajendra Prasad Central Agricultural University, Bihar, India. Dholi exhibited higher relative humidity and soil moisture, favoring vegetative growth, while Pusa was comparatively drier (Table S1). Soil samples from both sites were analyzed for physicochemical properties. Both locations had sandy loam soils; however, Dholi soil showed lower bulk density (1.3 vs. 1.5 Mg m^−3^) and higher clay content (14.4 vs. 12.8%) compared with Pusa. Dholi also recorded greater availability of nitrogen (172.6 vs. 116.0 kg ha^−1^), potassium (121.3 vs. 77.0 kg ha^−1^) and sulfur (25.4 vs. 6.8 mg kg^−1^). In contrast, Pusa soil exhibited higher pH (8.8 vs. 8.2), organic carbon (0.6 vs. 0.4%), and electrical conductivity (0.5 vs. 0.3 dS m^−1^) than Dholi (Table S2).

The experiment was conducted using an augmented block design, with five popular varieties included as checks (Precoz, Sehore 74 − 3, K 75, L 4076 and L 4717). Among these, L 4717 is an early-maturing variety, Precoz is known for its high hundred-seed weight, and L 4076 is a high-yielding cultivar (Sharma et al. 2022). Sehore 74 − 3 is an early variety and susceptible check for wilt (Choudhary et al. 2022), while K 75 is a drought-tolerant variety (Dash et al. 2020). The experimental design consisted of four blocks, with each block containing 36 lentil accessions, except the fourth block, which included 34 accessions. All blocks were sown uniformly along with randomly replicated check varieties. Each accession was sown in a single two-meter-long row with a row-to-row spacing of 30 cm. Sowing was carried out in the first week of November, and harvesting took place in the first week of April each growing season. Phenotypic data were recorded for six traits, including morphological traits such as plant height, yield-related traits such as seed yield per plant, hundred-seed weight and number of pods per plant, and phenological traits such as days to 50% flowering and days to maturity. Morphological traits were measured on five randomly selected plants per accession, whereas phenological traits were recorded on a plot basis. Phenotypic data for the studied traits, previously published by Naik et al. (2024), were used for association analysis in this study. In total, three distinct phenotyping datasets were used for GWAS analysis: E1 (Dholi, 2021–2022), E2 (Dholi, 2022–2023), E3 (Pusa, 2022–2023). Additionally, across-environment BLUPs (best linear unbiased predictions) were computed for each trait using Meta-R software (Alvarado et al. 2020).

### DNA extraction and sequencing

DNA was extracted from fresh apical leaves of 15-day-old seedlings (collected from three plants representing each line) using the NucleoSpin^®^ 96 Plant II DNA extraction kit at the International Crops Research Institute for the Semi-Arid Tropics (ICRISAT), Hyderabad, Telangana, India. The concentration and quality of DNA were assessed using a Qubit 3.0 fluorometer (Thermo Fisher, United States) and agarose (0.8%) gel electrophoresis. Normalized DNA (12.5 ng µL^−1^) was used to prepare the DNA library using the Twist 96-Plex Library Preparation Kit (Twist Bioscience, United States). The 142 accessions were sequenced using the Illumina NovaSeq 6000 platform (Illumina, United States).

### SNP genotyping and filtration

The raw sequencing data were processed by removing adapter sequences and filtering out low-quality reads. Quality assessment was performed using FastQC (Version 0.12; Andrews 2010), and read trimming was conducted with Trimmomatic (Version 0.39; Bolger et al. 2014). The processed paired-end reads were aligned to the CDC Redberry Genome Assembly v2.0 (*Lcu.2RBY*; Ramsay et al. 2021) of lentil using BWA-MEM (Version 0.7.17) with default parameters (Li and Durbin 2009). Alignment results were converted into BAM format and unmapped or non-unique reads were removed using SAMtools (Version 1.19.2; Li et al. 2009). Variant calling was conducted using bcftools (Version 1.19; Li 2011) to identify single nucleotide polymorphisms (SNPs) based on the *Lcu.2RBY* reference genome (Ramsay et al. 2021). A minimum read depth of 3× per site was used as a threshold during variant calling to ensure reliability. Genotype imputation was performed using TASSEL (Version 5.0) with the LD KNNi Imputation plugin (Bradbury et al. 2007). To ensure data quality, SNPs were filtered based on three criteria: (i) > 5% missing data, (ii) minor allele frequency < 5% and (iii) heterozygosity > 40%.

### Population structure evaluation and linkage disequilibrium (LD) analysis

A genetic distance matrix was generated using SNPs to explore the genetic relationships among lentil accessions. A phylogenetic tree was then constructed using the Neighbor-Joining method to classify the accessions into distinct groups using TASSEL (Version 5.0). Population structure was analyzed using the admixture model in STRUCTURE software. For each value of K ranging from 1 to 10, thirty independent runs were performed. Each run included a burn-in period of 10,000 iterations, followed by 25,000 Markov Chain Monte Carlo (MCMC) replications after burn-in. The optimal number of populations (K) was determined following the method described by Evanno et al. (2005), and it was calculated using the “pop-helper” package in R (Francis 2016). Pairwise LD among high-quality SNPs was estimated as r^2^ using TASSEL. Principal component analysis (PCA) and the marker density map were generated using “GAPIT3” R package (Wang and Zhang 2021).

### Genome-wide association analysis

To explore the genetic factors associated with the six evaluated traits, a GWAS analysis was performed using the “GAPIT3” R package (Wang and Zhang 2021). This analysis employed two multi-locus models: BLINK and FarmCPU (Liu et al. 2016; Huang et al. 2019), using three principal components and a kinship matrix. BLUPs across three environments and individual datasets were used for GWAS to identify associations. The significance threshold for MTAs was determined using the Bonferroni correction (Johnson et al. 2010), with a cutoff of *p* ≤ 0.05/N, where N is the total number of SNPs. This Bonferroni correction controlled the genome-wide type I error rate at 5%. The “CMPlot” package (Yin 2019) was used to generate Manhattan and QQ plots displaying multiple GWAS results.

### Identification of stable MTAs using a single-locus model

The 49 unique SNPs were re-evaluated using a single-locus generalized linear model (GLM) across E1–E3 and the BLUP dataset to assess the consistency of signals across the environments. SNP data corresponding to the 49 unique associated SNPs identified from the initial GWAS (out of a total of 34,995 SNPs) were extracted for further analysis. The data were then re-analyzed by performing a GWAS using a single-locus model in GAPIT3. This approach allowed verification of whether the associations remained consistent under varying environmental conditions, thereby strengthening the reliability of the identified loci.

### Candidate gene identification

Genomic regions significantly associated with days to 50% flowering and other phenotypic traits were identified by filtering significant markers from the GWAS analysis. Candidate genes were identified within a 1 Mb region (500 kb upstream and 500 kb downstream of each significant SNP). This window allows for the inclusion of likely causal genes while reducing the likelihood of unrelated flanking genes, providing a practical balance between resolution and candidate gene coverage. The positions of these genes were determined using the annotated lentil genome available on JBrowse via the JGI Phytozome v13 database (https://phytozome-next.jgi.doe.gov/info/Lculinaris_v1).

## Results

### Phenotypic characterisation of lentil accessions

The analysis of variance (ANOVA) demonstrated highly significant differences (*P* < 0.01) among genotypes for all six traits. Field trials at Pusa and Dholi revealed a wide range of variability, especially for plant height, days to maturity and pods per plant (Table S3). Pearson correlation coefficient analysis revealed consistent relationships among the six measured traits across 142 lentil accessions. Days to maturity exhibited a strong positive correlation with days to 50% flowering (*r* = 0.75, *P* < 0.01). Seed yield per plant was positively correlated with pods per plant (*r* = 0.77, *P* < 0.01), while days to 50% flowering was negatively associated with seed yield per plant. Additionally, plant height exhibited positive associations with all traits except seed yield per plant. These medium-to-strong correlations suggest shared genetic bases, potentially due to pleiotropy or linkage. Based on the phenotypic performance several superior accessions were identified for specific traits. High-yielding accessions included EC 78438, EC 78498, EC 78408, P 13143, and EC 78491, all of which exceeded 2.7 g seed yield per plant, outperforming the check varieties Precoz (2.6 g) and K75 (1.9 g). Early flowering accessions such as EC 223197-A, P 8112, EC 267696, EC 78532, and EC 223242 were notable, with EC 223197-A reaching 50% flowering in 67 days, one day earlier than the check variety L 4717. For days to maturity, accessions including EC 267696, P 3235, EC 267641, EC 78933, and EC 78551-A matured between 109 and 112 days, while L 4717 matured in 102 days. Importantly, EC 78491 and EC 78438 combined early flowering with high seed yield per plant, making them particularly promising candidates. Detailed phenotypic data and their interpretation have been reported in our earlier study (Naik et al. 2024).

### Sequencing data and genome-wide SNP distribution in lentil

**Fig. 1:**
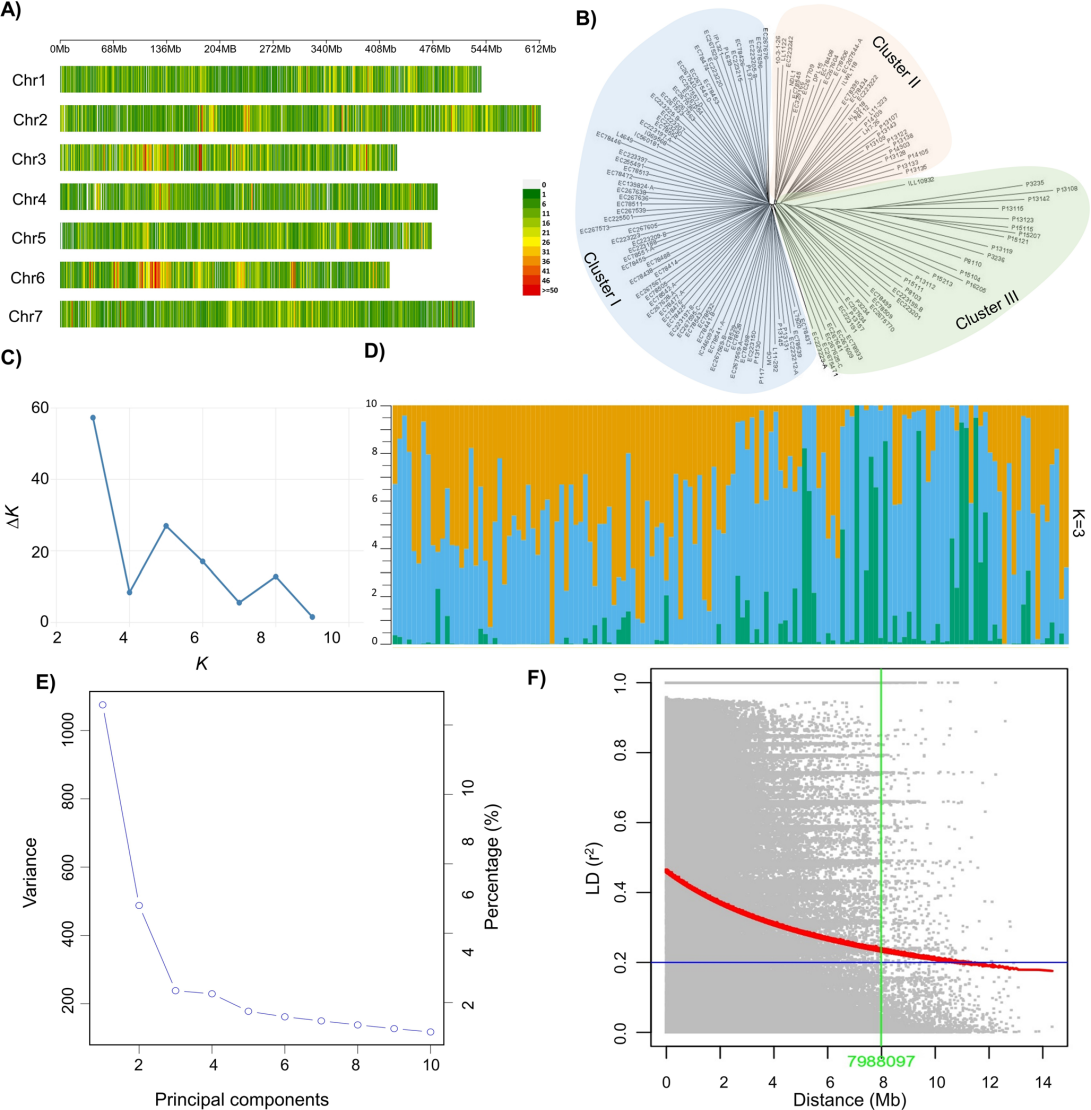
Genomic diversity and population structure of lentil accessions based on genome-wide SNP markers. **A** Genome-wide SNP distribution illustrating the number of SNPs within 1 Mb windows. Chromosomes are arranged horizontally, with SNP density represented by the scale on the right. **B** Unrooted neighbor joining tree based on a distance matrix in TASSEL. **C** The Delta K (ΔK) plot was calculated for K values ranging from 2 to 10. **D** Population structure classification based on membership probability (Q-values). In the admixture plot, each vertical bar represents an individual accession, and the different colored segments within each bar indicate the proportion of the genome assigned to each of the identified subpopulations. **E** Scree plot explaining the variance of principal components, indicating significance of first three components. **F** Linkage disequilibrium (LD) decay plot depicts the LD patterns across the chromosomes

The genomes of 142 lentil accessions were sequenced and generated 572.1 Gb of raw sequence data. On average, each accession generated 29 million reads. The read lengths ranged 131–143 bp (average 137 bp). Overall sequencing quality was high, with 96.5% of bases exhibiting Phred scores ≥ Q20 and 91.3% ≥ Q30 (Table S4). The average depth-of-coverage was approximately 11.7× for the lentil accessions. After stringent filtering, 34,995 high-quality SNPs were identified. Their distribution across the lentil genome was analyzed to assess chromosome-wise genetic variation. Chromosome 2, the longest at 613.6 Mb, harbored 6,055 SNPs, representing 17.3% of the total variants, with a SNP density of 101 per Mb (Fig. 1A; Table S5). In contrast, Chromosome 4, despite being shorter at 481.9 Mb, displayed the highest variant rate of 114.0 SNPs/Mb, contributing 12.1% of the total SNPs. On average, the variant rate across the genome was 100 SNPs/Mb (Table S5).

### Genetic diversity, population structure and LD decay

The Neighbor-Joining tree analysis revealed that the 142 lentil accessions were broadly divided into three major clusters: Cluster I, Cluster II and Cluster III (Fig. 1B; Table S6). Additionally, the population structure of 142 lentil accessions revealed that the highest delta K (ΔK) value occurred at K = 3, suggesting this as the most probable number of populations, with K = 5 being the next likely option (Fig. 1C). The accessions of the three subpopulations were classified as either pure or admixed based on genetic similarity within each subpopulation. Individuals with a similarity level of ≥ 0.8 were categorized as pure, while those below this threshold were considered admixed. Among the accessions analyzed, 100 exhibited genomic admixtures, while the remaining 42 were classified as pure. The admixture plot illustrated subpopulation structure based on the origin and nature of the accessions (Fig. 1D). The PCA results revealed the genetic structure of the population, with the first three principal components (PC1: 10.5%, PC2: 4.8% and PC3: 2.3%) explaining 17.7% of the total genetic variation. The scree plot indicates the variance contribution of each principal component, with the first few components capturing the most significant variation, suggesting distinct genetic subgroups within the population (Fig. 1E). The maximum LD (r^2^) observed was 0.4. Genome-wide LD analysis showed relatively slow decay, with r^2^ declining below 0.2 at an average physical distance of 7.9 Mb (Fig. 1F). LD decay varied among chromosomes, ranging from ~ 2.0 Mb (Chromosome 1) to ~ 11.5 Mb (Chromosome 2).

### Genome-wide association analysis

**Fig. 2:**
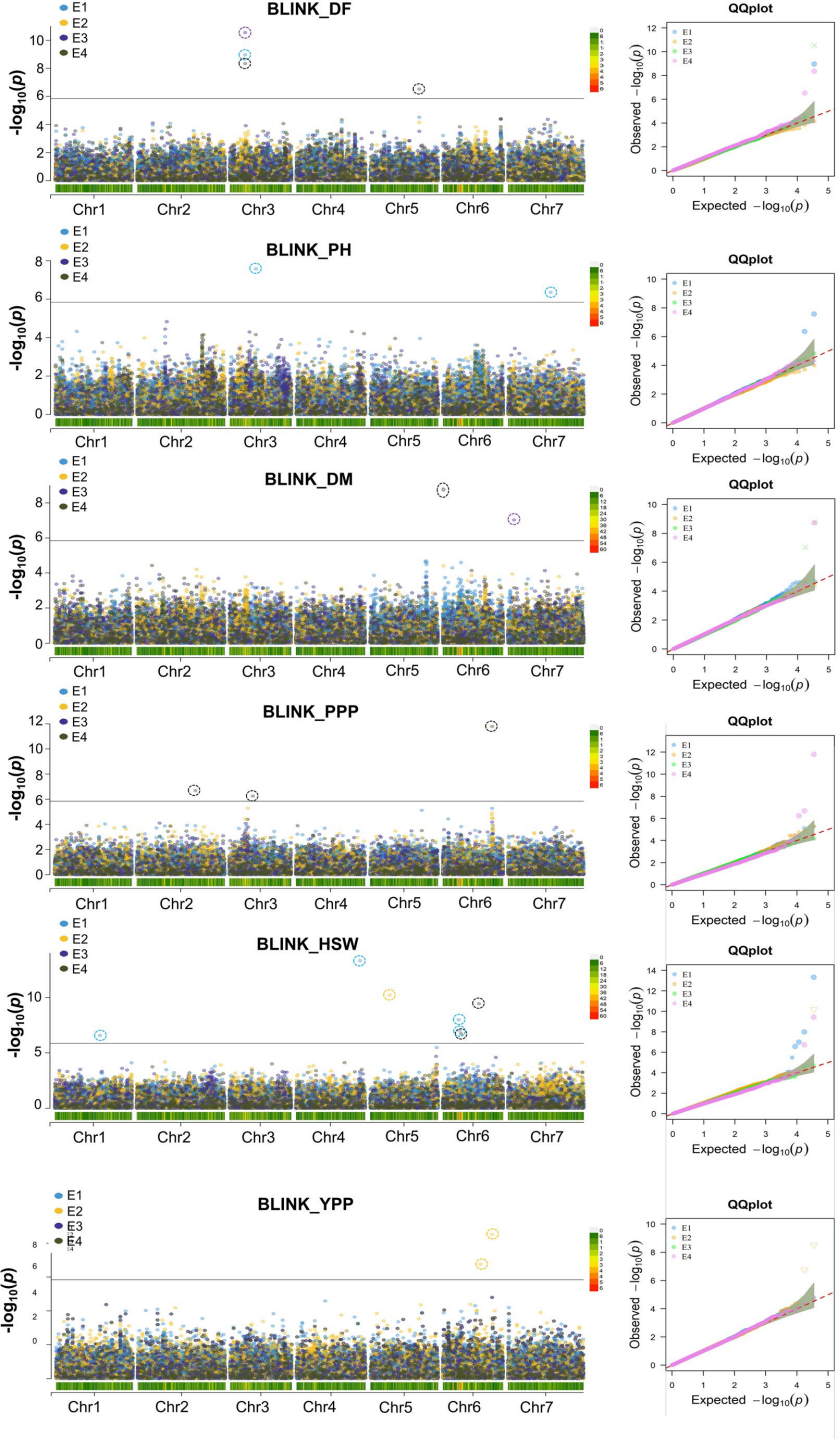
Genome-wide association signals for six traits using the BLINK model. Manhattan plots display significant SNPs associated with the traits across four datasets: E1 (Dholi 2022), E2 (Dholi 2023), E3 (Pusa 2023) and BLUP. In a Manhattan plot, each dot represents a single SNP, with its genomic position plotted along the x-axis and its corresponding statistical significance (−log10 of the p-value) plotted on the y-axis. The color of the dots indicates the dataset from which the SNP was identified. The horizontal line represents the Bonferroni threshold for significant associations. Quantile–quantile (QQ) plots for each trait are displayed next to the corresponding Manhattan plots. Plant height (PH), seed yield per plant (YPP), days to 50% flowering (DF), days to maturity (DM), pods per plant (PPP) and hundred seed weight (HSW)

**Fig. 3:**
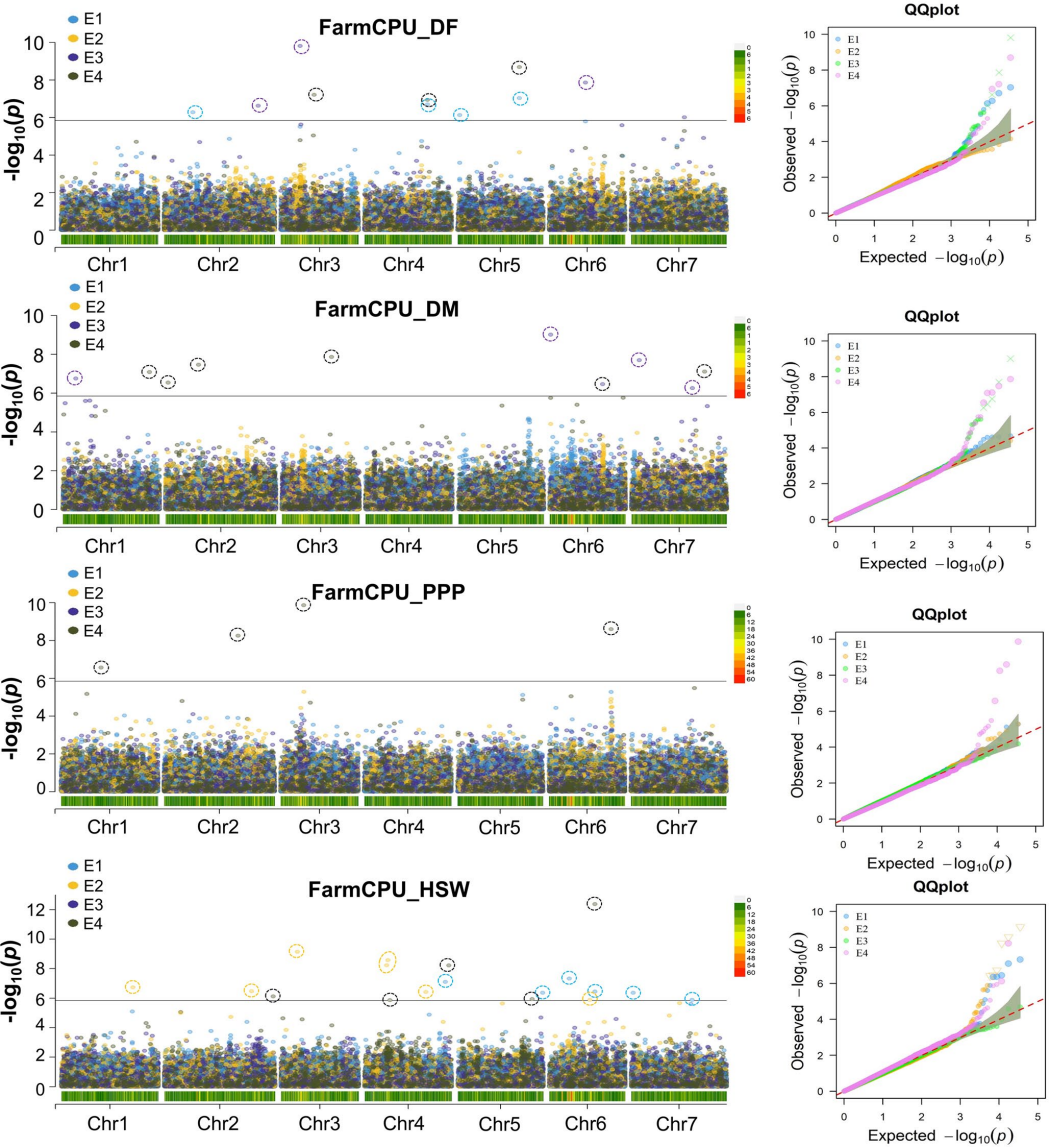
Genome-wide association signals for four traits using the FarmCPU model. Manhattan plots display significant SNPs associated with the traits across four datasets: E1 (Dholi 2022), E2 (Dholi 2023), E3 (Pusa 2023) and BLUP. In a Manhattan plot, each dot represents a single SNP, with its genomic position plotted along the x-axis and its corresponding statistical significance (−log10 of the p-value) plotted on the y-axis. The color of the dots indicates the dataset from which the SNP was identified. The horizontal line represents the Bonferroni threshold for significant associations. Quantile–quantile (QQ) plots for each trait are displayed next to the corresponding Manhattan plots. Days to 50% flowering (DF), days to maturity (DM), pods per plant (PPP), hundred seed weight (HSW)

A Bonferroni-corrected significance threshold of *p* ≤ 1.4 × 10^−6^ (–log_10_(*p*) ≥ 5.8) was applied to identify significantly associated SNPs in the GWAS. In total, 64 significant MTAs were identified for six traits evaluated across three environments and using BLUP estimates. Across all environments, BLINK identified the fewest MTAs (21; 32.8%; Fig. 2), whereas FarmCPU detected the most (43; 67.2%; Fig. 3; Table S7). In individual environments, 17, 10, and 12 MTAs were identified in E1, E2 and E3, respectively (Table S7), with most MTAs being specific to individual environments across all models. Using BLUP datasets, 25 MTAs were identified (Table 1). Importantly, SNPs Chr2_410777988 (pods per plant), Chr5_342836807 (days to 50% flowering), Chr6_253032265 (hundred seed weight), and Chr6_344313403 (pods per plant) were consistently detected by both BLINK and FarmCPU models, explaining 1.7–66.2% of the phenotypic variance.

**Table 1:** Significant SNPs associated with phenological and yield-related traits in lentil using BLUP datasets and multi-locus GWAS models

Trait	SNP	*P*-value	MAF	PVE (%)	Model
Days to 50% flowering	Chr3_106842007	4.55 × 10^− 09^	0.41	43.55	BLINK
	Chr3_184872087	6.18 × 10^− 08^	0.19	6.92	FarmCPU
	Chr4_348520593	1.13 × 10^− 07^	0.10	2.41	FarmCPU
	Chr5_342836807	2.04 × 10^− 09^	0.27	34.68	Both
Days to maturity	Chr1_482512926	8.20 × 10^− 08^	0.07	1.01	FarmCPU
	Chr2_13220114	2.85 × 10^− 07^	0.05	1.00	FarmCPU
	Chr2_182480268	3.43 × 10^− 08^	0.42	2.58	FarmCPU
	Chr3_274815239	1.37 × 10^− 08^	0.07	1.00	FarmCPU
	Chr6_4338291	1.86 × 10^− 09^	0.07	82.76	BLINK
	Chr7_407008237	7.83 × 10^− 08^	0.06	10.87	FarmCPU
Hundred seed weight	Chr2_603974724	7.64 × 10^− 07^	0.33	14.87	FarmCPU
	Chr4_139205447	1.36 × 10^− 06^	0.14	1.10	FarmCPU
	CHR4_466290148	6.05 × 10^− 09^	0.32	35.95	FarmCPU
	Chr5_413492767	1.15 × 10^− 06^	0.39	1.07	FarmCPU
	Chr6_128041250	1.90 × 10^− 07^	0.23	30.04	BLINK
	Chr6_253032265	4.20 × 10^− 13^	0.15	47.69	Both
Pods per plant	Chr1_221418298	2.72 × 10^− 07^	0.24	1.01	FarmCPU
	Chr2_410777988	5.62 × 10^− 09^	0.25	7.40	Both
	Chr3_126687055	1.37 × 10^− 10^	0.47	1.04	FarmCPU
	Chr3_163495081	5.61 × 10^− 07^	0.31	8.23	BLINK
	Chr6_344313403	1.60 × 10^− 12^	0.07	66.17	Both

Bayesian information and linkage-disequilibrium iteratively nested keyway (BLINK); Fixed and random model circulating probability unification (FarmCPU); Minor allele frequency (MAF); Phenotypic variance explained (PVE); single nucleotide polymorphism (SNP). The marker name indicates the chromosome number and physical position of the MTAs. MTAs identified in both models are presented with high PVE and significant *P*-values

#### MTAs for phenological and morphological traits

Fifteen significant MTAs associated with days to 50% flowering were detected across all chromosomes except chromosome 1. The FarmCPU model identified 11 MTAs, while BLINK detected four. Notably, the MTA Chr3_106842007 (PVE = 43.5%) was consistently identified in the E1, E3, and BLUP datasets by both models, and Chr5_342836807 (PVE = 34.7%) was also common to both on chromosome 5. Similarly, thirteen significant MTAs associated with days to maturity were identified across both the E3 and BLUP datasets. The FarmCPU model identified ten MTAs, while BLINK detected three. Two associations, Chr6_4338291 (PVE = 82.7%) and Chr7_42734754 (PVE = 3.9%), were consistently identified by both models on chromosomes 6 and 7, respectively. For the morphological trait plant height, only two significant MTAs were detected on chromosomes 3 and 7, represented by SNPs Chr3_181072527 (PVE = 35.0%) and Chr7_297717438 (PVE = 14.1%) in the E1 dataset. These associations were detected exclusively by the BLINK model, and no significant SNPs associated with plant height were identified in the E2, E3, or BLUP datasets. This pattern is likely due to low cross-environment correlations, indicating substantial genotype × environment (G × E) interaction and environmental influence on the trait, which reduces the consistency of SNP associations across different environments.

#### MTAs for yield-related traits

Genome-wide association analysis for yield-associated traits across multiple environments identified 34 statistically significant MTAs. Of these, 25 MTAs were associated with hundred seed weight, with seven detected by the BLINK model and 18 by the FarmCPU model. A large number of MTAs were located on chromosomes 4 and 6, with seven and eight MTAs, respectively. All these MTAs were identified using the E1, E2 and BLUP datasets, while no MTAs for hundred seed weight were detected in the E3 dataset. Additionally, two associations (Chr4_447360607 and Chr6_253032265) were common to both models on chromosomes 4 and 6. For pods per plant, a total of seven MTAs were identified on chromosomes 1, 2, 3 and 6 from the combined dataset (BLUP). The BLINK model detected three MTAs, whereas the FarmCPU model identified four. The PVE by these MTAs ranged from 1.0% (Chr3_126687055) to 66.2% (Chr6_344313403), indicating that multiple loci with varying effects contribute to variation in pods per plant. Additionally, for seed yield per plant, only two MTAs (Chr6_262648494 and Chr6_344313403) were identified, both from the E2 dataset and exclusively detected by the BLINK model on chromosome 6. The locus Chr6_344313403, identified for both pods per plant and seed yield per plant, showed a pleiotropic effect.

### Re-evaluation of stable MTAs using a single-locus model

**Fig. 4:**
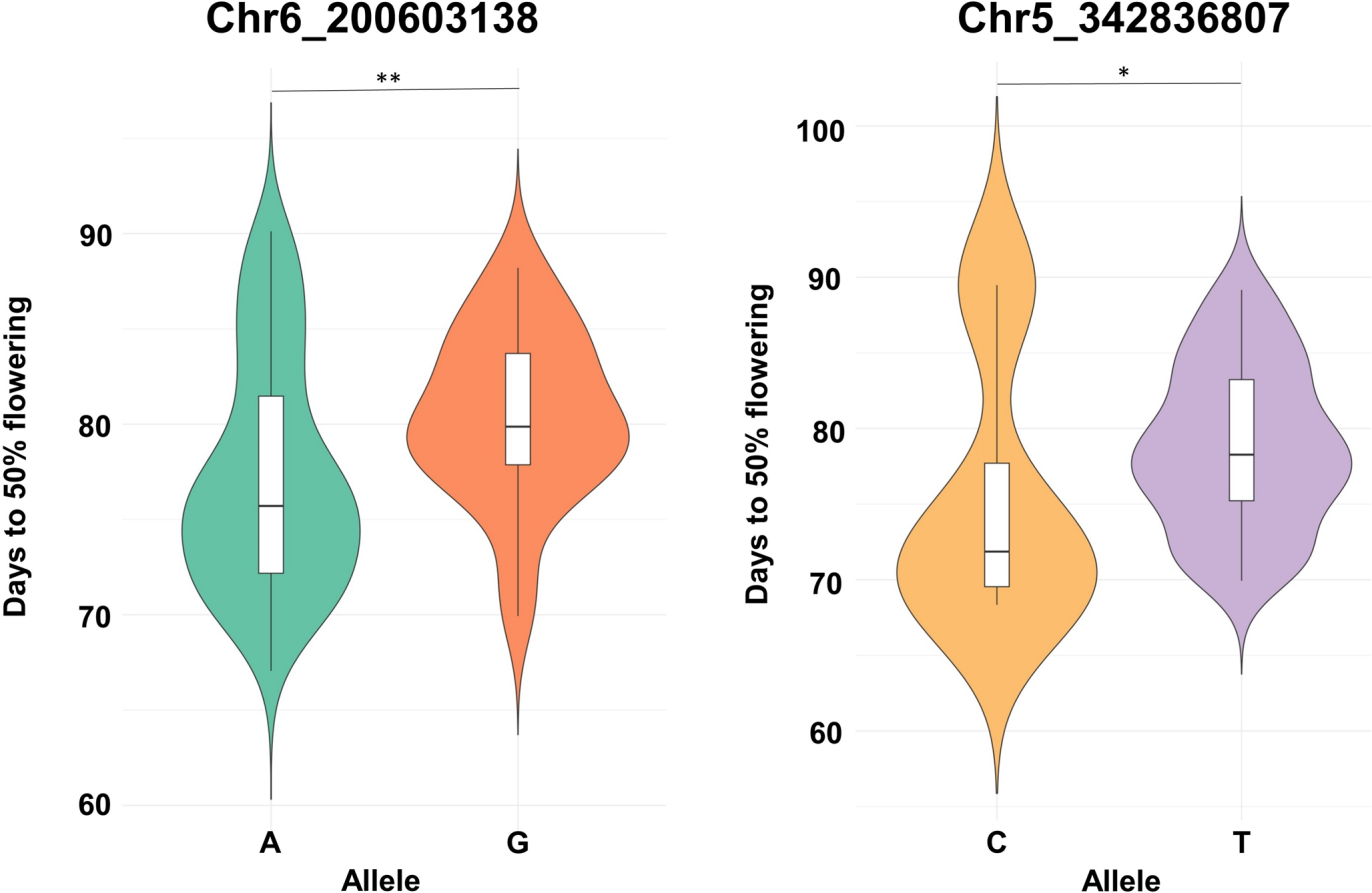
Violin plots depicting the allelic effects of SNPs associated with days to 50% flowering, identified using the GLM approach. These associations were consistent across all environments, and the phenotypic data shown in the graph are based on combined BLUP values. *Significant at the 5% probability level; **Significant at the 1% probability level

GWAS analysis identified a total of 64 MTAs across all four datasets. Of these, 49 were considered unique, meaning that repeated occurrences of the same MTA across environments or models were counted only once. The 49 SNPs were tested for consistency using a single-locus GLM model. This analysis revealed that two MTAs (Chr5_342836807 and Chr6_200603138) were consistently detected across all environments (E1, E2, E3 and BLUPs) for days to 50% flowering (Table S8). SNP Chr5_342836807 on chromosome 5, exhibited a PVE ranging from 5.7% in E3 to 21.3% in E2, whereas SNP Chr6_200603138 on chromosome 6 showed a PVE of 13.9% in E2 to 19.7% in the BLUP dataset. Allelic effects based on combined BLUP values revealed loci with statistically significant differences (Fig. 4).

### Candidate genes associated with significant MTAs

The 49 unique MTAs were subsequently analyzed to identify putative candidate genes based on their physical positions and functional annotations. Within a genomic window of 1 Mb, 570 genes were identified across 49 MTAs (Table S9). Wide variation was observed in the number of genes identified within the genomic regions, ranging from 1 to 36 genes per MTA, with an average of approximately 11 genes per MTA. Among all the identified genes, a substantial proportion (*n* = 116; 20%) encodes uncharacterized proteins, highlighting the need for further functional annotation and validation. The remaining genes predominantly contribute to critical biological processes essential for plant growth and development.

#### Candidate genes for phenological and morphological traits

The genomic region associated with days to maturity on chromosome 3 (marker: Chr3_274815239) contains key putative candidate genes such as *Lcu.2RBY.3g041640* (encoding Glutaredoxin C1), *Lcu.2RBY.3g041700* (encoding the *MYB98* transcription factor) and *Lcu.2RBY.3g041620* (encoding a Polygalacturonase/pectinase). Similarly, the genomic region on chromosome 1 linked to marker Chr1_482512926 includes the genes *Lcu.2RBY.1g065700* and *Lcu.2RBY.1g065710* (cinnamyl-alcohol dehydrogenase, CAD) and *Lcu.2RBY.1g065790* (transcription factor EMB1444-related). On chromosome 2, the genomic region associated with marker Chr2_13220114 for days to maturity harbors genes such as *Lcu.2RBY.2g006060* (encoding a Dof zinc finger transcription factor), *Lcu.2RBY.2g006120* (encoding a GT-2 trihelix transcription factor) and *Lcu.2RBY.2g006210* (encoding an LRR receptor-like kinase). Another genomic region on chromosome 2 (marker: Chr2_182480268) includes *Lcu.2RBY.2g033950* (encoding a serine/threonine protein kinase), *Lcu.2RBY.2g034020* (encoding a leucine-rich repeat protein) and *Lcu.2RBY.2g034030* (encoding a pentatricopeptide repeat protein). Additionally, on chromosome 6, the genomic region (marker: Chr6_4338291) for days to maturity spans a region of 35 genes, including key putative candidates such as *Lcu.2RBY.6g001060* (Legumain), *Lcu.2RBY.6g001150* (MYB transcription factor), *Lcu.2RBY.6g001330* (SAUR family protein), *Lcu.2RBY.6g001340* (SWEET15 sugar transporter), and *Lcu.2RBY.6g001070* (High mobility group B protein 10-related), which may collectively influence developmental processes and flowering regulation.

An MTA on chromosome 2 (Chr2_521877344) was associated with a genomic region containing *Lcu.2RBY.2g079110*, which encodes a tetratricopeptide repeat (TPR) protein. Furthermore, on chromosome 7, MTAs for days to 50% flowering were identified. This genomic region contains two important genes: *Lcu.2RBY.7g039780* (encoding a NAC transcription factor-like protein) and *Lcu.2RBY.7g039820* (encoding a GRAS domain transcription factor). Using the GLM model, two MTAs were identified across all the environments. The SNP Chr6_200603138, associated with days to 50% flowering, is located in a genomic region containing four genes: *Lcu.2RBY.6g025870* (ribosomal protein L10e/L16), *Lcu.2RBY.6g025900* (dolichol-phosphate mannosyltransferase subunit 1), *Lcu.2RBY.6g025940* (mitochondrial phosphate transporter, SLC25 family) and *Lcu.2RBY.6g025820* (uncharacterized protein). Similarly, within the genomic window around marker Chr5_342836807, ten genes were identified. Among them, UDP-glycosyltransferases (*Lcu.2RBY.5g047160* and *Lcu.2RBY.5g047180*) and the RNA-binding motif protein (*Lcu.2RBY.5g047190*) are strong candidates likely involved in flowering regulation. The genomic region on chromosome 3 (Chr3_181072527) associated with plant height contains thirteen genes, including *Lcu.2RBY.3g026810* (β-D-glucan exohydrolase-like protein) and *Lcu.2RBY.3g026860* (MYB-like DNA-binding protein). Similarly, the genomic region on chromosome 7 linked to marker Chr7_297717438 contains eight genes, including *Lcu.2RBY.7g039990* (GRF zinc finger protein), *Lcu.2RBY.7g040020* and *Lcu.2RBY.7g040060* (aspartyl protease family proteins) and *Lcu.2RBY.7g040040* (Rho/Rac/CDC GTPase-activating protein).

#### Candidate genes for yield-related traits

The MTA (Chr6_344313403) identified on chromosome 6 is associated with two yield-related traits (seed yield per plant and pods per plant). This genomic region contains important yield-related genes, such as *Lcu.2RBY.6g050520* (serine/threonine protein kinase NEK5), *Lcu.2RBY.6g050590* (RNA polymerase subunit RPB6) and *Lcu.2RBY.6g050640* (bHLH transcription factor BHLH4). The genomic region associated with SNP Chr7_9794560 includes the gene *Lcu.2RBY.7g005330*, which encodes a MYB transcription factor. Additionally, on chromosome 4, the MTA (Chr4_338897743) for hundred seed weight spans a region of 21 genes, with key putative candidates including *Lcu.2RBY.4g050610* (Asparagine synthase), *Lcu.2RBY.4g050620* (L-ascorbate oxidase), and *Lcu.2RBY.4g050710* (Pyruvate kinase), which may play crucial roles in seed development and metabolic regulation.

## Discussion

Advancements in cost-effective and high-throughput SNP genotyping techniques, along with the growing availability of genomic resources, have made genome-wide association studies a powerful tool for identifying trait associated alleles in various crop species, including legumes. In this study, the natural genetic diversity within lentil accessions was explored to identify MTAs. The selected accessions comprising exotic lines, cultivars, ICARDA accessions and advanced breeding lines (Naik et al. 2024) were chosen to maximize genetic variation. By incorporating a diverse range of alleles for agronomic and other key traits, these accessions provided a robust foundation for identifying important loci linked to desirable characteristics. A comparative analysis of field experiments at Pusa and Dholi revealed substantial phenotypic variation across all traits (Table S3, Fig. S1). The comparative analysis of environmental and soil conditions at Dholi (2021–22) and Pusa–Dholi (2022–23) highlights distinct differences that could influence crop growth and productivity. Climatic data indicate that Dholi generally experienced higher relative humidity (up to 89%) and root zone soil water content (0.6–0.7 m^3^/m^3^), along with slightly higher maximum and minimum temperatures, compared to Pusa, which was drier and cooler. Additionally, Dholi and Pusa had sandy loam soils, but Dholi had lower bulk density, more clay, and higher nutrients, while Pusa had more organic carbon and conductivity. These conditions suggest that Dholi provided a more favorable microclimate for vegetative growth, whereas the relatively lower moisture at Pusa may have accelerated reproductive development.

### Population genetic diversity and structure analysis

Genetic diversity and population structure play a pivotal role in understanding the history of domestication and genetic relationships among accessions. LD analysis revealed a slow decay pattern in the lentil genome, indicating the presence of large haplotype blocks. Such extended LD is characteristic of self-pollinated crops like lentil and is likely a consequence of domestication history, limited effective recombination, genetic drift, population stratification and breeding-related bottlenecks (Flint-Garcia et al. 2003). For instance, Srungarapu et al. (2022) reported an LD decay of 4.0 Mb in chickpea (*Cicer arietinum*). In lentil, previous studies have reported contrasting LD decay distances, ranging from 1 Mb (Kumar et al. 2025) to 0.3 Mb (Ma et al. 2020), reflecting differences in population structure and recombination history. Despite this slow LD decay, the marker density used was adequate for detecting trait-associated genomic regions. An average density of ~ 100 SNPs per Mb, resulting in many markers (700–800) within each LD block. Although slow LD decay restricts fine-scale gene resolution, the marker density employed is sufficient for robust association mapping. Among these, population stratification is a key factor impacting the precision and reliability of GWAS findings. In this study, population structure analysis using STRUCTURE, phylogenetic analysis, and PCA consistently identified three distinct subpopulations, revealing the genetic makeup of the lentil accessions. The population structure analysis further suggested that lentil accessions could be categorized based on their nature of accessions, such as exotic lines, landraces and modern varieties. The smaller number of significant clusters may be attributed to the shared geographical origin of most accessions in this study and their closely related ancestral history. Similarly, Khazaei et al. (2016) reported three subpopulations using a large set of lentil accessions from over fifty countries, categorized based on the climatic regions where lentil is widely cultivated. Additionally, previous studies have reported between three and five ancestral subpopulations in lentil (Singh et al. 2019; Rajendran et al. 2021). These consistent patterns underscore the importance of genetic diversity in shaping the development and improvement of lentil cultivars and offer valuable information for future breeding programs to enhance lentil traits.

### Genetic loci associated with key traits

A single-environment GWAS approach alone is insufficient to fully unravel the genetic mechanisms regulating complex traits such as flowering time in lentil. To overcome this limitation, the power of GWAS analysis was enhanced by utilizing phenotypic data derived from multi-environment trials. The traits analyzed in this study included agronomic traits and phenological traits, which are globally significant targets for lentil breeding programs. Previous GWAS studies, which used lentil reference set accessions, investigated similar traits: days to flowering, days to maturity and hundred seed weight and identified MTAs (Rajendran et al. 2021; Neupane et al. 2022). In contrast, our study employed different accessions, a statistically robust multi-locus GWAS model.

Multi-environment trials are typically combined to capture overall phenotypic variation in GWAS for detecting MTAs. However, in diverse accessions, phenotypic variation across different environments can be utilized to assess trait plasticity or trait G × E interactions using appropriate statistical models. Identifying environmentally stable and environment-specific MTAs enhances our understanding of the genetic basis of trait G × E interactions and contributes to knowledge of the genetic architecture of important traits (Zhao et al. 2022). The use of BLUP values helps account for environmental variation, providing more reliable estimates of genetic effects (Pezeshkpour et al. 2025). Additionally, in individual environmental analyses, a higher number of site-specific MTAs were identified compared to shared MTAs, likely due to the relatively low cross-environment correlations observed for some traits, particularly yield. Such low correlations indicate strong G × E interactions, where allelic effects vary under different environmental conditions (Li et al. 2025). For example, only two MTAs for plant height were detected in a single environment, suggesting that environmental variability and small-effect loci reduced the power of GWAS to detect consistent associations across datasets. Similar trends have been reported in other legume crops, where yield-related loci exhibited strong environmental dependency (Gutierrez et al. 2024; Chandana et al. 2024). Although the major climatic conditions such as temperature and rainfall are similar at both locations, micro-environmental conditions differed significantly (Table S2). Fine-scale heterogeneity in topography, nutrient availability, water retention and other environmental conditions contributed to the formation of distinct microhabitats (Denney et al. 2020). These micro-environmental variations, combined with plant plasticity, can substantially influence flowering time and yield (Alseekh et al. 2025). In this study, considerable variation in trait performance was observed across both locations. Notably, plants grown at Pusa showed reduced performance in terms of plant height, yield, and physiological traits compared to those at Dholi. This variability may explain why the multi-locus models did not consistently identify MTAs across locations, whereas the single-locus model was able to detect consistent MTAs.

Several consistently detected MTAs were identified by integrating results from multi-locus (BLINK and FarmCPU) and single-locus (GLM) GWAS analyses. Consistent loci were defined as SNPs associated with the same trait across multiple environments or models or associated with more than one trait within a close genomic region. Notably, SNPs such as Chr3_106842007 (days to 50% flowering), Chr6_4338291 (days to maturity), Chr6_344313403 (pods per plant and seed yield per plant), and Chr7_42734754 (days to maturity and plant height) were consistently detected across multiple models and environments, indicating stable and robust associations. Similarly, single-locus GLM analysis consistently detected MTAs such as Chr2_410777988 (pods per plant and seed yield per plant) and Chr5_342836807 (days to 50% flowering), further validating the multi-locus results. The phenotypic correlation analysis supported these genomic findings, revealing consistent relationships among the measured traits across lentil accessions.

The strength of this study is reflected in the identification of a greater number of key MTAs compared to previous lentil studies. On chromosome 3, two MTAs for days to 50% flowering were identified (Chr3_106842007 and Chr3_274815239). Similarly, previous lentil GWAS studies also reported MTAs for days to 50% flowering on the same chromosome (Rajendran et al. 2021; Neupane et al. 2022). Chr3_106,842,007 is approximately 9.3 Mb away from the previously reported flowering association SLCCHR3_116163111, suggesting partially overlapping (Rajendran et al. 2021). In this study, MTAs closer than 7.9 Mb are considered overlapping, and those less than 10 Mb apart are partially overlapping. A recent study using the QTL-seq approach identified three QTLs (*LcqDTF3.1*,* LcqDTF3.2* and *LcqDTF3*) on chromosome 3 for days to 50% flowering (Shivaprasad et al. 2024). The MTA Chr3_184872087 for days to 50% flowering co-localized with a previously reported senescence-associated MTA on chromosome 3, lying only 0.9 Mb apart, indicating a potential pleiotropic genomic region (Lorenzetti et al. 2024). On the same chromosome, the flowering-associated MTA (Chr3_184,872,087) and the maturity-associated MTA (Chr3_274,815,239) were located approximately 162 Mb and 72 Mb away, respectively, from the reported *LcqDTF3.1* locus (Shivaprasad et al. 2024). Indicating that these MTAs likely represent novel loci distinct from the previously reported QTL. The associations for hundred seed weight (Chr4_466,290,148) and pods per plant (Chr6_344,313,403) in our study are located 23.6 Mb and 30.0 Mb away, respectively, from previously reported anthracnose race 1 resistance loci (Gela et al. 2021). Present study not only confirms several previously reported MTAs for key traits but also identifies potentially novel loci, providing valuable targets for future functional validation and breeding programs.

### Candidate genes for flowering time and yield-related traits

Although the LD decay distance in our study was approximately 7.9 Mb, using such a wide window would encompass many genes, potentially diluting the resolution and complicating the identification of true candidate genes. Therefore, to improve specificity and reduce the likelihood of false positives, a smaller and more practical window size of 1 Mb was selected for putative candidate gene identification. A similar 1 Mb genomic region was also used for candidate gene analysis in a recent lentil study (Kumar et al. 2025). All identified SNPs were located within intragenic regions. In the present study, several genomic regions associated with MTAs contained genes encoding *MYB* transcription factors (Table S9), which are known to play pivotal roles in regulating diverse biological processes (Ambawat et al. 2013). Previous studies have shown that MYB transcription factors influence inflorescence development in chickpea (Caballo et al. 2022), whereas in lentil, they are associated with both flowering time and plant height (Dutta et al. 2022). Several MYB transcription factors have been reported to regulate flowering by integrating light and circadian signals, and their overexpression often leads to delayed flowering (Zhu et al. 2020). The genomic region (Chr3_106842007) contains the gene *Lcu.2RBY.3g016910*, which encodes a MADS-box transcription factor. MADS-box genes are well known regulators of flowering time and vernalization responses, and previous studies have implicated members of this family in the control of reproductive phase transitions (Schilling et al. 2018). In lentil, MADS-box transcription factors have also been reported to influence flowering time (Shivaprasad et al. 2024). The SNP Chr6_200603138 is located within a genomic region containing genes potentially involved in flowering, including *Lcu.2RBY.6g025900*, which encodes dolichol-phosphate mannosyltransferase. This gene has been reported to participate in protein glycosylation in meristems and to influence flowering regulation (Cho et al. 2017). Another gene in this region, *Lcu.2RBY.6g025940*, a mitochondrial phosphate transporter, affects ATP production and gibberellin metabolism, both of which are critical for flower initiation and development (Zhu et al. 2012; Mutasa-Göttgens and Hedden 2009). The genomic region (Chr4_348520593) associated with days to flowering, contains *Lcu.2RBY.4g052390*, which encodes *Abscisate beta-glucosyltransferase*, an enzyme involved in abscisic acid metabolism that regulates floral transition and stress-responsive flowering (Wang et al. 2012). The gene *Lcu.2RBY.2g079110* encodes a tetratricopeptide repeat protein, closely related to AT PRP39-1 in *Arabidopsis thaliana*, which is known to regulate flowering time (Wang et al. 2007). Genes encoding pentatricopeptide repeat (PPR) domain proteins were identified in multiple genomic regions associated with MTAs (Chr2_182480268, Chr2_603974724, Chr4_466290148 and Chr5_413492767), highlighting their role in diverse biological processes. PPR proteins play essential roles in organellar gene expression and RNA metabolism, influencing flowering and fertility (Barkan and Small 2014). The candidate gene *Lcu.2RBY.7g039820* (GRAS transcription factor) likely regulates flowering, as GRAS proteins act as transcriptional regulators involved in development, gibberellic acid signaling and light responses across diverse plant species (Hirsch and Oldroyd 2009). A recent study in lentil found that 50 *LcGRAS* genes were identified across nine subfamilies and seven chromosomes, with several, including *LcGRAS04*,* LcGRAS11*, and *LcGRAS25*, strongly upregulated under abiotic stresses, highlighting their role in stress tolerance (Ali et al. 2026). The NAC transcription factor (*Lcu.2RBY.7g039780*) is a member of the NAC gene family, which has been reported to play important roles in transcriptional reprogramming associated with stress responses in legume plants (Peng et al. 2009). Furthermore, its association with light, hormone, and energy signaling pathways implicates extensive crosstalk between stress adaptation and overall plant fitness (Srivastava and Sahoo 2022).

The co-localized genomic region (Chr6_344313403) associated with two yield-related traits, contains important genes, including serine/threonine protein kinase *NEK5* and a *bHLH* transcription factor. This result is supported by previous studies showing that BHLH transcription factors regulate nutrient uptake and plant development, highlighting their potential role in yield regulation in lentil (Gupta et al. 2017). Additionally, *Lcu.2RBY.2g094210* near Chr2_603974724 encodes a seed linoleate 9 S-lipoxygenase-3 (*9 S-LOX*). Lipoxygenases are iron-containing enzymes that catalyze the oxygenation of linoleic acid, generating oxylipins, bioactive compounds essential to plant defense and development (Loiseau et al. 2001). In seeds, *9 S-LOXs* produce 9 S-hydroperoxy-octadecadienoic acid, which acts as a precursor for defense-related signaling molecules (Singh et al. 2022). Although specific studies on *Lcu.2RBY.2g094210* in lentil are limited, its orthologs in other species suggest vital functions in seed physiology, including germination, storage and stress tolerance. The roles of these identified genes are supported by functional evidence from other species, but experimental validation in lentil is essential to confirm their contributions. They represent valuable targets for functional studies, marker-assisted selection and gene editing, aimed at accelerating the development of improved lentil cultivars.

## Conclusion

A GWAS analysis was performed using lentil accessions and multi-locus models, incorporating both individual phenotypic values and BLUP-derived estimates across all traits. This comprehensive analysis identified 64 MTAs, including 25 based on combined BLUP values. The application of high-resolution GWAS models (FarmCPU and BLINK) demonstrated strong statistical power, enabling the precise detection of significant MTAs that can accelerate breeding strategies aimed at improving lentil yield and adaptation. Further annotation of these loci revealed candidate genes potentially involved in the molecular regulation of key agronomic traits. Notably, the MYB transcription factor family emerged as a central regulator of flowering time, plant height, seed weight and pod number, underscoring its broader significance in plant growth and productivity. Upon validation, these candidate genes and associated SNPs will serve as valuable genetic resources. They provide a robust foundation for trait optimization through marker-assisted selection and genomic breeding.

## Supplementary Information

Below is the link to the electronic supplementary material.

Supplementary Material 1

## Data Availability

Phenotypic data used in this study are available in Naik et al. (2024), DOI: 10.1017/S1479262124000042. Genotypic data generated in the current study have been deposited in the Indian Biological Data Centre (IBDC) repository under accession INRP000277: https://ibdc.dbtindia.gov.in/inda/submittedStudyHome.
