# Supplementary material for: Genomic dissection of phenological and yield-associated traits in lentil (Lens culinaris Medik.) using genome-wide association mapping
Source: Physiol Mol Biol Plants. 2026 Apr 7;32(4):839–55. doi: 10.1007/s12298-026-01739-x (PMC13125655; doi:10.1007/s12298-026-01739-x)
Supplement: Supplementary file 1 — Supplementary Material 1 [file 12298_2026_1739_MOESM1_ESM.docx]

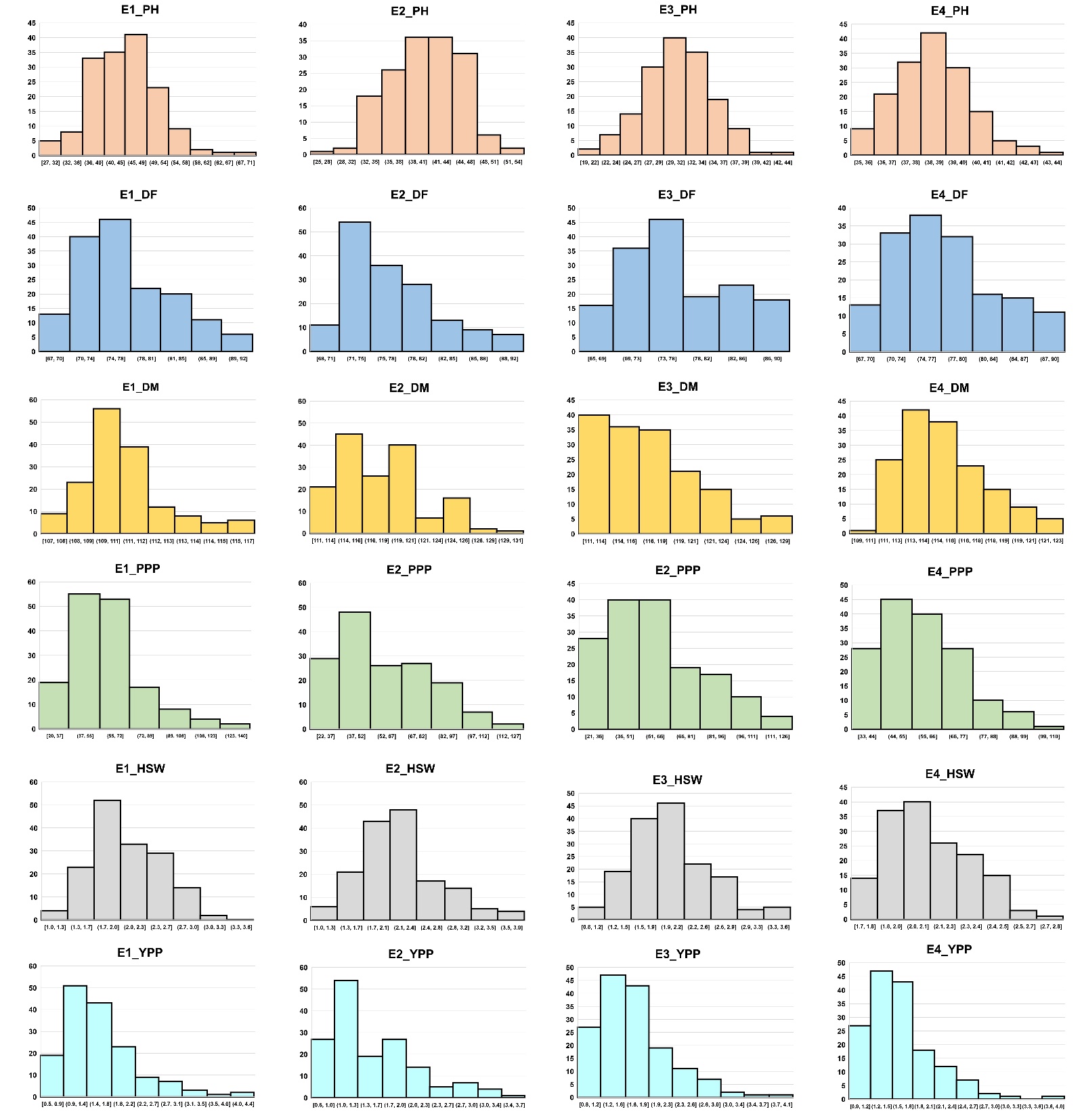


**Supplementary Fig. S1:** Distribution of phenotypic traits across three environments (E1–E3) and combined BLUPs (E4): Plant Height (PH, cm), Days to Flowering (DF), Days to Maturity (DM), Pods per Plant (PPP, g), Hundred Seed Weight (HSW, g), and Yield per Plant (YPP, g). X-axis: Trait values measured for each genotype; Y-axis: Frequency of genotypes corresponding to each trait value.

**Table S1:** Summary of weather data at Pusa and Dholi during the lentil growing seasons (November–March) from 2021 to 2023.

|  | **Dholi 2021-22** | | | | **Pusa and Dholi 2022-23** | | | |
| --- | --- | --- | --- | --- | --- | --- | --- | --- |
| **Month** | **RH2M (%)** | **RZSW (m³/m³)** | **T_MAX (°C)** | **T_MIN (°C)** | **RH2M (%)** | **RZSW (m³/m³)** | **T_MAX (°C)** | **T_MIN (°C)** |
| **Nov** | 79.17 | 0.76 | 26.41 | 14.86 | 75.08 | 0.53 | 30.79 | 18.17 |
| **Nov** | 73.95 | 0.75 | 27.09 | 15.90 | 73.94 | 0.53 | 30.92 | 17.23 |
| **Nov** | 80.53 | 0.74 | 26.25 | 16.07 | 74.21 | 0.52 | 29.89 | 17.00 |
| **Nov** | 84.57 | 0.74 | 26.32 | 14.90 | 67.00 | 0.52 | 30.54 | 17.55 |
| **Nov** | 87.02 | 0.73 | 25.70 | 14.28 | 64.34 | 0.52 | 30.62 | 18.22 |
| **Nov** | 88.09 | 0.73 | 25.05 | 14.89 | 66.91 | 0.51 | 30.57 | 19.21 |
| **Nov** | 87.31 | 0.72 | 24.93 | 14.67 | 70.47 | 0.51 | 30.37 | 17.78 |
| **Nov** | 86.11 | 0.72 | 25.76 | 14.65 | 71.5 | 0.50 | 30.23 | 17.32 |
| **Nov** | 83.00 | 0.71 | 27.11 | 15.69 | 73.54 | 0.50 | 29.6 | 17.3 |
| **Nov** | 82.61 | 0.71 | 27.50 | 16.03 | 74.73 | 0.50 | 28.11 | 17.67 |
| **Nov** | 83.67 | 0.70 | 26.91 | 16.1 | 76.56 | 0.49 | 29.12 | 16.82 |
| **Nov** | 83.89 | 0.70 | 25.54 | 15.57 | 70.88 | 0.49 | 29.16 | 16.26 |
| **Nov** | 85.82 | 0.69 | 25.13 | 14.66 | 66.48 | 0.49 | 28.14 | 12.93 |
| **Nov** | 83.94 | 0.68 | 24.10 | 14.35 | 67.46 | 0.49 | 27.59 | 13.26 |
| **Nov** | 81.68 | 0.68 | 24.15 | 13.27 | 71.68 | 0.48 | 27.87 | 13.50 |
| **Nov** | 85.43 | 0.68 | 25.30 | 13.97 | 73.36 | 0.48 | 28.82 | 14.38 |
| **Nov** | 80.30 | 0.67 | 24.94 | 13.27 | 72.26 | 0.48 | 28.33 | 15.22 |
| **Nov** | 80.74 | 0.67 | 25.75 | 13.62 | 64.13 | 0.48 | 26.74 | 11.86 |
| **Nov** | 85.25 | 0.66 | 25.19 | 13.90 | 64.44 | 0.47 | 27.10 | 12.39 |
| **Nov** | 85.36 | 0.66 | 24.39 | 14.60 | 64.12 | 0.47 | 28.04 | 12.47 |
| **Nov** | 85.62 | 0.65 | 26.32 | 15.61 | 67.53 | 0.47 | 26.99 | 12.31 |
| **Nov** | 82.50 | 0.65 | 25.61 | 14.86 | 73.06 | 0.47 | 27.57 | 12.58 |
| **Nov** | 81.15 | 0.64 | 24.03 | 10.69 | 67.50 | 0.46 | 27.25 | 11.96 |
| **Nov** | 75.26 | 0.64 | 23.40 | 11.20 | 67.77 | 0.46 | 27.07 | 11.26 |
| **Nov** | 74.44 | 0.64 | 24.45 | 12.24 | 68.51 | 0.46 | 27.93 | 11.48 |
| **Nov** | 73.14 | 0.63 | 23.69 | 12.01 | 68.64 | 0.46 | 26.90 | 11.93 |
| **Nov** | 75.30 | 0.63 | 24.33 | 13.33 | 66.85 | 0.46 | 27.01 | 12.61 |
| **Nov** | 82.52 | 0.63 | 24.83 | 14.07 | 64.87 | 0.45 | 27.15 | 13.63 |
| **Nov** | 83.02 | 0.62 | 24.72 | 13.42 | 65.26 | 0.45 | 28.25 | 14.00 |
| **Dec** | 78.20 | 0.62 | 24.84 | 11.28 | 71.03 | 0.45 | 27.89 | 13.18 |
| **Dec** | 74.67 | 0.62 | 25.99 | 14.04 | 68.59 | 0.45 | 26.81 | 11.44 |
| **Dec** | 77.56 | 0.61 | 27.39 | 14.77 | 67.35 | 0.45 | 25.43 | 10.33 |
| **Dec** | 82.09 | 0.61 | 26.47 | 14.56 | 72.82 | 0.45 | 24.82 | 11.37 |
| **Dec** | 79.69 | 0.60 | 27.18 | 15.15 | 74.11 | 0.44 | 26.14 | 10.82 |
| **Dec** | 83.46 | 0.60 | 26.82 | 17.03 | 73.83 | 0.44 | 25.67 | 11.87 |
| **Dec** | 80.77 | 0.60 | 26.69 | 18.33 | 71.83 | 0.44 | 25.09 | 10.8 |
| **Dec** | 80.56 | 0.59 | 26.98 | 16.12 | 70.79 | 0.44 | 24.80 | 9.97 |
| **Dec** | 74.41 | 0.59 | 25.78 | 11.96 | 67.15 | 0.44 | 24.72 | 9.85 |
| **Dec** | 72.68 | 0.59 | 25.58 | 12.25 | 65.2 | 0.44 | 26.01 | 11.19 |
| **Dec** | 74.64 | 0.58 | 24.12 | 11.66 | 69.14 | 0.43 | 26.85 | 12.00 |
| **Dec** | 73.11 | 0.58 | 22.69 | 9.27 | 75.69 | 0.43 | 25.6 | 10.94 |
| **Dec** | 73.78 | 0.58 | 23.06 | 10.84 | 69.92 | 0.43 | 26.6 | 10.94 |
| **Dec** | 72.93 | 0.57 | 22.40 | 9.37 | 64.94 | 0.43 | 27.74 | 12.05 |
| **Dec** | 78.96 | 0.57 | 22.31 | 9.13 | 65.68 | 0.43 | 26.16 | 9.76 |
| **Dec** | 80.57 | 0.57 | 22.88 | 8.99 | 65.35 | 0.43 | 25.10 | 9.30 |
| **Dec** | 75.35 | 0.56 | 24.31 | 9.35 | 65.25 | 0.43 | 23.27 | 7.65 |
| **Dec** | 77.7 | 0.56 | 22.97 | 9.40 | 68.32 | 0.42 | 23.78 | 8.96 |
| **Dec** | 71.13 | 0.56 | 23.09 | 9.36 | 67.2 | 0.42 | 24.55 | 10.64 |
| **Dec** | 73.49 | 0.55 | 18.81 | 4.82 | 67.87 | 0.42 | 25.00 | 11.34 |
| **Dec** | 78.26 | 0.55 | 19.64 | 5.34 | 69.05 | 0.42 | 25.38 | 9.63 |
| **Dec** | 74.10 | 0.55 | 19.39 | 7.10 | 69.07 | 0.42 | 24.82 | 10.33 |
| **Dec** | 74.32 | 0.55 | 21.34 | 6.47 | 61.96 | 0.42 | 25.11 | 8.82 |
| **Dec** | 72.65 | 0.55 | 21.93 | 7.43 | 59.06 | 0.42 | 26.58 | 9.55 |
| **Dec** | 68.07 | 0.54 | 22.48 | 10.12 | 57.74 | 0.42 | 24.74 | 11.93 |
| **Dec** | 82.75 | 0.54 | 21.97 | 9.78 | 60.57 | 0.41 | 27.50 | 15.29 |
| **Dec** | 83.84 | 0.54 | 22.79 | 10.94 | 64.53 | 0.41 | 26.50 | 14.56 |
| **Dec** | 83.13 | 0.54 | 22.43 | 13.15 | 51.9 | 0.41 | 23.75 | 10.8 |
| **Dec** | 85.77 | 0.54 | 23.03 | 12.49 | 58.67 | 0.41 | 22.13 | 7.70 |
| **Dec** | 89.27 | 0.54 | 18.33 | 14.03 | 50.95 | 0.41 | 22.81 | 8.46 |
| **Dec** | 81.32 | 0.54 | 22.13 | 13.33 | 49.79 | 0.41 | 22.86 | 10.02 |
| **Dec** | 80.51 | 0.54 | 22.01 | 10.08 | 55.97 | 0.41 | 23.63 | 11.41 |
| **Jan** | 78.37 | 0.54 | 22.83 | 11.28 | 60.93 | 0.41 | 23.13 | 11.24 |
| **Jan** | 76.50 | 0.54 | 21.95 | 8.05 | 65.32 | 0.40 | 22.93 | 10.33 |
| **Jan** | 75.07 | 0.53 | 21.47 | 8.00 | 68.33 | 0.40 | 22.33 | 8.61 |
| **Jan** | 74.85 | 0.53 | 21.57 | 7.34 | 67.52 | 0.40 | 21.61 | 7.53 |
| **Jan** | 73.38 | 0.53 | 22.44 | 8.72 | 66.39 | 0.40 | 21.83 | 6.41 |
| **Jan** | 72.97 | 0.53 | 24.35 | 11.32 | 63.32 | 0.40 | 22.61 | 5.69 |
| **Jan** | 82.28 | 0.52 | 23.81 | 12.20 | 62.49 | 0.40 | 21.81 | 4.85 |
| **Jan** | 81.14 | 0.52 | 25.41 | 11.78 | 59.37 | 0.40 | 22.38 | 5.15 |
| **Jan** | 79.39 | 0.52 | 26.17 | 12.32 | 56.24 | 0.40 | 22.66 | 5.96 |
| **Jan** | 78.25 | 0.52 | 25.35 | 14.27 | 58.96 | 0.40 | 23.79 | 6.95 |
| **Jan** | 83.47 | 0.52 | 23.44 | 14.27 | 57.28 | 0.39 | 23.05 | 6.74 |
| **Jan** | 84.85 | 0.52 | 22.44 | 11.53 | 54.65 | 0.39 | 24.5 | 7.83 |
| **Jan** | 75.00 | 0.52 | 22.07 | 10.00 | 53.07 | 0.39 | 26.15 | 9.85 |
| **Jan** | 71.98 | 0.52 | 21.6 | 8.41 | 55.31 | 0.39 | 26.84 | 12.74 |
| **Jan** | 72.50 | 0.51 | 22.26 | 8.03 | 62.62 | 0.39 | 23.13 | 10.58 |
| **Jan** | 75.20 | 0.51 | 20.95 | 7.98 | 45.16 | 0.39 | 21.43 | 6.25 |
| **Jan** | 77.46 | 0.51 | 20.00 | 7.62 | 48.57 | 0.39 | 21.39 | 4.92 |
| **Jan** | 72.51 | 0.51 | 22.58 | 7.51 | 54.3 | 0.38 | 21.59 | 5.69 |
| **Jan** | 71.62 | 0.50 | 22.14 | 7.82 | 49.54 | 0.38 | 22.49 | 7.14 |
| **Jan** | 73.39 | 0.50 | 21.6 | 8.36 | 40.03 | 0.38 | 24.77 | 7.35 |
| **Jan** | 68.64 | 0.50 | 23.47 | 9.43 | 49.61 | 0.38 | 24.47 | 12.39 |
| **Jan** | 70.10 | 0.50 | 24.12 | 10.27 | 60.81 | 0.38 | 27.57 | 12.48 |
| **Jan** | 86.49 | 0.50 | 19.50 | 12.90 | 53.30 | 0.38 | 28.16 | 11.66 |
| **Jan** | 74.37 | 0.50 | 23.73 | 12.16 | 52.85 | 0.38 | 28.13 | 14.74 |
| **Jan** | 70.85 | 0.49 | 22.60 | 11.49 | 54.91 | 0.38 | 29.54 | 14.62 |
| **Jan** | 66.34 | 0.49 | 21.32 | 8.93 | 58.50 | 0.38 | 30.53 | 14.94 |
| **Jan** | 67.54 | 0.49 | 20.54 | 7.12 | 64.22 | 0.37 | 29.26 | 15.23 |
| **Jan** | 68.51 | 0.49 | 20.76 | 8.41 | 51.21 | 0.37 | 27.02 | 12.76 |
| **Jan** | 67.95 | 0.48 | 20.48 | 5.64 | 46.79 | 0.37 | 27.61 | 11.03 |
| **Jan** | 62.79 | 0.48 | 22.76 | 6.23 | 44.21 | 0.37 | 29.12 | 13.87 |
| **Feb** | 62.09 | 0.48 | 23.61 | 7.96 | 48.51 | 0.37 | 30.00 | 12.8 |
| **Feb** | 69.18 | 0.48 | 23.41 | 8.10 | 54.62 | 0.37 | 26.25 | 12.88 |
| **Feb** | 64.29 | 0.47 | 24.92 | 8.22 | 48.83 | 0.36 | 26.47 | 9.96 |
| **Feb** | 66.37 | 0.48 | 24.33 | 8.85 | 48.74 | 0.36 | 26.28 | 9.47 |
| **Feb** | 84.61 | 0.53 | 21.53 | 10.49 | 49.22 | 0.36 | 27.29 | 9.33 |
| **Feb** | 73.35 | 0.54 | 20.96 | 8.66 | 47.22 | 0.36 | 27.86 | 10.09 |
| **Feb** | 71.35 | 0.54 | 20.71 | 6.13 | 45.66 | 0.36 | 28.38 | 11.71 |
| **Feb** | 69.67 | 0.53 | 21.93 | 7.17 | 44.34 | 0.36 | 30.07 | 11.4 |
| **Feb** | 66.68 | 0.53 | 23.18 | 7.53 | 46.75 | 0.36 | 29.29 | 13.32 |
| **Feb** | 58.80 | 0.53 | 25.57 | 9.86 | 42.90 | 0.35 | 28.38 | 10.82 |
| **Feb** | 66.43 | 0.52 | 25.37 | 13.13 | 37.67 | 0.35 | 28.95 | 11.42 |
| **Feb** | 64.66 | 0.52 | 23.67 | 10.34 | 37.23 | 0.35 | 31.43 | 12.87 |
| **Feb** | 63.48 | 0.51 | 23.66 | 7.57 | 36.84 | 0.35 | 30.14 | 13.48 |
| **Feb** | 59.69 | 0.51 | 24.56 | 9.75 | 30.01 | 0.35 | 27.91 | 9.18 |
| **Feb** | 56.06 | 0.51 | 24.68 | 10.28 | 31.17 | 0.35 | 27.36 | 10.67 |
| **Feb** | 61.19 | 0.50 | 25.86 | 9.46 | 30.38 | 0.35 | 26.92 | 11.83 |
| **Feb** | 63.91 | 0.50 | 26.36 | 9.62 | 33.27 | 0.34 | 29.75 | 13.1 |
| **Feb** | 59.76 | 0.50 | 26.42 | 9.74 | 37.18 | 0.34 | 30.26 | 15.83 |
| **Feb** | 57.13 | 0.49 | 26.83 | 8.89 | 38.88 | 0.34 | 30.93 | 16.26 |
| **Feb** | 57.60 | 0.49 | 27.81 | 9.20 | 39.24 | 0.34 | 32.03 | 17.25 |
| **Feb** | 56.41 | 0.48 | 27.55 | 10.54 | 43.75 | 0.34 | 32.01 | 16.54 |
| **Feb** | 53.49 | 0.48 | 27.57 | 11.51 | 39.15 | 0.34 | 33.61 | 16.86 |
| **Feb** | 46.39 | 0.48 | 26.96 | 12.46 | 33.81 | 0.34 | 34.46 | 17.27 |
| **Feb** | 56.33 | 0.47 | 27.76 | 10.06 | 30.34 | 0.34 | 34.08 | 15.11 |
| **Feb** | 60.55 | 0.47 | 29.33 | 12.91 | 28.08 | 0.34 | 33.45 | 13.92 |
| **Feb** | 66.15 | 0.47 | 27.83 | 16.64 | 24.96 | 0.33 | 32.03 | 15.40 |
| **Feb** | 63.58 | 0.47 | 29.14 | 13.84 | 31.01 | 0.33 | 31.92 | 13.26 |
| **Feb** | 50.8 | 0.46 | 30.73 | 15.85 | 29.85 | 0.33 | 32.09 | 17.09 |
| **Mar** | 50.98 | 0.46 | 29.85 | 14.02 | 31.77 | 0.33 | 33.41 | 17.32 |
| **Mar** | 45.14 | 0.46 | 30.29 | 12.93 | 32.48 | 0.33 | 33.87 | 16.09 |
| **Mar** | 50.92 | 0.45 | 30.23 | 12.69 | 30.1 | 0.33 | 34.58 | 17.33 |
| **Mar** | 48.06 | 0.45 | 30.78 | 12.66 | 33.27 | 0.33 | 35.34 | 16.22 |
| **Mar** | 45.6 | 0.45 | 32.47 | 13.25 | 32.85 | 0.33 | 35.39 | 17.24 |
| **Mar** | 49.92 | 0.44 | 31.57 | 13.86 | 32.31 | 0.33 | 35.18 | 16.52 |
| **Mar** | 47.31 | 0.44 | 31.31 | 13.35 | 30.77 | 0.33 | 35.03 | 16.77 |
| **Mar** | 46.35 | 0.43 | 33.26 | 15.22 | 28.22 | 0.33 | 35.27 | 16.95 |
| **Mar** | 43.50 | 0.43 | 33.76 | 14.89 | 32.67 | 0.33 | 33.08 | 19.15 |
| **Mar** | 45.38 | 0.43 | 33.26 | 14.75 | 34.45 | 0.33 | 34.23 | 17.53 |
| **Mar** | 43.00 | 0.42 | 34.57 | 15.43 | 30.83 | 0.33 | 34.68 | 17.19 |
| **Mar** | 45.48 | 0.42 | 34.16 | 16.17 | 29.92 | 0.33 | 34.98 | 19.39 |
| **Mar** | 43.82 | 0.42 | 33.89 | 15.42 | 30.82 | 0.33 | 35.17 | 18.88 |
| **Mar** | 42.15 | 0.41 | 34.25 | 16.64 | 33.64 | 0.33 | 35.79 | 19.94 |
| **Mar** | 42.25 | 0.41 | 35.55 | 16.91 | 34.23 | 0.33 | 34.4 | 19.84 |
| **Mar** | 41.90 | 0.41 | 36.06 | 19.22 | 31.08 | 0.33 | 36.29 | 20.26 |
| **Mar** | 36.53 | 0.40 | 36.78 | 20.87 | 36.88 | 0.33 | 33.01 | 20.18 |
| **Mar** | 37.14 | 0.40 | 37.71 | 20.21 | 47.34 | 0.33 | 33.00 | 19.61 |
| **Mar** | 44.50 | 0.40 | 38.62 | 21.50 | 46.50 | 0.34 | 32.18 | 21.09 |
| **Mar** | 42.00 | 0.39 | 39.30 | 22.04 | 60.14 | 0.35 | 29.53 | 20.13 |
| **Mar** | 42.80 | 0.39 | 38.84 | 23.13 | 81.25 | 0.36 | 24.71 | 19.17 |
| **Mar** | 40.43 | 0.39 | 38.34 | 23.35 | 66.72 | 0.36 | 28.61 | 16.84 |
| **Mar** | 35.42 | 0.39 | 38.79 | 21.19 | 50.93 | 0.36 | 31.98 | 17.24 |
| **Mar** | 34.64 | 0.38 | 37.97 | 21.23 | 45.96 | 0.36 | 32.90 | 16.35 |
| **Mar** | 32.83 | 0.38 | 38.01 | 19.70 | 40.66 | 0.36 | 34.98 | 17.77 |
| **Mar** | 34.34 | 0.38 | 39.13 | 20.49 | 37.29 | 0.35 | 36.15 | 20.31 |
| **Mar** | 28.64 | 0.37 | 38.99 | 19.66 | 37.97 | 0.35 | 36.98 | 20.75 |
| **Mar** | 31.49 | 0.37 | 37.98 | 18.19 | 37.15 | 0.35 | 36.26 | 19.62 |
| **Mar** | 28.77 | 0.36 | 37.87 | 19.20 | 33.06 | 0.34 | 36.44 | 19.67 |
| **Mar** | 37.91 | 0.36 | 37.51 | 19.54 | 31.44 | 0.34 | 36.58 | 19.02 |
| **Mar** | 44.23 | 0.36 | 37.20 | 20.97 | 27.84 | 0.34 | 36.94 | 20.58 |
| **Mar** | 48.98 | 0.36 | 37.63 | 21.84 | 37.00 | 0.34 | 31.56 | 20.09 |

**Note:** RH2M – Relative Humidity; RZSW – Root Zone Soil Wetness; T_MAX – Maximum Temperature; T_MIN – Minimum Temperature.

| **Table S2:** Comparative analysis of soil physico-chemical properties at Pusa and Dholi locations. | | |
| --- | --- | --- |
| **Soil Properties** | **Pusa** | **Dholi** |
| Textural class | Sandy loam | Sandy loam |
| Sand (%) | 58.22 | 55.74 |
| Silt (%) | 28.61 | 29.83 |
| Clay (%) | 12.83 | 14.43 |
| Bulk Density (Mg m⁻³) | 1.52 | 1.31 |
| Soil pH (1:2.5) | 8.83 | 8.21 |
| Electrical Conductivity (dS m⁻¹) | 0.54 | 0.31 |
| Organic Carbon (%) | 0.60 | 0.40 |
| Available Nitrogen (kg ha⁻¹) | 116.00 | 172.60 |
| Available Phosphorus (P₂O₅, kg ha⁻¹) | 15.00 | 17.50 |
| Available Potassium (K₂O, kg ha⁻¹) | 77.00 | 121.3 |
| Available Sulphur (mg kg⁻¹) | 6.79 | 25.36 |

**Table S3:** Minimum, maximum, and mean values of key agronomic traits across three environments (E1–E3) and combined BLUPs in lentil.

|  | **E1 (Dholi, 2020–2021)** | | | **E2 (Dholi, 2021–2022)** | | | **E3 (Pusa, 2021-2022)** | | | **BLUPs** | | |
| --- | --- | --- | --- | --- | --- | --- | --- | --- | --- | --- | --- | --- |
| **Trait** | **MIN** | **MAX** | **AVG** | **MIN** | **MAX** | **AVG** | **MIN** | **MAX** | **AVG** | **MIN** | **MAX** | **AVG** |
| **PH** | 24.98 | 59.80 | 42.33 | 22.80 | 51.80 | 40.53 | 19.00 | 43.25 | 30.86 | 35.01 | 43.44 | 38.48 |
| **DF** | 66.00 | 90.00 | 76.89 | 68.00 | 91.00 | 77.22 | 65.00 | 90.00 | 76.53 | 67.06 | 90.12 | 77.12 |
| **DM** | 99.00 | 121.00 | 110.75 | 101.00 | 129.00 | 117.67 | 101.00 | 128.00 | 117.07 | 109.47 | 122.62 | 115.25 |
| **PPP** | 20.41 | 140.00 | 58.11 | 22.00 | 115.00 | 57.42 | 21.00 | 125.00 | 58.55 | 32.81 | 109.96 | 58.28 |
| **HSW** | 1.01 | 3.64 | 2.08 | 0.97 | 3.91 | 2.21 | 0.80 | 3.55 | 2.02 | 1.69 | 2.68 | 2.11 |
| **YPP** | 0.51 | 4.12 | 1.58 | 0.61 | 3.74 | 1.53 | 0.84 | 4.07 | 1.49 | 0.86 | 3.67 | 1.60 |

**Note:** Average (AVG); days to 50% flowering (DF); days to maturity (DM); environment 1 (E1); environment 2 (E2); environment 3 (E3); environment 4 (E4); grain yield per plant (YPP); hundred seed weight (HSW); Maximum (MAX); Minimum (MIN); number of pods per plant (PPP); plant height (PH).

| **Table S4: Summary of sequencing data statistics and quality assessment parameters** | | | | | | | | | | |
| --- | --- | --- | --- | --- | --- | --- | --- | --- | --- | --- |
| **Genotype** | **Paired Read** | **Bases (Gb)** | **Reads (millions)** | **% bases >=Q20** | **% bases >=Q30** | **Average**  **Read length** | **Read length range (bp)** | **Qual range** | **Phred range** | **GC**  **content** |
| 10-3-1-26 | R2 | 5.53 | 40.35 | 97.38 | 93.01 | 131 | 131-131 | 35-70 | 2-37 | 37.58 |
| 10-3-1-26 | R1 |  |  | 96.61 | 91.45 | 143 | 143-143 | 35-70 | 2-37 | 37.99 |
| DPL15 | R2 | 5.30 | 38.69 | 97.45 | 93.14 | 131 | 131-131 | 35-70 | 2-37 | 37.53 |
| DPL15 | R1 |  |  | 96.65 | 91.52 | 143 | 143-143 | 35-70 | 2-37 | 38.00 |
| EC139824-A | R2 | 1.80 | 13.10 | 97.24 | 92.85 | 131 | 131-131 | 35-70 | 2-37 | 37.50 |
| EC139824-A | R1 |  |  | 96.51 | 91.34 | 143 | 143-143 | 35-70 | 2-37 | 37.98 |
| EC223150 | R2 | 3.40 | 24.82 | 97.37 | 92.99 | 131 | 131-131 | 35-70 | 2-37 | 37.95 |
| EC223150 | R1 |  |  | 96.62 | 91.54 | 143 | 143-143 | 35-70 | 2-37 | 38.28 |
| EC223188 | R2 | 0.02 | 0.16 | 93.85 | 86.35 | 131 | 131-131 | 35-70 | 2-37 | 39.00 |
| EC223188 | R1 |  |  | 94.64 | 87.60 | 143 | 143-143 | 35-70 | 2-37 | 39.27 |
| EC223191 | R2 | 5.62 | 41.01 | 97.32 | 92.86 | 131 | 131-131 | 35-70 | 2-37 | 37.35 |
| EC223191 | R1 |  |  | 96.68 | 91.58 | 143 | 143-143 | 35-70 | 2-37 | 37.90 |
| EC223197-A | R2 | 3.38 | 24.69 | 97.36 | 92.98 | 131 | 131-131 | 35-70 | 2-37 | 37.51 |
| EC223197-A | R1 |  |  | 96.58 | 91.39 | 143 | 143-143 | 35-70 | 2-37 | 38.05 |
| EC223197-B | R2 | 2.72 | 19.84 | 97.35 | 93.01 | 131 | 131-131 | 35-70 | 2-37 | 37.63 |
| EC223197-B | R1 |  |  | 96.52 | 91.24 | 143 | 143-143 | 35-70 | 2-37 | 38.17 |
| EC223199-B | R2 | 3.95 | 28.84 | 97.33 | 92.92 | 131 | 131-131 | 35-70 | 2-37 | 37.53 |
| EC223199-B | R1 |  |  | 96.66 | 91.52 | 143 | 143-143 | 35-70 | 2-37 | 38.00 |
| EC223201 | R2 | 5.69 | 41.51 | 97.48 | 93.17 | 131 | 131-131 | 35-70 | 2-37 | 37.61 |
| EC223201 | R1 |  |  | 96.61 | 91.47 | 143 | 143-143 | 35-70 | 2-37 | 38.05 |
| EC223205-B | R2 | 3.08 | 22.50 | 97.40 | 93.06 | 131 | 131-131 | 35-70 | 2-37 | 37.69 |
| EC223205-B | R1 |  |  | 96.69 | 91.63 | 143 | 143-143 | 35-70 | 2-37 | 38.14 |
| EC223207 | R2 | 4.87 | 35.56 | 97.51 | 93.21 | 131 | 131-131 | 35-70 | 2-37 | 37.77 |
| EC223207 | R1 |  |  | 96.77 | 91.87 | 143 | 143-143 | 35-70 | 2-37 | 38.09 |
| EC223209-B | R2 | 2.78 | 20.28 | 97.33 | 92.9 | 131 | 131-131 | 35-70 | 2-37 | 37.70 |
| EC223209-B | R1 |  |  | 96.53 | 91.34 | 143 | 143-143 | 35-70 | 2-37 | 38.28 |
| EC223212-A | R2 | 4.01 | 29.28 | 97.44 | 93.16 | 131 | 131-131 | 35-70 | 2-37 | 37.51 |
| EC223212-A | R1 |  |  | 96.61 | 91.46 | 143 | 143-143 | 35-70 | 2-37 | 38.08 |
| EC223215 | R2 | 0.39 | 2.86 | 97.44 | 93.11 | 131 | 131-131 | 35-70 | 2-37 | 37.05 |
| EC223215 | R1 |  |  | 96.68 | 91.57 | 143 | 143-143 | 35-70 | 2-37 | 37.62 |
| EC223220 | R2 | 5.18 | 37.83 | 97.35 | 92.96 | 131 | 131-131 | 35-70 | 2-37 | 37.37 |
| EC223220 | R1 |  |  | 96.64 | 91.47 | 143 | 143-143 | 35-70 | 2-37 | 37.92 |
| EC223221 | R2 | 3.44 | 25.14 | 97.44 | 93.16 | 131 | 131-131 | 35-70 | 2-37 | 37.82 |
| EC223221 | R1 |  |  | 96.66 | 91.61 | 143 | 143-143 | 35-70 | 2-37 | 38.17 |
| EC223222 | R2 | 8.20 | 59.84 | 97.46 | 93.01 | 131 | 131-131 | 35-70 | 2-37 | 37.12 |
| EC223222 | R1 |  |  | 95.73 | 89.72 | 143 | 143-143 | 35-70 | 2-37 | 37.84 |
| EC223223 | R2 | 6.54 | 47.76 | 97.58 | 93.35 | 131 | 131-131 | 35-70 | 2-37 | 37.46 |
| EC223223 | R1 |  |  | 96.69 | 91.61 | 143 | 143-143 | 35-70 | 2-37 | 37.86 |
| EC223226 | R2 | 3.36 | 24.54 | 97.50 | 93.18 | 131 | 131-131 | 35-70 | 2-37 | 37.88 |
| EC223226 | R1 |  |  | 96.74 | 91.76 | 143 | 143-143 | 35-70 | 2-37 | 38.14 |
| EC223229-A | R2 | 3.23 | 23.56 | 97.48 | 93.12 | 131 | 131-131 | 35-70 | 2-37 | 37.90 |
| EC223229-A | R1 |  |  | 96.94 | 92.18 | 143 | 143-143 | 35-70 | 2-37 | 38.18 |
| EC223229-B | R2 | 5.14 | 37.50 | 97.42 | 93.19 | 131 | 131-131 | 35-70 | 2-37 | 37.38 |
| EC223229-B | R1 |  |  | 96.53 | 91.26 | 143 | 143-143 | 35-70 | 2-37 | 37.85 |
| EC223230 | R2 | 5.00 | 36.49 | 97.35 | 93.03 | 131 | 131-131 | 35-70 | 2-37 | 37.20 |
| EC223230 | R1 |  |  | 96.48 | 91.14 | 143 | 143-143 | 35-70 | 2-37 | 37.85 |
| EC223242 | R2 | 0.07 | 0.49 | 96.55 | 91.23 | 131 | 131-131 | 35-70 | 2-37 | 37.13 |
| EC223242 | R1 |  |  | 95.67 | 89.70 | 143 | 143-143 | 35-70 | 2-37 | 37.90 |
| EC223397 | R2 | 9.33 | 68.10 | 97.43 | 92.94 | 131 | 131-131 | 35-70 | 2-37 | 37.05 |
| EC223397 | R1 |  |  | 95.82 | 89.91 | 143 | 143-143 | 35-70 | 2-37 | 37.81 |
| EC225501 | R2 | 0.06 | 0.42 | 94.97 | 88.88 | 131 | 131-131 | 35-70 | 2-37 | 36.97 |
| EC225501 | R1 |  |  | 93.88 | 86.80 | 143 | 143-143 | 35-70 | 2-37 | 37.98 |
| EC255491 | R2 | 0.02 | 0.16 | 93.89 | 86.54 | 131 | 131-131 | 35-70 | 2-37 | 38.32 |
| EC255491 | R1 |  |  | 93.68 | 86.29 | 143 | 143-143 | 35-70 | 2-37 | 39.14 |
| EC267529 | R2 | 9.06 | 66.15 | 97.29 | 92.74 | 131 | 131-131 | 35-70 | 2-37 | 37.65 |
| EC267529 | R1 |  |  | 95.77 | 89.85 | 143 | 143-143 | 35-70 | 2-37 | 38.16 |
| EC267533 | R2 | 7.78 | 56.75 | 97.47 | 93.10 | 131 | 131-131 | 35-70 | 2-37 | 37.85 |
| EC267533 | R1 |  |  | 95.80 | 89.92 | 143 | 143-143 | 35-70 | 2-37 | 38.20 |
| EC267536 | R2 | 8.37 | 61.06 | 97.41 | 92.99 | 131 | 131-131 | 35-70 | 2-37 | 37.00 |
| EC267536 | R1 |  |  | 95.67 | 89.53 | 143 | 143-143 | 35-70 | 2-37 | 37.87 |
| EC267539 | R2 | 10.72 | 78.21 | 97.46 | 93.01 | 131 | 131-131 | 35-70 | 2-37 | 37.38 |
| EC267539 | R1 |  |  | 95.64 | 89.60 | 143 | 143-143 | 35-70 | 2-37 | 38.05 |
| EC267540 | R2 | 0.03 | 0.24 | 93.24 | 85.36 | 131 | 131-131 | 35-70 | 2-37 | 38.18 |
| EC267540 | R1 |  |  | 93.86 | 86.55 | 143 | 143-143 | 35-70 | 2-37 | 38.81 |
| EC267544-A | R2 | 8.38 | 61.17 | 97.30 | 92.79 | 131 | 131-131 | 35-70 | 2-37 | 36.90 |
| EC267544-A | R1 |  |  | 95.54 | 89.30 | 143 | 143-143 | 35-70 | 2-37 | 37.86 |
| EC267545-D | R2 | 0.02 | 0.14 | 94.55 | 87.48 | 131 | 131-131 | 35-70 | 2-37 | 38.34 |
| EC267545-D | R1 |  |  | 95.20 | 88.61 | 143 | 143-143 | 35-70 | 2-37 | 38.78 |
| EC2675471 | R2 | 6.20 | 45.27 | 97.44 | 93.02 | 131 | 131-131 | 35-70 | 2-37 | 37.47 |
| EC2675471 | R1 |  |  | 95.69 | 89.68 | 143 | 143-143 | 35-70 | 2-37 | 38.07 |
| EC267554 | R2 | 5.25 | 38.33 | 97.46 | 93.06 | 131 | 131-131 | 35-70 | 2-37 | 37.78 |
| EC267554 | R1 |  |  | 95.70 | 89.72 | 143 | 143-143 | 35-70 | 2-37 | 38.29 |
| EC267557-D | R2 | 7.68 | 56.07 | 96.89 | 91.89 | 131 | 131-131 | 35-70 | 2-37 | 37.35 |
| EC267557-D | R1 |  |  | 96.04 | 90.40 | 143 | 143-143 | 35-70 | 2-37 | 37.95 |
| EC267563 | R2 | 9.33 | 68.13 | 97.55 | 93.15 | 131 | 131-131 | 35-70 | 2-37 | 37.48 |
| EC267563 | R1 |  |  | 96.03 | 90.33 | 143 | 143-143 | 35-70 | 2-37 | 37.88 |
| EC267567 | R2 | 0.00 | 0.03 | 91.21 | 82.20 | 131 | 131-131 | 35-70 | 2-37 | 38.93 |
| EC267567 | R1 |  |  | 87.88 | 78.72 | 143 | 143-143 | 35-70 | 2-37 | 42.13 |
| EC267569-A | R2 | 6.97 | 50.87 | 97.41 | 92.92 | 131 | 131-131 | 35-70 | 2-37 | 37.42 |
| EC267569-A | R1 |  |  | 95.93 | 90.13 | 143 | 143-143 | 35-70 | 2-37 | 37.92 |
| EC267569-B | R2 | 5.44 | 39.69 | 97.41 | 92.9 | 131 | 131-131 | 35-70 | 2-37 | 37.54 |
| EC267569-B | R1 |  |  | 95.83 | 89.9 | 143 | 143-143 | 35-70 | 2-37 | 38.17 |
| EC267573 | R2 | 0.03 | 0.23 | 94.68 | 88.31 | 131 | 131-131 | 35-70 | 2-37 | 37.62 |
| EC267573 | R1 |  |  | 94.14 | 87.13 | 143 | 143-143 | 35-70 | 2-37 | 38.39 |
| EC2675770 | R2 | 0.03 | 0.21 | 94.08 | 87.14 | 131 | 131-131 | 35-70 | 2-37 | 37.87 |
| EC2675770 | R1 |  |  | 94.23 | 87.06 | 143 | 143-143 | 35-70 | 2-37 | 38.49 |
| EC267595-C | R2 | 0.03 | 0.20 | 94.32 | 87.01 | 131 | 131-131 | 35-70 | 2-37 | 38.92 |
| EC267595-C | R1 |  |  | 95.41 | 88.99 | 143 | 143-143 | 35-70 | 2-37 | 39.21 |
| EC267604 | R2 | 0.02 | 0.17 | 94.53 | 87.60 | 131 | 131-131 | 35-70 | 2-37 | 38.31 |
| EC267604 | R1 |  |  | 94.87 | 88.04 | 143 | 143-143 | 35-70 | 2-37 | 38.66 |
| EC267605 | R2 | 0.04 | 0.32 | 95.10 | 88.75 | 131 | 131-131 | 35-70 | 2-37 | 37.38 |
| EC267605 | R1 |  |  | 94.88 | 88.26 | 143 | 143-143 | 35-70 | 2-37 | 38.03 |
| EC267609 | R2 | 0.05 | 0.35 | 95.02 | 88.65 | 131 | 131-131 | 35-70 | 2-37 | 37.61 |
| EC267609 | R1 |  |  | 94.50 | 87.64 | 143 | 143-143 | 35-70 | 2-37 | 38.44 |
| EC267625-C | R2 | 0.03 | 0.24 | 94.93 | 88.25 | 131 | 131-131 | 35-70 | 2-37 | 38.01 |
| EC267625-C | R1 |  |  | 95.05 | 88.27 | 143 | 143-143 | 35-70 | 2-37 | 38.56 |
| EC267628-A | R2 | 6.92 | 50.48 | 97.39 | 92.92 | 131 | 131-131 | 35-70 | 2-37 | 36.93 |
| EC267628-A | R1 |  |  | 95.66 | 89.59 | 143 | 143-143 | 35-70 | 2-37 | 37.78 |
| EC267634 | R2 | 3.54 | 25.84 | 97.26 | 92.83 | 131 | 131-131 | 35-70 | 2-37 | 37.44 |
| EC267634 | R1 |  |  | 96.45 | 91.10 | 143 | 143-143 | 35-70 | 2-37 | 38.12 |
| EC267636 | R2 | 5.30 | 38.69 | 97.36 | 92.87 | 131 | 131-131 | 35-70 | 2-37 | 37.40 |
| EC267636 | R1 |  |  | 95.64 | 89.63 | 143 | 143-143 | 35-70 | 2-37 | 38.08 |
| EC267638 | R2 | 11.07 | 80.78 | 97.51 | 93.10 | 131 | 131-131 | 35-70 | 2-37 | 37.16 |
| EC267638 | R1 |  |  | 95.76 | 89.78 | 143 | 143-143 | 35-70 | 2-37 | 37.89 |
| EC267641 | R2 | 6.65 | 48.58 | 97.38 | 92.91 | 131 | 131-131 | 35-70 | 2-37 | 36.92 |
| EC267641 | R1 |  |  | 95.70 | 89.62 | 143 | 143-143 | 35-70 | 2-37 | 37.76 |
| EC267676 | R2 | 6.33 | 46.22 | 97.65 | 93.38 | 131 | 131-131 | 35-70 | 2-37 | 37.87 |
| EC267676 | R1 |  |  | 96.13 | 90.51 | 143 | 143-143 | 35-70 | 2-37 | 38.08 |
| EC267696 | R2 | 4.19 | 30.60 | 97.28 | 92.84 | 131 | 131-131 | 35-70 | 2-37 | 37.42 |
| EC267696 | R1 |  |  | 96.6 | 91.64 | 143 | 143-143 | 35-70 | 2-37 | 37.89 |
| EC267709 | R2 | 4.79 | 34.99 | 97.30 | 92.87 | 131 | 131-131 | 35-70 | 2-37 | 36.98 |
| EC267709 | R1 |  |  | 96.50 | 91.18 | 143 | 143-143 | 35-70 | 2-37 | 37.70 |
| EC329166 | R2 | 3.91 | 28.54 | 97.40 | 93.00 | 131 | 131-131 | 35-70 | 2-37 | 37.86 |
| EC329166 | R1 |  |  | 96.83 | 92.00 | 143 | 143-143 | 35-70 | 2-37 | 38.13 |
| EC78395 | R2 | 3.59 | 26.23 | 97.25 | 92.78 | 131 | 131-131 | 35-70 | 2-37 | 37.29 |
| EC78395 | R1 |  |  | 96.46 | 91.11 | 143 | 143-143 | 35-70 | 2-37 | 37.91 |
| EC78408 | R2 | 1.16 | 8.44 | 97.26 | 92.78 | 131 | 131-131 | 35-70 | 2-37 | 36.91 |
| EC78408 | R1 |  |  | 96.77 | 91.78 | 143 | 143-143 | 35-70 | 2-37 | 37.63 |
| EC78414 | R2 | 4.63 | 33.81 | 97.47 | 93.23 | 131 | 131-131 | 35-70 | 2-37 | 37.84 |
| EC78414 | R1 |  |  | 96.79 | 91.86 | 143 | 143-143 | 35-70 | 2-37 | 38.04 |
| EC78425 | R2 | 0.02 | 0.15 | 94.08 | 86.76 | 131 | 131-131 | 35-70 | 2-37 | 38.15 |
| EC78425 | R1 |  |  | 94.55 | 87.41 | 143 | 143-143 | 35-70 | 2-37 | 38.70 |
| EC78426 | R2 | 2.97 | 21.70 | 97.33 | 92.94 | 131 | 131-131 | 35-70 | 2-37 | 37.37 |
| EC78426 | R1 |  |  | 96.58 | 91.41 | 143 | 143-143 | 35-70 | 2-37 | 37.96 |
| EC78434 | R2 | 4.85 | 35.42 | 97.38 | 93.02 | 131 | 131-131 | 35-70 | 2-37 | 37.67 |
| EC78434 | R1 |  |  | 96.62 | 91.50 | 143 | 143-143 | 35-70 | 2-37 | 38.15 |
| EC78437 | R2 | 6.51 | 47.52 | 97.48 | 93.23 | 131 | 131-131 | 35-70 | 2-37 | 37.50 |
| EC78437 | R1 |  |  | 96.63 | 91.50 | 143 | 143-143 | 35-70 | 2-37 | 37.92 |
| EC78438 | R2 | 9.66 | 70.52 | 97.36 | 93.00 | 131 | 131-131 | 35-70 | 2-37 | 37.25 |
| EC78438 | R1 |  |  | 96.55 | 91.33 | 143 | 143-143 | 35-70 | 2-37 | 37.76 |
| EC78441-B | R2 | 4.58 | 33.42 | 97.33 | 92.98 | 131 | 131-131 | 35-70 | 2-37 | 37.43 |
| EC78441-B | R1 |  |  | 96.48 | 91.17 | 143 | 143-143 | 35-70 | 2-37 | 38.01 |
| EC78446 | R2 | 5.20 | 37.94 | 97.38 | 93.03 | 131 | 131-131 | 35-70 | 2-37 | 37.20 |
| EC78446 | R1 |  |  | 96.64 | 91.50 | 143 | 143-143 | 35-70 | 2-37 | 37.76 |
| EC78453 | R2 | 4.20 | 30.67 | 97.48 | 93.08 | 131 | 131-131 | 35-70 | 2-37 | 37.44 |
| EC78453 | R1 |  |  | 96.90 | 92.13 | 143 | 143-143 | 35-70 | 2-37 | 37.89 |
| EC78459 | R2 | 8.18 | 59.67 | 97.48 | 93.06 | 131 | 131-131 | 35-70 | 2-37 | 37.34 |
| EC78459 | R1 |  |  | 95.67 | 89.60 | 143 | 143-143 | 35-70 | 2-37 | 38.04 |
| EC78472 | R2 | 0.01 | 0.09 | 93.91 | 86.37 | 131 | 131-131 | 35-70 | 2-37 | 38.28 |
| EC78472 | R1 |  |  | 94.68 | 87.59 | 143 | 143-143 | 35-70 | 2-37 | 38.91 |
| EC78474 | R2 | 0.02 | 0.13 | 93.94 | 86.31 | 131 | 131-131 | 35-70 | 2-37 | 38.85 |
| EC78474 | R1 |  |  | 95.02 | 88.32 | 143 | 143-143 | 35-70 | 2-37 | 39.24 |
| EC78476 | R2 | 0.03 | 0.20 | 95.24 | 88.88 | 131 | 131-131 | 35-70 | 2-37 | 37.92 |
| EC78476 | R1 |  |  | 95.15 | 88.56 | 143 | 143-143 | 35-70 | 2-37 | 38.43 |
| EC78477-A | R2 | 0.02 | 0.16 | 93.46 | 85.79 | 131 | 131-131 | 35-70 | 2-37 | 38.32 |
| EC78477-A | R1 |  |  | 93.93 | 86.60 | 143 | 143-143 | 35-70 | 2-37 | 38.88 |
| EC78488 | R2 | 7.10 | 51.82 | 97.50 | 93.14 | 131 | 131-131 | 35-70 | 2-37 | 37.59 |
| EC78488 | R1 |  |  | 96.02 | 90.23 | 143 | 143-143 | 35-70 | 2-37 | 37.92 |
| EC78498 | R2 | 9.20 | 67.17 | 97.41 | 92.92 | 131 | 131-131 | 35-70 | 2-37 | 37.61 |
| EC78498 | R1 |  |  | 95.93 | 90.14 | 143 | 143-143 | 35-70 | 2-37 | 37.96 |
| EC78499 | R2 | 0.06 | 0.45 | 95.14 | 89.02 | 131 | 131-131 | 35-70 | 2-37 | 37.58 |
| EC78499 | R1 |  |  | 94.45 | 87.56 | 143 | 143-143 | 44-70 | 11-37 | 38.32 |
| EC78505 | R2 | 0.03 | 0.20 | 93.54 | 85.89 | 131 | 131-131 | 35-70 | 2-37 | 38.39 |
| EC78505 | R1 |  |  | 93.86 | 86.24 | 143 | 143-143 | 35-70 | 2-37 | 38.99 |
| EC78506 | R2 | 0.14 | 0.99 | 96.45 | 91.14 | 131 | 131-131 | 35-70 | 2-37 | 37.49 |
| EC78506 | R1 |  |  | 95.40 | 89.18 | 143 | 143-143 | 35-70 | 2-37 | 38.08 |
| EC78509 | R2 | 4.84 | 35.34 | 97.45 | 93.00 | 131 | 131-131 | 35-70 | 2-37 | 37.65 |
| EC78509 | R1 |  |  | 95.78 | 89.86 | 143 | 143-143 | 35-70 | 2-37 | 38.15 |
| EC78511 | R2 | 9.26 | 67.62 | 97.37 | 92.95 | 131 | 131-131 | 35-70 | 2-37 | 36.96 |
| EC78511 | R1 |  |  | 95.57 | 89.32 | 143 | 143-143 | 35-70 | 2-37 | 37.86 |
| EC78513 | R2 | 6.26 | 45.71 | 97.43 | 93.02 | 131 | 131-131 | 35-70 | 2-37 | 37.52 |
| EC78513 | R1 |  |  | 95.72 | 89.73 | 143 | 143-143 | 35-70 | 2-37 | 38.10 |
| EC78528 | R2 | 4.46 | 32.52 | 97.20 | 92.74 | 131 | 131-131 | 35-70 | 2-37 | 37.42 |
| EC78528 | R1 |  |  | 96.40 | 91.01 | 143 | 143-143 | 35-70 | 2-37 | 38.08 |
| EC78529 | R2 | 4.47 | 32.66 | 97.25 | 92.84 | 131 | 131-131 | 35-70 | 2-37 | 37.49 |
| EC78529 | R1 |  |  | 96.45 | 91.11 | 143 | 143-143 | 35-70 | 2-37 | 38.09 |
| EC78532 | R2 | 3.37 | 24.58 | 97.33 | 93.03 | 131 | 131-131 | 35-70 | 2-37 | 37.54 |
| EC78532 | R1 |  |  | 96.53 | 91.32 | 143 | 143-143 | 35-70 | 2-37 | 37.91 |
| EC78539 | R2 | 4.84 | 35.32 | 97.13 | 92.61 | 131 | 131-131 | 35-70 | 2-37 | 37.57 |
| EC78539 | R1 |  |  | 96.42 | 91.09 | 143 | 143-143 | 35-70 | 2-37 | 38.05 |
| EC78541-A | R2 | 5.33 | 38.91 | 97.46 | 93.19 | 131 | 131-131 | 35-70 | 2-37 | 37.31 |
| EC78541-A | R1 |  |  | 96.68 | 91.58 | 143 | 143-143 | 35-70 | 2-37 | 37.76 |
| EC78542-A | R2 | 0.02 | 0.12 | 94.18 | 86.94 | 131 | 131-131 | 35-70 | 2-37 | 38.74 |
| EC78542-A | R1 |  |  | 94.61 | 87.59 | 143 | 143-143 | 35-70 | 2-37 | 39.23 |
| EC78543 | R2 | 4.42 | 32.29 | 97.36 | 92.93 | 131 | 131-131 | 35-70 | 2-37 | 37.65 |
| EC78543 | R1 |  |  | 96.72 | 91.61 | 143 | 143-143 | 35-70 | 2-37 | 37.98 |
| EC78545 | R2 | 3.90 | 28.49 | 97.62 | 93.49 | 131 | 131-131 | 35-70 | 2-37 | 37.91 |
| EC78545 | R1 |  |  | 96.73 | 91.67 | 143 | 143-143 | 35-70 | 2-37 | 38.19 |
| EC78551-A | R2 | 0.03 | 0.22 | 95.08 | 88.56 | 131 | 131-131 | 35-70 | 2-37 | 37.84 |
| EC78551-A | R1 |  |  | 94.87 | 87.95 | 143 | 143-143 | 35-70 | 2-37 | 38.51 |
| EC78554 | R2 | 4.62 | 33.70 | 97.51 | 93.20 | 131 | 131-131 | 35-70 | 2-37 | 37.14 |
| EC78554 | R1 |  |  | 96.69 | 91.57 | 143 | 143-143 | 35-70 | 2-37 | 37.70 |
| EC78933 | R2 | 4.95 | 36.15 | 97.22 | 92.82 | 131 | 131-131 | 35-70 | 2-37 | 37.43 |
| EC78933 | R1 |  |  | 96.37 | 90.96 | 143 | 143-143 | 35-70 | 2-37 | 38.06 |
| IC346092 | R2 | 3.02 | 22.02 | 97.36 | 92.97 | 131 | 131-131 | 35-70 | 2-37 | 37.92 |
| IC346092 | R1 |  |  | 96.62 | 91.55 | 143 | 143-143 | 35-70 | 2-37 | 38.18 |
| IC560181 | R2 | 3.88 | 28.29 | 97.39 | 93.04 | 131 | 131-131 | 35-70 | 2-37 | 37.22 |
| IC560181 | R1 |  |  | 96.64 | 91.53 | 143 | 143-143 | 35-70 | 2-37 | 37.79 |
| IG69568 | R2 | 2.28 | 16.63 | 97.40 | 93.03 | 131 | 131-131 | 35-70 | 2-37 | 37.63 |
| IG69568 | R1 |  |  | 96.64 | 91.54 | 143 | 143-143 | 35-70 | 2-37 | 38.13 |
| ILL10832 | R2 | 4.97 | 36.30 | 97.34 | 93.01 | 131 | 131-131 | 35-70 | 2-37 | 37.25 |
| ILL10832 | R1 |  |  | 96.46 | 91.09 | 143 | 143-143 | 35-70 | 2-37 | 37.89 |
| ILWL118 | R2 | 4.15 | 30.27 | 97.29 | 92.90 | 131 | 131-131 | 35-70 | 2-37 | 37.36 |
| ILWL118 | R1 |  |  | 96.47 | 91.09 | 143 | 143-143 | 35-70 | 2-37 | 38.00 |
| IPL321 | R2 | 3.25 | 23.75 | 97.51 | 93.20 | 131 | 131-131 | 35-70 | 2-37 | 37.72 |
| IPL321 | R1 |  |  | 96.86 | 91.99 | 143 | 143-143 | 35-70 | 2-37 | 38.05 |
| KLS218 | R2 | 4.14 | 30.20 | 97.39 | 93.05 | 131 | 131-131 | 35-70 | 2-37 | 37.14 |
| KLS218 | R1 |  |  | 96.58 | 91.38 | 143 | 143-143 | 35-70 | 2-37 | 37.71 |
| L11-223 | R2 | 2.75 | 20.06 | 97.40 | 93.11 | 131 | 131-131 | 35-70 | 2-37 | 37.99 |
| L11-223 | R1 |  |  | 96.64 | 91.57 | 143 | 143-143 | 35-70 | 2-37 | 38.30 |
| L11-292 | R2 | 3.51 | 25.60 | 97.57 | 93.35 | 131 | 131-131 | 35-70 | 2-37 | 38.20 |
| L11-292 | R1 |  |  | 96.88 | 92.09 | 143 | 143-143 | 35-70 | 2-37 | 38.36 |
| L4649 | R2 | 3.55 | 25.94 | 97.56 | 93.35 | 131 | 131-131 | 35-70 | 2-37 | 38.24 |
| L4649 | R1 |  |  | 96.82 | 91.97 | 143 | 143-143 | 35-70 | 2-37 | 38.31 |
| L7920 | R2 | 4.40 | 32.12 | 97.35 | 92.99 | 131 | 131-131 | 35-70 | 2-37 | 37.26 |
| L7920 | R1 |  |  | 96.59 | 91.37 | 143 | 143-143 | 35-70 | 2-37 | 37.84 |
| LH7-26 | R2 | 4.35 | 31.75 | 97.45 | 93.22 | 131 | 131-131 | 35-70 | 2-37 | 38.23 |
| LH7-26 | R1 |  |  | 96.65 | 91.60 | 143 | 143-143 | 35-70 | 2-37 | 38.33 |
| LL1122 | R2 | 4.47 | 32.63 | 97.30 | 92.83 | 131 | 131-131 | 35-70 | 2-37 | 37.94 |
| LL1122 | R1 |  |  | 96.67 | 91.67 | 143 | 143-143 | 35-70 | 2-37 | 38.17 |
| MC6 | R2 | 3.34 | 24.41 | 97.25 | 92.89 | 131 | 131-131 | 35-70 | 2-37 | 37.66 |
| MC6 | R1 |  |  | 96.48 | 91.13 | 143 | 143-143 | 35-70 | 2-37 | 38.29 |
| NDL1 | R2 | 0.19 | 1.36 | 97.53 | 93.12 | 131 | 131-131 | 35-70 | 2-37 | 37.60 |
| NDL1 | R1 |  |  | 96.92 | 92.22 | 143 | 143-143 | 35-70 | 2-37 | 37.77 |
| P117 | R2 | 3.34 | 24.35 | 97.40 | 93.08 | 131 | 131-131 | 35-70 | 2-37 | 37.73 |
| P117 | R1 |  |  | 96.63 | 91.51 | 143 | 143-143 | 35-70 | 2-37 | 38.10 |
| P13107 | R2 | 4.29 | 31.31 | 96.79 | 91.82 | 131 | 131-131 | 35-70 | 2-37 | 37.62 |
| P13107 | R1 |  |  | 96.91 | 92.16 | 143 | 143-143 | 35-70 | 2-37 | 38.02 |
| P13108 | R2 | 3.60 | 26.28 | 97.45 | 93.15 | 131 | 131-131 | 35-70 | 2-37 | 37.96 |
| P13108 | R1 |  |  | 96.65 | 91.58 | 143 | 143-143 | 35-70 | 2-37 | 38.25 |
| P13109 | R2 | 4.72 | 34.45 | 97.62 | 93.37 | 131 | 131-131 | 35-70 | 2-37 | 37.74 |
| P13109 | R1 |  |  | 96.91 | 92.13 | 143 | 143-143 | 35-70 | 2-37 | 38.01 |
| P13112 | R2 | 5.04 | 36.82 | 97.31 | 92.96 | 131 | 131-131 | 35-70 | 2-37 | 37.45 |
| P13112 | R1 |  |  | 96.45 | 91.09 | 143 | 143-143 | 35-70 | 2-37 | 38.04 |
| P13115 | R2 | 3.95 | 28.81 | 97.45 | 93.17 | 131 | 131-131 | 35-70 | 2-37 | 37.70 |
| P13115 | R1 |  |  | 96.64 | 91.54 | 143 | 143-143 | 35-70 | 2-37 | 38.13 |
| P13119 | R2 | 5.42 | 39.59 | 97.47 | 93.16 | 131 | 131-131 | 35-70 | 2-37 | 37.48 |
| P13119 | R1 |  |  | 96.62 | 91.43 | 143 | 143-143 | 35-70 | 2-37 | 38.02 |
| P13122 | R2 | 2.35 | 17.14 | 97.20 | 92.64 | 131 | 131-131 | 35-70 | 2-37 | 37.37 |
| P13122 | R1 |  |  | 96.58 | 91.33 | 143 | 143-143 | 35-70 | 2-37 | 37.95 |
| P13123 | R2 | 4.13 | 30.17 | 97.36 | 92.95 | 131 | 131-131 | 35-70 | 2-37 | 37.80 |
| P13123 | R1 |  |  | 96.73 | 91.75 | 143 | 143-143 | 35-70 | 2-37 | 38.14 |
| P13128 | R2 | 3.90 | 28.50 | 97.21 | 92.68 | 131 | 131-131 | 35-70 | 2-37 | 37.51 |
| P13128 | R1 |  |  | 96.55 | 91.28 | 143 | 143-143 | 35-70 | 2-37 | 38.02 |
| P13130 | R2 | 3.50 | 25.56 | 97.51 | 93.19 | 131 | 131-131 | 35-70 | 2-37 | 38.04 |
| P13130 | R1 |  |  | 96.86 | 92.06 | 143 | 143-143 | 35-70 | 2-37 | 38.23 |
| P13131 | R2 | 4.21 | 30.71 | 97.33 | 92.98 | 131 | 131-131 | 35-70 | 2-37 | 37.29 |
| P13131 | R1 |  |  | 96.50 | 91.19 | 143 | 143-143 | 35-70 | 2-37 | 37.89 |
| P13133 | R2 | 4.94 | 36.06 | 97.26 | 92.91 | 131 | 131-131 | 35-70 | 2-37 | 37.60 |
| P13133 | R1 |  |  | 96.34 | 90.92 | 143 | 143-143 | 35-70 | 2-37 | 38.18 |
| P13135 | R2 | 3.91 | 28.55 | 97.50 | 93.28 | 131 | 131-131 | 35-70 | 2-37 | 37.52 |
| P13135 | R1 |  |  | 96.63 | 91.42 | 143 | 143-143 | 35-70 | 2-37 | 37.96 |
| P13138 | R2 | 2.94 | 21.48 | 97.38 | 93.01 | 131 | 131-131 | 35-70 | 2-37 | 37.70 |
| P13138 | R1 |  |  | 96.54 | 91.37 | 143 | 143-143 | 35-70 | 2-37 | 38.16 |
| P13142 | R2 | 6.78 | 49.51 | 97.48 | 93.22 | 131 | 131-131 | 35-70 | 2-37 | 37.79 |
| P13142 | R1 |  |  | 96.71 | 91.70 | 143 | 143-143 | 35-70 | 2-37 | 38.04 |
| P13143 | R2 | 3.48 | 25.40 | 97.45 | 93.16 | 131 | 131-131 | 35-70 | 2-37 | 38.18 |
| P13143 | R1 |  |  | 96.70 | 91.70 | 143 | 143-143 | 35-70 | 2-37 | 38.38 |
| P13145 | R2 | 4.77 | 34.83 | 97.35 | 93.01 | 131 | 131-131 | 35-70 | 2-37 | 37.76 |
| P13145 | R1 |  |  | 96.52 | 91.29 | 143 | 143-143 | 35-70 | 2-37 | 38.12 |
| P13157 | R2 | 3.51 | 25.65 | 97.42 | 93.09 | 131 | 131-131 | 35-70 | 2-37 | 37.65 |
| P13157 | R1 |  |  | 96.68 | 91.62 | 143 | 143-143 | 35-70 | 2-37 | 38.00 |
| P14105 | R2 | 4.52 | 33.03 | 97.46 | 93.18 | 131 | 131-131 | 35-70 | 2-37 | 37.73 |
| P14105 | R1 |  |  | 96.71 | 91.62 | 143 | 143-143 | 35-70 | 2-37 | 38.07 |
| P14109 | R2 | 5.46 | 39.83 | 97.47 | 93.14 | 131 | 131-131 | 35-70 | 2-37 | 37.88 |
| P14109 | R1 |  |  | 96.90 | 92.14 | 143 | 143-143 | 35-70 | 2-37 | 38.08 |
| P14903 | R2 | 3.92 | 28.58 | 97.32 | 92.94 | 131 | 131-131 | 35-70 | 2-37 | 37.49 |
| P14903 | R1 |  |  | 96.64 | 91.39 | 143 | 143-143 | 35-70 | 2-37 | 38.01 |
| P15104 | R2 | 2.28 | 16.65 | 97.31 | 92.91 | 131 | 131-131 | 35-70 | 2-37 | 37.53 |
| P15104 | R1 |  |  | 96.68 | 91.57 | 143 | 143-143 | 35-70 | 2-37 | 38.14 |
| P15111 | R2 | 3.10 | 22.66 | 97.48 | 93.22 | 131 | 131-131 | 35-70 | 2-37 | 37.55 |
| P15111 | R1 |  |  | 96.61 | 91.42 | 143 | 143-143 | 35-70 | 2-37 | 38.07 |
| P15115 | R2 | 4.80 | 35.01 | 97.31 | 92.86 | 131 | 131-131 | 35-70 | 2-37 | 37.72 |
| P15115 | R1 |  |  | 96.67 | 91.63 | 143 | 143-143 | 35-70 | 2-37 | 38.08 |
| P15121 | R2 | 3.20 | 23.37 | 97.42 | 93.13 | 131 | 131-131 | 35-70 | 2-37 | 37.64 |
| P15121 | R1 |  |  | 96.71 | 91.62 | 143 | 143-143 | 35-70 | 2-37 | 38.02 |
| P15207 | R2 | 3.84 | 28.02 | 97.50 | 93.23 | 131 | 131-131 | 35-70 | 2-37 | 37.58 |
| P15207 | R1 |  |  | 96.69 | 91.58 | 143 | 143-143 | 35-70 | 2-37 | 38.04 |
| P15213 | R2 | 3.66 | 26.71 | 97.33 | 92.91 | 131 | 131-131 | 35-70 | 2-37 | 37.42 |
| P15213 | R1 |  |  | 96.7 | 91.68 | 143 | 143-143 | 35-70 | 2-37 | 37.97 |
| P16205 | R2 | 2.09 | 15.26 | 97.39 | 93.07 | 131 | 131-131 | 35-70 | 2-37 | 37.31 |
| P16205 | R1 |  |  | 96.60 | 91.46 | 143 | 143-143 | 35-70 | 2-37 | 37.78 |
| P3234 | R2 | 3.85 | 28.12 | 97.52 | 93.32 | 131 | 131-131 | 35-70 | 2-37 | 37.69 |
| P3234 | R1 |  |  | 96.68 | 91.60 | 143 | 143-143 | 35-70 | 2-37 | 38.06 |
| P3235 | R2 | 4.31 | 31.45 | 97.38 | 92.99 | 131 | 131-131 | 35-70 | 2-37 | 37.52 |
| P3235 | R1 |  |  | 96.63 | 91.51 | 143 | 143-143 | 35-70 | 2-37 | 37.97 |
| P3236 | R2 | 6.01 | 43.86 | 97.38 | 93.02 | 131 | 131-131 | 35-70 | 2-37 | 37.63 |
| P3236 | R1 |  |  | 96.68 | 91.65 | 143 | 143-143 | 35-70 | 2-37 | 37.97 |
| P8103 | R2 | 3.13 | 22.82 | 97.37 | 93.00 | 131 | 131-131 | 35-70 | 2-37 | 37.95 |
| P8103 | R1 |  |  | 96.68 | 91.70 | 143 | 143-143 | 35-70 | 2-37 | 38.31 |
| P8110 | R2 | 3.74 | 27.28 | 97.44 | 93.17 | 131 | 131-131 | 35-70 | 2-37 | 37.59 |
| P8110 | R1 |  |  | 96.61 | 91.45 | 143 | 143-143 | 35-70 | 2-37 | 37.99 |
| P8112 | R2 | 4.45 | 32.45 | 97.42 | 93.09 | 131 | 131-131 | 35-70 | 2-37 | 37.36 |
| P8112 | R1 |  |  | 96.71 | 91.61 | 143 | 143-143 | 35-70 | 2-37 | 37.86 |
| PL639 | R2 | 5.41 | 39.46 | 97.62 | 93.44 | 131 | 131-131 | 35-70 | 2-37 | 38.01 |
| PL639 | R1 |  |  | 96.94 | 92.19 | 143 | 143-143 | 35-70 | 2-37 | 38.11 |
| PL97 | R2 | 0.09 | 0.66 | 96.84 | 91.67 | 131 | 131-131 | 35-70 | 2-37 | 36.90 |
| PL97 | R1 |  |  | 96.84 | 91.92 | 143 | 143-143 | 35-70 | 2-37 | 37.58 |
|  | **Avg.** | **3.97** | **29.00** | **96.51** | **91.33** | **137.00** |  |  |  | **37.89** |

| **Table S5:** Summary and distribution of SNPs on lentil chromosomes | | | | |
| --- | --- | --- | --- | --- |
| **Chromosome** | **Total length (Mb)** | **SNPs** | **% Variants** | **Variants rate/Mb** |
| Chr 1 | 537.78 | 4900 | 14.00 | 109.75 |
| Chr 2 | 613.58 | 6055 | 17.30 | 101.33 |
| Chr 3 | 430.03 | 5294 | 15.13 | 81.23 |
| Chr 4 | 481.87 | 4226 | 12.08 | 114.02 |
| Chr 5 | 474.41 | 4329 | 12.37 | 109.59 |
| Chr 6 | 420.53 | 5323 | 15.21 | 79.00 |
| Chr 7 | 529.02 | 4868 | 13.91 | 108.67 |
| **Total** | **3,487** | **34,995** | **100** | **100.51** |

| **Table S6:** Pedigree information of 142 lentil accessions and their clustering patterns based on 34,995 SNPs | | | | |
| --- | --- | --- | --- | --- |
| **Sr. No** | **Genotype** | **Cluster** | **Pedigree/Key feature/Method** | **Origin** |
| 1 | EC78437 | Cluster I | Exotic germplasm | ICARDA, Aleppo, Syria |
| 2 | L7920 | Cluster I | Resistance to Fusarium | IARI, New Delhi, India |
| 3 | EC78539 | Cluster I | Exotic germplasm | ICARDA, Aleppo, Syria |
| 4 | EC223212-A | Cluster I | Exotic germplasm | ICARDA, Aleppo, Syria |
| 5 | P13131 | Cluster I | ICARDA Nursery selection | ICARDA, Aleppo, Syria |
| 6 | P13145 | Cluster I | ICARDA Nursery selection | ICARDA, Aleppo, Syria |
| 7 | L11-292 | Cluster I | Advanced breeding lines | IIPR, Kanpur India |
| 8 | MC6 | Cluster I | Advanced breeding lines | India |
| 9 | P117 | Cluster I | Advanced breeding lines | GBPUAT, Pantnagar, India |
| 10 | P13130 | Cluster I | ICARDA Nursery selection | ICARDA, Aleppo, Syria |
| 11 | EC223150 | Cluster I | Exotic germplasm | ICARDA, Aleppo, Syria |
| 12 | EC78498 | Cluster I | Exotic germplasm | ICARDA, Aleppo, Syria |
| 13 | EC267569-A | Cluster I | Exotic germplasm | ICARDA, Aleppo, Syria |
| 14 | EC78528 | Cluster I | Exotic germplasm | ICARDA, Aleppo, Syria |
| 15 | EC78529 | Cluster I | Exotic germplasm | ICARDA, Aleppo, Syria |
| 16 | EC267569-B | Cluster I | Exotic germplasm | ICARDA, Aleppo, Syria |
| 17 | EC78541-A | Cluster I | Exotic germplasm | ICARDA, Aleppo, Syria |
| 18 | IC346092 | Cluster I | High Zn content | NBPGR, New Delhi, India |
| 19 | EC78441-B | Cluster I | Exotic germplasm | ICARDA, Aleppo, Syria |
| 20 | EC78532 | Cluster I | Exotic germplasm | ICARDA, Aleppo, Syria |
| 21 | EC78543 | Cluster I | Exotic germplasm | ICARDA, Aleppo, Syria |
| 22 | EC267595-C | Cluster I | Exotic germplasm | ICARDA, Aleppo, Syria |
| 23 | EC223197-B | Cluster I | Exotic germplasm | ICARDA, Aleppo, Syria |
| 24 | EC78425 | Cluster I | Exotic germplasm | ICARDA, Aleppo, Syria |
| 25 | EC78476 | Cluster I | Exotic germplasm | ICARDA, Aleppo, Syria |
| 26 | EC78477-A | Cluster I | Exotic germplasm | ICARDA, Aleppo, Syria |
| 27 | EC267628-A | Cluster I | Exotic germplasm | ICARDA, Aleppo, Syria |
| 28 | EC78542-A | Cluster I | Exotic germplasm | ICARDA, Aleppo, Syria |
| 29 | EC78505 | Cluster I | Exotic germplasm | ICARDA, Aleppo, Syria |
| 30 | EC267567 | Cluster I | Exotic germplasm | ICARDA, Aleppo, Syria |
| 31 | EC78414 | Cluster I | Exotic germplasm | ICARDA, Aleppo, Syria |
| 32 | EC78438 | Cluster I | Exotic germplasm | ICARDA, Aleppo, Syria |
| 33 | EC78488 | Cluster I | Exotic germplasm | ICARDA, Aleppo, Syria |
| 34 | EC78459 | Cluster I | Exotic germplasm | ICARDA, Aleppo, Syria |
| 35 | EC78551-A | Cluster I | Exotic germplasm | ICARDA, Aleppo, Syria |
| 36 | EC223188 | Cluster I | Exotic germplasm | ICARDA, Aleppo, Syria |
| 37 | EC223209-B | Cluster I | Exotic germplasm | ICARDA, Aleppo, Syria |
| 38 | EC223223 | Cluster I | Exotic germplasm | ICARDA, Aleppo, Syria |
| 39 | EC267605 | Cluster I | Exotic germplasm | ICARDA, Aleppo, Syria |
| 40 | EC267573 | Cluster I | Exotic germplasm | ICARDA, Aleppo, Syria |
| 41 | EC225501 | Cluster I | Exotic germplasm | ICARDA, Aleppo, Syria |
| 42 | EC267539 | Cluster I | Exotic germplasm | ICARDA, Aleppo, Syria |
| 43 | EC78511 | Cluster I | Exotic germplasm | ICARDA, Aleppo, Syria |
| 44 | EC267636 | Cluster I | Exotic germplasm | ICARDA, Aleppo, Syria |
| 45 | EC267638 | Cluster I | Exotic germplasm | ICARDA, Aleppo, Syria |
| 46 | EC139824-A | Cluster I | Exotic germplasm | ICARDA, Aleppo, Syria |
| 47 | EC78472 | Cluster I | Exotic germplasm | ICARDA, Aleppo, Syria |
| 48 | EC78513 | Cluster I | Exotic germplasm | ICARDA, Aleppo, Syria |
| 49 | EC255491 | Cluster I | Exotic germplasm | ICARDA, Aleppo, Syria |
| 50 | EC223397 | Cluster I | Exotic germplasm | ICARDA, Aleppo, Syria |
| 51 | EC78446 | Cluster I | Exotic germplasm | ICARDA, Aleppo, Syria |
| 52 | L4649 | Cluster I | Advanced breeding lines | IARI, New Delhi, India |
| 53 | IC 560181 | Cluster I | Indian germplasm lines | NBPGR, New Delhi, India |
| 54 | IG69568 | Cluster I | Advanced breeding lines | ICARDA, Aleppo, Syria |
| 55 | EC223197-A | Cluster I | Exotic germplasm | ICARDA, Aleppo, Syria |
| 56 | EC78554 | Cluster I | Exotic germplasm | ICARDA, Aleppo, Syria |
| 57 | EC223230 | Cluster I | Exotic germplasm | ICARDA, Aleppo, Syria |
| 58 | EC223207 | Cluster I | Exotic germplasm | ICARDA, Aleppo, Syria |
| 59 | EC223229-B | Cluster I | Exotic germplasm | ICARDA, Aleppo, Syria |
| 60 | EC267533 | Cluster I | Exotic germplasm | ICARDA, Aleppo, Syria |
| 61 | EC267563 | Cluster I | Exotic germplasm | ICARDA, Aleppo, Syria |
| 62 | EC267536 | Cluster I | Exotic germplasm | ICARDA, Aleppo, Syria |
| 63 | EC267554 | Cluster I | Exotic germplasm | ICARDA, Aleppo, Syria |
| 64 | EC267557-D | Cluster I | Exotic germplasm | ICARDA, Aleppo, Syria |
| 65 | EC267540 | Cluster I | Exotic germplasm | ICARDA, Aleppo, Syria |
| 66 | EC267545-D | Cluster I | Exotic germplasm | ICARDA, Aleppo, Syria |
| 67 | EC78453 | Cluster I | Exotic germplasm | ICARDA, Aleppo, Syria |
| 68 | EC78474 | Cluster I | Exotic germplasm | ICARDA, Aleppo, Syria |
| 69 | EC223220 | Cluster I | Exotic germplasm | ICARDA, Aleppo, Syria |
| 70 | EC267529 | Cluster I | Exotic germplasm | ICARDA, Aleppo, Syria |
| 71 | IPL321 | Cluster I | Resistance to Wilt | IIPR, Kanpur India |
| 72 | PL639 | Cluster I | high yielder with more protein percentage | GBPUAT, Pantnagar, India |
| 73 | EC223215 | Cluster I | Exotic germplasm | ICARDA, Aleppo, Syria |
| 74 | EC78426 | Cluster I | Exotic germplasm | ICARDA, Aleppo, Syria |
| 75 | PL97 | Cluster I | Advanced breeding lines | GBPUAT, Pantnagar, India |
| 76 | EC223205-B | Cluster I | Exotic germplasm | ICARDA, Aleppo, Syria |
| 77 | EC267696 | Cluster I | Exotic germplasm | ICARDA, Aleppo, Syria |
| 78 | EC267676 | Cluster I | Exotic germplasm | ICARDA, Aleppo, Syria |
| 79 | 10-3-1-26 | Cluster II | Advanced breeding lines | IARI, New Delhi, India |
| 80 | LL1122 | Cluster II | Elite breeding line (DPL15 × No. 303) | India |
| 81 | EC223242 | Cluster II | Exotic germplasm | ICARDA, Aleppo, Syria |
| 82 | NDL1 | Cluster II | High Yielding variety | India |
| 83 | EC78545 | Cluster II | Exotic germplasm | ICARDA, Aleppo, Syria |
| 84 | EC329166 | Cluster II | Exotic germplasm | ICARDA, Aleppo, Syria |
| 85 | EC267709 | Cluster II | Exotic germplasm | ICARDA, Aleppo, Syria |
| 86 | DPL15 | Cluster II | Resistance to rust disease | IIPR, Kanpur India |
| 87 | EC78408 | Cluster II | Exotic germplasm | ICARDA, Aleppo, Syria |
| 88 | EC267604 | Cluster II | Exotic germplasm | ICARDA, Aleppo, Syria |
| 89 | EC78506 | Cluster II | Exotic germplasm | ICARDA, Aleppo, Syria |
| 90 | EC267544-A | Cluster II | Exotic germplasm | ICARDA, Aleppo, Syria |
| 91 | ILWL118 | Cluster II | Advanced breeding lines | IARI, New Delhi, India |
| 92 | EC78395 | Cluster II | Exotic germplasm | ICARDA, Aleppo, Syria |
| 93 | EC78434 | Cluster II | Exotic germplasm | ICARDA, Aleppo, Syria |
| 94 | EC223222 | Cluster II | Exotic germplasm | ICARDA, Aleppo, Syria |
| 95 | KLS218 | Cluster II | Resistance to rust disease | CSAU, Kanpur, India |
| 96 | P8112 | Cluster II | ICARDA Nursery selection | ICARDA, Aleppo, Syria |
| 97 | L11-223 | Cluster II | Advanced breeding lines | IIPR, Kanpur India |
| 98 | P14109 | Cluster II | ICARDA Nursery selection | ICARDA, Aleppo, Syria |
| 99 | LH7-26 | Cluster II | Resistance to Wilt | India |
| 100 | P13107 | Cluster II | ICARDA Nursery selection | ICARDA, Aleppo, Syria |
| 101 | P13143 | Cluster II | ICARDA Nursery selection | ICARDA, Aleppo, Syria |
| 102 | P13109 | Cluster II | ICARDA Nursery selection | ICARDA, Aleppo, Syria |
| 103 | P13122 | Cluster II | ICARDA Nursery selection | ICARDA, Aleppo, Syria |
| 104 | P13138 | Cluster II | ICARDA Nursery selection | ICARDA, Aleppo, Syria |
| 105 | P14903 | Cluster II | ICARDA Nursery selection | ICARDA, Aleppo, Syria |
| 106 | P13128 | Cluster II | ICARDA Nursery selection | ICARDA, Aleppo, Syria |
| 107 | P14105 | Cluster II | ICARDA Nursery selection | ICARDA, Aleppo, Syria |
| 108 | P13133 | Cluster II | Advanced breeding lines | ICARDA, Aleppo, Syria |
| 109 | P13135 | Cluster II | ICARDA Nursery selection | ICARDA, Aleppo, Syria |
| 110 | ILL10832 | Cluster III | Mediterranean landraces | ICARDA, Aleppo, Syria |
| 111 | P3235 | Cluster III | ICARDA Nursery selection | ICARDA, Aleppo, Syria |
| 112 | P13108 | Cluster III | ICARDA Nursery selection | ICARDA, Aleppo, Syria |
| 113 | P13142 | Cluster III | ICARDA Nursery selection | ICARDA, Aleppo, Syria |
| 114 | P13115 | Cluster III | ICARDA Nursery selection | ICARDA, Aleppo, Syria |
| 115 | P13123 | Cluster III | ICARDA Nursery selection | ICARDA, Aleppo, Syria |
| 116 | P15115 | Cluster III | ICARDA Nursery selection | ICARDA, Aleppo, Syria |
| 117 | P15207 | Cluster III | ICARDA Nursery selection | ICARDA, Aleppo, Syria |
| 118 | P15121 | Cluster III | ICARDA Nursery selection | ICARDA, Aleppo, Syria |
| 119 | P13119 | Cluster III | ICARDA Nursery selection | ICARDA, Aleppo, Syria |
| 120 | P3236 | Cluster III | ICARDA Nursery selection | ICARDA, Aleppo, Syria |
| 121 | P8110 | Cluster III | ICARDA Nursery selection | ICARDA, Aleppo, Syria |
| 122 | P15104 | Cluster III | ICARDA Nursery selection | ICARDA, Aleppo, Syria |
| 123 | P16205 | Cluster III | ICARDA Nursery selection | ICARDA, Aleppo, Syria |
| 124 | P15213 | Cluster III | ICARDA Nursery selection | ICARDA, Aleppo, Syria |
| 125 | P13112 | Cluster III | ICARDA Nursery selection | ICARDA, Aleppo, Syria |
| 126 | P15111 | Cluster III | ICARDA Nursery selection | ICARDA, Aleppo, Syria |
| 127 | P8103 | Cluster III | ICARDA Nursery selection | ICARDA, Aleppo, Syria |
| 128 | EC223199-B | Cluster III | Exotic germplasm | ICARDA, Aleppo, Syria |
| 129 | EC223201 | Cluster III | Exotic germplasm | ICARDA, Aleppo, Syria |
| 130 | EC78499 | Cluster III | Exotic germplasm | ICARDA, Aleppo, Syria |
| 131 | EC78509 | Cluster III | Exotic germplasm | ICARDA, Aleppo, Syria |
| 132 | EC2675770 | Cluster III | Exotic germplasm | ICARDA, Aleppo, Syria |
| 133 | P3234 | Cluster III | ICARDA Nursery selection | ICARDA, Aleppo, Syria |
| 134 | EC267634 | Cluster III | Exotic germplasm | ICARDA, Aleppo, Syria |
| 135 | P13157 | Cluster III | ICARDA Nursery selection | ICARDA, Aleppo, Syria |
| 136 | EC223191 | Cluster III | Exotic germplasm | ICARDA, Aleppo, Syria |
| 137 | EC78933 | Cluster III | Exotic germplasm | ICARDA, Aleppo, Syria |
| 138 | EC267609 | Cluster III | Exotic germplasm | ICARDA, Aleppo, Syria |
| 139 | EC267641 | Cluster III | Exotic germplasm | ICARDA, Aleppo, Syria |
| 140 | EC267625-C | Cluster III | Exotic germplasm | ICARDA, Aleppo, Syria |
| 141 | EC2675471 | Cluster III | Exotic germplasm | ICARDA, Aleppo, Syria |
| 142 | EC223229-A | Cluster III | Exotic germplasm | ICARDA, Aleppo, Syria |

**Table S7:** Marker–trait association analysis revealed major novel QTLs for complex traits in lentil using two GWAS models (BLINK and FarmCPU) across individual environments (E1–E3) and combined BLUP.

| **Model** | **SNP** | **Chromosome** | **Position (bp)** | **P -value** | **PVE (%)** | **MAF** | **Trait** | **Environment** |
| --- | --- | --- | --- | --- | --- | --- | --- | --- |
| BLINK | Chr1_315275939 | Chr1 | 315275939 | 2.70 ×10^-7^ | 7.90 | 0.21 | HSW | E1 |
|  | Chr2_410777988 | Chr2 | 410777988 | 2.04 ×10^-7^ | 6.08 | 0.25 | PPP | BLUP |
|  | Chr3_106842007 | Chr3 | 106842007 | 1.08 ×10^-9^ | 26.08 | 0.41 | DF | E1 |
|  | Chr3_106842007 | Chr3 | 106842007 | 2.86 ×10^-11^ | 39.34 | 0.41 | DF | E3 |
|  | Chr3_106842007 | Chr3 | 106842007 | 4.55 ×10^-9^ | 43.55 | 0.41 | DF | BLUP |
|  | Chr3_163495081 | Chr3 | 163495081 | 5.61 ×10^-07^ | 8.23 | 0.31 | PPP | BLUP |
|  | Chr3_181072527 | Chr3 | 181072527 | 2.70 ×10^-8^ | 35.02 | 0.14 | PH | E1 |
|  | Chr4_447360607 | Chr4 | 447360607 | 4.75 ×10^-14^ | 56.28 | 0.07 | HSW | E1 |
|  | Chr5_138162617 | Chr5 | 138162617 | 6.17 ×10^-11^ | 46.30 | 0.25 | HSW | E2 |
|  | Chr5_342836807 | Chr5 | 342836807 | 3.00 ×10^-07^ | 34.68 | 0.27 | DF | BLUP |
|  | Chr6_4338291 | Chr6 | 4338291 | 1.65 ×10^-9^ | 86.85 | 0.07 | DM | E3 |
|  | Chr6_4338291 | Chr6 | 4338291 | 1.86 ×10^-9^ | 82.76 | 0.07 | DM | BLUP |
|  | Chr6_110350822 | Chr6 | 110350822 | 1.03 ×10^-8^ | 11.64 | 0.22 | HSW | E1 |
|  | Chr6_117959608 | Chr6 | 117959608 | 1.02 ×10^-07^ | 9.90 | 0.11 | HSW | E1 |
|  | Chr6_128041250 | Chr6 | 128041250 | 1.90 ×10^-7^ | 30.04 | 0.23 | HSW | BLUP |
|  | Chr6_253032265 | Chr6 | 253032265 | 3.69 ×10^-10^ | 47.69 | 0.15 | HSW | BLUP |
|  | Chr6_262648494 | Chr6 | 262648494 | 1.74 ×10^-07^ | 7.61 | 0.23 | YPP | E2 |
|  | Chr6_344313403 | Chr6 | 344313403 | 1.60 ×10^-12^ | 66.17 | 0.07 | PPP | BLUP |
|  | Chr6_344313403 | Chr6 | 344313403 | 2.98 ×10^-9^ | 64.60 | 0.07 | YPP | E2 |
|  | Chr7_42734754 | Chr7 | 42734754 | 9.37 ×10^-8^ | 3.94 | 0.41 | DM | E3 |
|  | Chr7_297717438 | Chr7 | 297717438 | 4.41 ×10^-7^ | 14.11 | 0.25 | PH | E1 |
| FarmCPU | Chr1_72000142 | Chr1 | 72000142 | 1.75 ×10^-7^ | 1.00 | 0.07 | DM | E3 |
|  | Chr1_221418298 | Chr1 | 221418298 | 2.72 ×10^-7^ | 1.01 | 0.24 | PPP | BLUP |
|  | Chr1_396757210 | Chr1 | 396757210 | 1.85 ×10^-7^ | 1.00 | 0.06 | HSW | E2 |
|  | Chr1_482512926 | Chr1 | 482512926 | 8.20 ×10^-8^ | 1.01 | 0.07 | DM | BLUP |
|  | Chr2_13220114 | Chr2 | 13220114 | 2.85 ×10^-7^ | 1.00 | 0.05 | DM | BLUP |
|  | Chr2_159172425 | Chr2 | 159172425 | 5.26 ×10^-7^ | 1.00 | 0.25 | DF | E1 |
|  | Chr2_182480268 | Chr2 | 182480268 | 3.43 ×10^-8^ | 2.58 | 0.42 | DM | BLUP |
|  | Chr2_410777988 | Chr2 | 410777988 | 5.62 ×10^-9^ | 7.40 | 0.25 | PPP | BLUP |
|  | Chr2_484037996 | Chr2 | 484037996 | 3.43 ×10^-7^ | 1.00 | 0.15 | HSW | E2 |
|  | Chr2_521877344 | Chr2 | 521877344 | 2.36 ×10^-7^ | 0.53 | 0.24 | DF | E3 |
|  | Chr2_603974724 | Chr2 | 603974724 | 7.64 ×10^-7^ | 14.87 | 0.33 | HSW | BLUP |
|  | Chr3_90640061 | Chr3 | 90640061 | 7.21 ×10^-10^ | 10.08 | 0.06 | HSW | E2 |
|  | Chr3_106842007 | Chr3 | 106842007 | 1.53 ×10^-10^ | 8.89 | 0.41 | DF | E3 |
|  | Chr3_126687055 | Chr3 | 126687055 | 1.37 ×10^-10^ | 1.04 | 0.47 | PPP | BLUP |
|  | Chr3_184872087 | Chr3 | 184872087 | 6.18 ×10^-8^ | 6.92 | 0.19 | DF | BLUP |
|  | Chr3_274815239 | Chr3 | 274815239 | 1.37 ×10^-8^ | 1.00 | 0.07 | DM | BLUP |
|  | Chr4_121690483 | Chr4 | 121690483 | 5.99 ×10^-9^ | 1.34 | 0.13 | HSW | E2 |
|  | Chr4_128357096 | Chr4 | 128357096 | 2.63 ×10^-9^ | 1.39 | 0.38 | HSW | E2 |
|  | Chr4_139205447 | Chr4 | 139205447 | 1.36 ×10^-6^ | 1.10 | 0.14 | HSW | BLUP |
|  | Chr4_338897743 | Chr4 | 338897743 | 3.84 ×10^-7^ | 1.47 | 0.46 | HSW | E2 |
|  | Chr4_348520593 | Chr4 | 348520593 | 1.99 ×10^-7^ | 1.22 | 0.1 | DF | E1 |
|  | Chr4_348520593 | Chr4 | 348520593 | 1.13 ×10^-7^ | 2.41 | 0.1 | DF | BLUP |
|  | Chr4_447360607 | Chr4 | 447360607 | 7.91 ×10^-8^ | 1.00 | 0.07 | HSW | E1 |
|  | Chr4_466290148 | Chr4 | 466290148 | 6.05 ×10^-9^ | 35.95 | 0.32 | HSW | BLUP |
|  | Chr5_11821350 | Chr5 | 11821350 | 7.36 ×10^-07^ | 1.00 | 0.23 | DF | E1 |
|  | Chr5_342836807 | Chr5 | 342836807 | 9.24 ×10^-8^ | 1.00 | 0.27 | DF | E1 |
|  | Chr5_342836807 | Chr5 | 342836807 | 2.04 ×10^-9^ | 1.00 | 0.27 | DF | BLUP |
|  | Chr5_413492767 | Chr5 | 413492767 | 1.15 ×10^-6^ | 1.07 | 0.39 | HSW | BLUP |
|  | Chr5_471647151 | Chr5 | 471647151 | 4.36 ×10^-7^ | 1.00 | 0.06 | HSW | E1 |
|  | Chr6_4338291 | Chr6 | 4338291 | 9.75 ×10^-10^ | 21.25 | 0.07 | DM | E3 |
|  | Chr6_109937017 | Chr6 | 109937017 | 4.72 ×10^-8^ | 1.00 | 0.07 | HSW | E1 |
|  | Chr6_200603138 | Chr6 | 200603138 | 1.38 ×10^-8^ | 1.00 | 0.3 | DF | E3 |
|  | Chr6_224444128 | Chr6 | 224444128 | 1.30 ×10^-6^ | 24.20 | 0.25 | HSW | E2 |
|  | Chr6_253032265 | Chr6 | 253032265 | 3.61 ×10^-7^ | 1.26 | 0.15 | HSW | E1 |
|  | Chr6_253032265 | Chr6 | 253032265 | 4.20 ×10^-13^ | 6.87 | 0.15 | HSW | BLUP |
|  | Chr6_294199001 | Chr6 | 294199001 | 3.41 ×10^-7^ | 1.00 | 0.11 | DM | E3 |
|  | Chr6_344313403 | Chr6 | 344313403 | 2.52 ×10^-9^ | 1.69 | 0.07 | PPP | BLUP |
|  | Chr7_9794560 | Chr7 | 9794560 | 4.31 ×10^-7^ | 8.04 | 0.17 | HSW | E1 |
|  | Chr7_42734754 | Chr7 | 42734754 | 2.00 ×10^-8^ | 1.00 | 0.41 | DM | E3 |
|  | Chr7_295399575 | Chr7 | 295399575 | 9.74 ×10^-7^ | 1.00 | 0.09 | DF | E3 |
|  | Chr7_337510839 | Chr7 | 337510839 | 1.30 ×10^-6^ | 1.00 | 0.08 | HSW | E1 |
|  | Chr7_338463151 | Chr7 | 338463151 | 5.50 ×10^-7^ | 2.54 | 0.06 | DM | E3 |
|  | Chr7_407008237 | Chr7 | 407008237 | 7.83 ×10^8^ | 10.87 | 0.06 | DM | BLUP |

| **Table S8:** GWAS analysis using the GLM model with 49 unique identified MTAs. | | | | | | | | |
| --- | --- | --- | --- | --- | --- | --- | --- | --- |
| **SNP** | **Chr** | **Position (bp)** | **P-value** | **MAF** | **PVE (%)** | **Trait** | **Environment** | **Model** |
| Chr1_72000142 | Chr1 | 72000142 | 0.00035074 | 0.07 | 8.30 | HSW | BLUP | GLM |
| Chr1_221418298 | Chr1 | 221418298 | 0.00006416 | 0.24 | 9.35 | PPP | BLUP | GLM |
| Chr1_221418298 | Chr1 | 221418298 | 0.00037286 | 0.24 | 13.32 | PPP | E1 | GLM |
| Chr1_221418298 | Chr1 | 221418298 | 0.00066908 | 0.24 | 6.99 | PPP | E2 | GLM |
| Chr1_315275939 | Chr1 | 315275939 | 0.00006296 | 0.21 | 18.61 | HSW | E1 | GLM |
| Chr2_13220114 | Chr2 | 13220114 | 0.00023786 | 0.05 | 15.24 | DM | BLUP | GLM |
| Chr2_13220114 | Chr2 | 13220114 | 0.00045097 | 0.05 | 14.08 | DM | E3 | GLM |
| Chr2_159172425 | Chr2 | 159172425 | 0.00089877 | 0.25 | 6.02 | DF | BLUP | GLM |
| Chr2_159172425 | Chr2 | 159172425 | 0.00079522 | 0.25 | 6.22 | DF | E1 | GLM |
| Chr2_159172425 | Chr2 | 159172425 | 0.00026118 | 0.25 | 7.90 | DF | E2 | GLM |
| Chr2_182480268 | Chr2 | 182480268 | 0.00037533 | 0.42 | 4.46 | DM | BLUP | GLM |
| Chr2_182480268 | Chr2 | 182480268 | 0.00006279 | 0.42 | 18.25 | DM | E2 | GLM |
| Chr2_410777988 | Chr2 | 410777988 | 0.00006990 | 0.25 | 13.91 | PPP | BLUP | GLM |
| Chr2_410777988 | Chr2 | 410777988 | 0.00013731 | 0.25 | 16.81 | PPP | E1 | GLM |
| Chr2_410777988 | Chr2 | 410777988 | 0.00069948 | 0.25 | 15.40 | YPP | E1 | GLM |
| Chr2_410777988 | Chr2 | 410777988 | 0.00051512 | 0.25 | 11.28 | PPP | E2 | GLM |
| Chr3_181072527 | Chr3 | 181072527 | 0.00046042 | 0.14 | 17.97 | PH | E1 | GLM |
| Chr4_121690483 | Chr4 | 121690483 | 0.00035796 | 0.13 | 28.02 | HSW | E2 | GLM |
| Chr4_139205447 | Chr4 | 139205447 | 0.00045751 | 0.14 | 5.91 | HSW | BLUP | GLM |
| Chr4_338897743 | Chr4 | 338897743 | 0.00044194 | 0.46 | 14.44 | HSW | E2 | GLM |
| Chr4_447360607 | Chr4 | 447360607 | 0.00096165 | 0.07 | 16.25 | HSW | E1 | GLM |
| Chr5_11821350 | Chr5 | 11821350 | 0.00019826 | 0.23 | 8.08 | DF | E3 | GLM |
| Chr5_138162617 | Chr5 | 138162617 | 0.00003608 | 0.25 | 11.49 | HSW | E2 | GLM |
| Chr5_342836807 | Chr5 | 342836807 | 0.00015474 | 0.27 | 7.83 | DF | BLUP | GLM |
| Chr5_342836807 | Chr5 | 342836807 | 0.00018006 | 0.27 | 7.66 | DF | E1 | GLM |
| Chr5_342836807 | Chr5 | 342836807 | 0.00012955 | 0.27 | 21.35 | DF | E2 | GLM |
| Chr5_342836807 | Chr5 | 342836807 | 0.00077613 | 0.27 | 5.74 | DF | E3 | GLM |
| Chr5_413492767 | Chr5 | 413492767 | 0.00090486 | 0.39 | 2.31 | HSW | BLUP | GLM |
| Chr6_4338291 | Chr6 | 4338291 | 0.00030176 | 0.07 | 38.03 | DM | BLUP | GLM |
| Chr6_4338291 | Chr6 | 4338291 | 0.00028544 | 0.07 | 30.56 | DM | E3 | GLM |
| Chr6_128041250 | Chr6 | 128041250 | 0.00010600 | 0.23 | 29.14 | HSW | BLUP | GLM |
| Chr6_128041250 | Chr6 | 128041250 | 0.00033008 | 0.23 | 56.08 | HSW | E3 | GLM |
| Chr6_200603138 | Chr6 | 200603138 | 0.00001573 | 0.30 | 19.69 | DF | BLUP | GLM |
| Chr6_200603138 | Chr6 | 200603138 | 0.00001609 | 0.30 | 19.66 | DF | E1 | GLM |
| Chr6_200603138 | Chr6 | 200603138 | 0.00009794 | 0.30 | 13.97 | DF | E2 | GLM |
| Chr6_200603138 | Chr6 | 200603138 | 0.00003079 | 0.30 | 19.27 | DF | E3 | GLM |
| Chr6_200603138 | Chr6 | 200603138 | 0.00005734 | 0.30 | 14.97 | DM | BLUP | GLM |
| Chr6_200603138 | Chr6 | 200603138 | 0.00005237 | 0.30 | 16.84 | DM | E2 | GLM |
| Chr6_224444128 | Chr6 | 224444128 | 0.00040146 | 0.25 | 13.39 | PH | BLUP | GLM |
| Chr6_224444128 | Chr6 | 224444128 | 0.00069121 | 0.25 | 8.75 | PH | E1 | GLM |
| Chr6_253032265 | Chr6 | 253032265 | 0.00005436 | 0.15 | 12.29 | HSW | BLUP | GLM |
| Chr6_262648494 | Chr6 | 262648494 | 0.00062535 | 0.23 | 15.10 | YPP | BLUP | GLM |
| Chr6_262648494 | Chr6 | 262648494 | 0.00078069 | 0.23 | 14.55 | YPP | E2 | GLM |
| Chr6_262648494 | Chr6 | 262648494 | 0.00062739 | 0.23 | 15.10 | YPP | E1 | GLM |
| Chr6_294199001 | Chr6 | 294199001 | 0.00040781 | 0.11 | 13.67 | HSW | BLUP | GLM |
| Chr6_294199001 | Chr6 | 294199001 | 0.00040547 | 0.11 | 17.57 | HSW | E1 | GLM |
| Chr6_344313403 | Chr6 | 344313403 | 0.00032023 | 0.07 | 35.25 | PPP | BLUP | GLM |
| Chr6_344313403 | Chr6 | 344313403 | 0.00096084 | 0.07 | 31.53 | PPP | E2 | GLM |
| Chr7_9794560 | Chr7 | 9794560 | 0.00072273 | 0.17 | 6.15 | HSW | E1 | GLM |
| Chr7_42734754 | Chr7 | 42734754 | 0.00079844 | 0.41 | 18.77 | PH | E2 | GLM |
| Chr7_42734754 | Chr7 | 42734754 | 0.00102033 | 0.41 | 7.39 | DM | E3 | GLM |
| Chr7_295399575 | Chr7 | 295399575 | 0.00046659 | 0.09 | 16.88 | DF | BLUP | GLM |
| Chr7_295399575 | Chr7 | 295399575 | 0.00054872 | 0.09 | 16.63 | DF | E1 | GLM |
| Chr7_295399575 | Chr7 | 295399575 | 0.00032441 | 0.09 | 14.46 | DF | E3 | GLM |
| Chr7_297717438 | Chr7 | 297717438 | 0.00001779 | 0.25 | 16.72 | PH | E1 | GLM |

**Table S9**: Putative candidate genes associated with significant MTAs and their putative functions.

| **SNP** | **Trait** | **No. of genes** | **Gene ID** | **Chro.** | **Start** | **End** | **Description** |
| --- | --- | --- | --- | --- | --- | --- | --- |
| Chr1_72000142 | DM | 6 | *Lcu.2RBY.1g012000* | Chr1 | 71548828 | 71549832 | PTHR33095 family protein (protein-coding gene) |
|  |  |  | *Lcu.2RBY.1g012010* | Chr1 | 71552128 | 71554291 | PTHR11132:SF38 protein |
|  |  |  | *Lcu.2RBY.1g012020* | Chr1 | 71570415 | 71575310 | tRNA (adenine(58)-N(1))-methyltransferase non-catalytic subunit TRM6 |
|  |  |  | *Lcu.2RBY.1g012030* | Chr1 | 71575338 | 71576390 | Lysosomal Pro-Xaa carboxypeptidase / Prolyl carboxypeptidase |
|  |  |  | *Lcu.2RBY.1g012050* | Chr1 | 71875187 | 71876119 | Bactericidal permeability-increasing protein, alpha/beta domain |
|  |  |  | *Lcu.2RBY.1g012080* | Chr1 | 72397479 | 72398063 | GAG-pre-integrase domain-containing protein |
| Chr1_221418298 | PPP | 6 | *Lcu.2RBY.1g027320* | Chr1 | 220958165 | 220959100 | Ribosomal protein L13e; HAD-like domain-containing protein |
|  |  |  | *Lcu.2RBY.1g027330* | Chr1 | 220962679 | 220963609 | Aldehyde reductase / Polyol dehydrogenase (NADP⁺) |
|  |  |  | *Lcu.2RBY.1g027340* | Chr1 | 220980304 | 220980615 | Tetratricopeptide repeat protein 11 (TPR repeat protein) |
|  |  |  | *Lcu.2RBY.1g027350* | Chr1 | 221081964 | 221084175 | Replication factor A protein 1 (RFA1) |
|  |  |  | *Lcu.2RBY.1g027370* | Chr1 | 221283049 | 221288145 | F-box and leucine-rich repeat protein (FBXL2/20) |
|  |  |  | *Lcu.2RBY.1g027380* | Chr1 | 221301159 | 221305180 | Retrotransposon gag protein with aspartyl protease domain |
| Chr1_315275939 | HSW | 6 | *Lcu.2RBY.1g038210* | Chr1 | 314837684 | 314846344 | Xyloglucan endotransglucosylase/hydrolase protein 25–related |
|  |  |  | *Lcu.2RBY.1g038220* | Chr1 | 314846470 | 314847956 | Xyloglucan endotransglucosylase/hydrolase protein 25–related |
|  |  |  | *Lcu.2RBY.1g038230* | Chr1 | 314848466 | 314850049 | Ribosomal protein L18/L5; zinc finger (RING/FYVE/PHD-type) |
|  |  |  | *Lcu.2RBY.1g038240* | Chr1 | 314857505 | 314857888 | Protein serine/threonine phosphatase |
|  |  |  | *Lcu.2RBY.1g038290* | Chr1 | 315190729 | 315192639 | Transcription factor TCP1 |
|  |  |  | *Lcu.2RBY.1g038300* | Chr1 | 315729860 | 315734863 | Polyphosphate 5′-phosphatase–related protein |
| Chr1_396757210 | HSW | 4 | *Lcu.2RBY.1g051310* | Chr1 | 396341315 | 396343296 | Reverse transcriptase (RNA-dependent DNA polymerase); zinc knuckle; retrotransposon gag protein |
|  |  |  | *Lcu.2RBY.1g051330* | Chr1 | 396659039 | 396662415 | Gibberellin receptor GID1 |
|  |  |  | *Lcu.2RBY.1g051360* | Chr1 | 397136617 | 397137289 | Uncharacterized protein |
|  |  |  | *Lcu.2RBY.1g051370* | Chr1 | 397142928 | 397143901 | Uncharacterized protein |
| Chr1_482512926 | DM | 7 | *Lcu.2RBY.1g065700* | Chr1 | 482408754 | 482416514 | Cinnamyl-alcohol dehydrogenase (CAD) |
|  |  |  | *Lcu.2RBY.1g065710* | Chr1 | 482417755 | 482418554 | Cinnamyl-alcohol dehydrogenase (CAD) |
|  |  |  | *Lcu.2RBY.1g065730* | Chr1 | 482590639 | 482590938 | Uncharacterized protein |
|  |  |  | *Lcu.2RBY.1g065740* | Chr1 | 482611339 | 482617399 | Alpha/beta-hydrolase superfamily protein |
|  |  |  | *Lcu.2RBY.1g065770* | Chr1 | 482944295 | 482946652 | HR-like lesion-inducing protein |
|  |  |  | *Lcu.2RBY.1g065780* | Chr1 | 482968082 | 482970175 | Oxidoreductase, 2OG-Fe(II) oxygenase family protein |
|  |  |  | *Lcu.2RBY.1g065790* | Chr1 | 482970133 | 482975119 | Transcription factor EMB1444-related |
| Chr2_13220114 | DM | 23 | *Lcu.2RBY.2g006000.1* | Chr2 | 12799043 | 12802004 | Papain / Papaya peptidase I |
|  |  |  | *Lcu.2RBY.2g006010.1* | Chr2 | 12814983 | 12816908 | Papain / Papaya peptidase I |
|  |  |  | *Lcu.2RBY.2g006020.1* | Chr2 | 12833275 | 12834052 | Uncharacterized protein |
|  |  |  | *Lcu.2RBY.2g006030.1* | Chr2 | 12844674 | 12849508 | Uncharacterized hydrolase-like protein |
|  |  |  | *Lcu.2RBY.2g006040.1* | Chr2 | 12854976 | 12858040 | Multifunctional chaperone (14-3-3 family) |
|  |  |  | *Lcu.2RBY.2g006050.1* | Chr2 | 12870111 | 12871981 | F28N24.16 protein family |
|  |  |  | *Lcu.2RBY.2g006060.1* | Chr2 | 12902669 | 12904013 | Dof domain, zinc finger |
|  |  |  | *Lcu.2RBY.2g006070.1* | Chr2 | 12923886 | 12927940 | Altered xyloglucan/trichome birefringence-like |
|  |  |  | *Lcu.2RBY.2g006080.1* | Chr2 | 12935831 | 12936633 | Inositol transporter 4-related |
|  |  |  | *Lcu.2RBY.2g006090.1* | Chr2 | 12993540 | 12994434 | Uncharacterized protein |
|  |  |  | *Lcu.2RBY.2g006120.1* | Chr2 | 13072313 | 13078174 | Transcription factor GT-2, trihelix DNA-binding |
|  |  |  | *Lcu.2RBY.2g006150.1* | Chr2 | 13132561 | 13134107 | Domain of unknown function DUF4228 |
|  |  |  | *Lcu.2RBY.2g006160.1* | Chr2 | 13163449 | 13167694 | Peroxin-7 (PEX7) |
|  |  |  | *Lcu.2RBY.2g006170.1* | Chr2 | 13167700 | 13170611 | Cathepsin O |
|  |  |  | *Lcu.2RBY.2g006190.1* | Chr2 | 13200400 | 13204512 | F28N24.8 protein |
|  |  |  | *Lcu.2RBY.2g006200.1* | Chr2 | 13225560 | 13229046 | Cysteamine dioxygenase / Persulfurase |
|  |  |  | *Lcu.2RBY.2g006210.1* | Chr2 | 13242924 | 13247320 | LRR receptor-like serine/threonine-protein kinase |
|  |  |  | *Lcu.2RBY.2g006220.1* | Chr2 | 13250758 | 13251734 | CAMP-response element binding protein-related |
|  |  |  | *Lcu.2RBY.2g006230.1* | Chr2 | 13261617 | 13263684 | Serine/threonine-protein kinase, CBL-interacting 2 |
|  |  |  | *Lcu.2RBY.2g006240.1* | Chr2 | 13282576 | 13284701 | Serine/threonine-protein kinase, CBL-interacting 12 |
|  |  |  | *Lcu.2RBY.2g006250.1* | Chr2 | 13446023 | 13450428 | Leucine-rich repeat receptor-like protein kinase |
|  |  |  | *Lcu.2RBY.2g006260.1* | Chr2 | 13470461 | 13474739 | Xanthine-uracil / vitamin C permease family member |
|  |  |  | *Lcu.2RBY.2g006270.1* | Chr2 | 13474064 | 13477325 | O-fucosyltransferase-like protein |
| Chr2_159172425 | DF | 6 | *Lcu.2RBY.2g031110* | Chr2 | 159090867 | 159091887 | ZINC-FINGER HOMEODOMAIN PROTEIN 14 |
|  |  |  | *Lcu.2RBY.2g031130* | Chr2 | 159103946 | 159104344 | RNA helicase |
|  |  |  | *Lcu.2RBY.2g031150* | Chr2 | 159164766 | 159165239 | Ribonuclease H-like domain |
|  |  |  | *Lcu.2RBY.2g031160* | Chr2 | 159208255 | 159210281 | Uncharacterized protein |
|  |  |  | *Lcu.2RBY.2g031170* | Chr2 | 159231849 | 159232064 | Uncharacterized protein |
|  |  |  | *Lcu.2RBY.2g031180* | Chr2 | 159468269 | 159472530 | PROTEIN BASIC PENTACYSTEINE1-RELATED |
| Chr2_182480268 | DM | 17 | *Lcu.2RBY.2g033950* | Chr2 | 182000305 | 182003417 | Protein kinase domain; Serine/threonine/dual specificity protein kinase; Leucine-rich repeat-containing N-terminal domain |
|  |  |  | *Lcu.2RBY.2g033960* | Chr2 | 182133457 | 182138757 | Uncharacterized protein |
|  |  |  | *Lcu.2RBY.2g033980* | Chr2 | 182235084 | 182238368 | CYTOCHROME P450 SUPERFAMILY PROTEIN |
|  |  |  | *Lcu.2RBY.2g033990* | Chr2 | 182311016 | 182317338 | PROTEIN ARGONAUTE 5 |
|  |  |  | *Lcu.2RBY.2g034000* | Chr2 | 182341745 | 182345403 | Uncharacterized protein |
|  |  |  | *Lcu.2RBY.2g034020* | Chr2 | 182529035 | 182531574 | Leucine rich repeat N-terminal domain (LRRNT_2); Leucine rich repeat |
|  |  |  | *Lcu.2RBY.2g034030* | Chr2 | 182547212 | 182549955 | Pentatricopeptide repeat protein |
|  |  |  | *Lcu.2RBY.2g034040* | Chr2 | 182550502 | 182552121 | Adenylyl-sulfate reductase (glutathione); Plant-type 5'-adenylylsulfate reductase |
|  |  |  | *Lcu.2RBY.2g034050* | Chr2 | 182575367 | 182578045 | Uncharacterized conserved protein (KOG4529) |
|  |  |  | *Lcu.2RBY.2g034060* | Chr2 | 182652928 | 182659375 | ALCOHOL DEHYDROGENASE-LIKE 6 |
|  |  |  | *Lcu.2RBY.2g034070* | Chr2 | 182698989 | 182700860 | Uncharacterized protein |
|  |  |  | *Lcu.2RBY.2g034080* | Chr2 | 182701537 | 182702608 | Mannan endo-1,4-beta-mannosidase (Endo-1,4-mannanase) |
|  |  |  | *Lcu.2RBY.2g034090* | Chr2 | 182705962 | 182707625 | Complex 1 protein (LYR family) |
|  |  |  | *Lcu.2RBY.2g034100* | Chr2 | 182708620 | 182715925 | Uncharacterized protein |
|  |  |  | *Lcu.2RBY.2g034110* | Chr2 | 182849198 | 182849819 | Uncharacterized protein |
|  |  |  | *Lcu.2RBY.2g034130* | Chr2 | 182945166 | 182947069 | PEROXIDASE 20 |
|  |  |  | *Lcu.2RBY.2g034140* | Chr2 | 182952701 | 182954809 | Pentatricopeptide repeat (PPR) family protein |
| Chr2_410777988 | PPP | 22 | *Lcu.2RBY.2g064380* | Chr2 | 410296945 | 410303419 | Translation initiation factor eIF-2B subunit delta (EIF2B4) |
|  |  |  | *Lcu.2RBY.2g064390* | Chr2 | 410303476 | 410305308 | ADENINE NUCLEOTIDE ALPHA HYDROLASES-LIKE PROTEIN |
|  |  |  | *Lcu.2RBY.2g064400* | Chr2 | 410399483 | 410405875 | GALACTURONOSYLTRANSFERASE 10-RELATED |
|  |  |  | *Lcu.2RBY.2g064430* | Chr2 | 410521211 | 410522434 | ASPARTYL PROTEASE FAMILY PROTEIN |
|  |  |  | *Lcu.2RBY.2g064440* | Chr2 | 410578462 | 410585175 | ATP-dependent RNA helicase DDX18/HAS1 |
|  |  |  | *Lcu.2RBY.2g064450* | Chr2 | 410605728 | 410612528 | Karyopherin (importin) alpha |
|  |  |  | *Lcu.2RBY.2g064460* | Chr2 | 410654586 | 410659645 | Kinesin family member 18/19 (KIF18/19) |
|  |  |  | *Lcu.2RBY.2g064470* | Chr2 | 410663640 | 410665584 | Uncharacterized protein |
|  |  |  | *Lcu.2RBY.2g064480* | Chr2 | 410667933 | 410671718 | PROTEIN DR1 HOMOLOG |
|  |  |  | *Lcu.2RBY.2g064490* | Chr2 | 410704847 | 410710234 | Phospholipid-translocating ATPase (E3.6.3.1) |
|  |  |  | *Lcu.2RBY.2g064500* | Chr2 | 410752181 | 410757722 | Uncharacterized protein |
|  |  |  | *Lcu.2RBY.2g064510* | Chr2 | 410757712 | 410757921 | L-ascorbate oxidase (Ascorbase) |
|  |  |  | *Lcu.2RBY.2g064520* | Chr2 | 410866594 | 410867114 | Uncharacterized protein |
|  |  |  | *Lcu.2RBY.2g064530* | Chr2 | 410870435 | 410879425 | Acetate—CoA ligase / Acyl-activating enzyme |
|  |  |  | *Lcu.2RBY.2g064540* | Chr2 | 410883747 | 410884941 | Tetraspanin family protein |
|  |  |  | *Lcu.2RBY.2g064550* | Chr2 | 410910237 | 410910610 | Uncharacterized protein |
|  |  |  | *Lcu.2RBY.2g064560* | Chr2 | 410945703 | 410946161 | Uncharacterized protein |
|  |  |  | *Lcu.2RBY.2g064580* | Chr2 | 410977244 | 411003814 | Uncharacterized protein |
|  |  |  | *Lcu.2RBY.2g064600* | Chr2 | 411086023 | 411086700 | Uncharacterized protein |
|  |  |  | *Lcu.2RBY.2g064610* | Chr2 | 411088431 | 411092664 | ENOLASE 1, chloroplastic |
|  |  |  | *Lcu.2RBY.2g064620* | Chr2 | 411097441 | 411103121 | Cysteamine dioxygenase (Persulfurase) |
|  |  |  | *Lcu.2RBY.2g064640* | Chr2 | 411209633 | 411210196 | DNA-binding pseudobarrel domain protein (LcE1) |
| Chr2_484037996 | HSW | 11 | *Lcu.2RBY.2g074550* | Chr2 | 483717503 | 483728345 | DNA topoisomerase 2-associated protein PAT1 (PATL1, PAT1) |
|  |  |  | *Lcu.2RBY.2g074560* | Chr2 | 483929684 | 483937410 | DNA topoisomerase 2-associated protein PAT1 (PATL1, PAT1) |
|  |  |  | *Lcu.2RBY.2g074570* | Chr2 | 483963979 | 483972111 | BTB/POZ domain protein; Armadillo-type fold |
|  |  |  | *Lcu.2RBY.2g074580* | Chr2 | 483978706 | 483979191 | Uncharacterized protein |
|  |  |  | *Lcu.2RBY.2g074590* | Chr2 | 483983518 | 483984501 | Thaumatin family protein |
|  |  |  | *Lcu.2RBY.2g074600* | Chr2 | 484023586 | 484024014 | PHD finger protein / Histone-lysine N-methyltransferase ATX1-related |
|  |  |  | *Lcu.2RBY.2g074610* | Chr2 | 484040951 | 484043968 | Pentatricopeptide repeat / Translation initiation factor 2 alpha subunit |
|  |  |  | *Lcu.2RBY.2g074630* | Chr2 | 484131171 | 484132531 | Uncharacterized protein |
|  |  |  | *Lcu.2RBY.2g074640* | Chr2 | 484379226 | 484379609 | Serine/threonine-specific protein phosphatase |
|  |  |  | *Lcu.2RBY.2g074650* | Chr2 | 484380536 | 484386192 | Snapin/Pallidin family protein |
|  |  |  | *Lcu.2RBY.2g074660* | Chr2 | 484486195 | 484488645 | Uncharacterized protein |
| Chr2_521877344 | DF | 9 | *Lcu.2RBY.2g079030* | Chr2 | 521457446 | 521460346 | Uncharacterized protein |
|  |  |  | *Lcu.2RBY.2g079040* | Chr2 | 521605407 | 521606720 | ANTIPORTER / DRUG TRANSPORTER-RELATED |
|  |  |  | *Lcu.2RBY.2g079050* | Chr2 | 521696866 | 521699676 | Uncharacterized protein |
|  |  |  | *Lcu.2RBY.2g079060* | Chr2 | 521725216 | 521727829 | Uncharacterized protein |
|  |  |  | *Lcu.2RBY.2g079070* | Chr2 | 521781048 | 521788933 | tRNA dimethylallyltransferase / tRNA prenyltransferase |
|  |  |  | *Lcu.2RBY.2g079080* | Chr2 | 521782629 | 521783315 | Uncharacterized protein |
|  |  |  | *Lcu.2RBY.2g079110* | Chr2 | 521913106 | 521914525 | TPR (Tetratricopeptide repeat) protein |
|  |  |  | *Lcu.2RBY.2g079120* | Chr2 | 521923404 | 521924108 | Zinc-binding reverse transcriptase (zf-RVT) domain protein |
|  |  |  | *Lcu.2RBY.2g079130* | Chr2 | 522094021 | 522094458 | GAG/POL/ENV POLYPROTEIN subfamily protein |
| Chr2_603974724 | HSW | 31 | *Lcu.2RBY.2g094020* | Chr2 | 603482982 | 603486449 | 5′-nucleotidase SURE |
|  |  |  | *Lcu.2RBY.2g094030* | Chr2 | 603518590 | 603525366 | Serine/threonine-protein phosphatase 5 (PPP5C) |
|  |  |  | *Lcu.2RBY.2g094040* | Chr2 | 603559796 | 603567538 | Phospholipid-transporting ATPase TAT-1 family protein |
|  |  |  | *Lcu.2RBY.2g094070* | Chr2 | 603607408 | 603608036 | Uncharacterized protein |
|  |  |  | *Lcu.2RBY.2g094080* | Chr2 | 603615294 | 603621031 | Cwf19-like protein with HIT-like domain |
|  |  |  | *Lcu.2RBY.2g094090* | Chr2 | 603636161 | 603641258 | Pentatricopeptide repeat-containing protein (PPR) |
|  |  |  | *Lcu.2RBY.2g094100* | Chr2 | 603667584 | 603669010 | Uncharacterized protein |
|  |  |  | *Lcu.2RBY.2g094110* | Chr2 | 603692880 | 603694193 | 40S ribosomal protein S14 (RPS14) |
|  |  |  | *Lcu.2RBY.2g094120* | Chr2 | 603711757 | 603717051 | Casein kinase 1 (CSNK1/CK1) |
|  |  |  | *Lcu.2RBY.2g094130* | Chr2 | 603746200 | 603746814 | Domain of unknown function (DUF3511) |
|  |  |  | *Lcu.2RBY.2g094140* | Chr2 | 603818749 | 603822120 | Translation initiation factor 4A (EIF4A) |
|  |  |  | *Lcu.2RBY.2g094160* | Chr2 | 603836757 | 603838253 | F-box domain protein |
|  |  |  | *Lcu.2RBY.2g094170* | Chr2 | 603847822 | 603849030 | Pre-mRNA splicing factor–related protein |
|  |  |  | *Lcu.2RBY.2g094180* | Chr2 | 603850816 | 603857335 | Telomere repeat-binding factor 4–related protein |
|  |  |  | *Lcu.2RBY.2g094190* | Chr2 | 603966757 | 603970096 | Telomere repeat-binding factor 4–related protein |
|  |  |  | *Lcu.2RBY.2g094210* | Chr2 | 604063990 | 604067631 | Linoleate 9S-lipoxygenase (LOX1_5) |
|  |  |  | *Lcu.2RBY.2g094220* | Chr2 | 604069306 | 604073030 | Linoleate 9S-lipoxygenase (LOX1_5) |
|  |  |  | *Lcu.2RBY.2g094230* | Chr2 | 604144023 | 604147332 | Mitochondrial import inner membrane translocase subunit TIM23 |
|  |  |  | *Lcu.2RBY.2g094240* | Chr2 | 604152956 | 604155362 | Uncharacterized protein |
|  |  |  | *Lcu.2RBY.2g094250* | Chr2 | 604159415 | 604161739 | 2-haloacrylate reductase |
|  |  |  | *Lcu.2RBY.2g094260* | Chr2 | 604207471 | 604210164 | Protein kinase with adenine nucleotide α-hydrolase–like domain |
|  |  |  | *Lcu.2RBY.2g094270* | Chr2 | 604218884 | 604220364 | Uncharacterized protein |
|  |  |  | *Lcu.2RBY.2g094280* | Chr2 | 604218891 | 604219220 | Uncharacterized protein |
|  |  |  | *Lcu.2RBY.2g094290* | Chr2 | 604221382 | 604223367 | Threonine synthase (ThrC) |
|  |  |  | *Lcu.2RBY.2g094300* | Chr2 | 604234039 | 604235505 | Peptide α-N-acetyltransferase |
|  |  |  | *Lcu.2RBY.2g094310* | Chr2 | 604237145 | 604241616 | PHD finger protein ALFIN-like 6–related |
|  |  |  | *Lcu.2RBY.2g094320* | Chr2 | 604363167 | 604370767 | Ataxin-2 SM domain protein (SM-ATX) |
|  |  |  | *Lcu.2RBY.2g094330* | Chr2 | 604372479 | 604375865 | Nuclear transcription factor Y subunit A-3–related |
|  |  |  | *Lcu.2RBY.2g094340* | Chr2 | 604385554 | 604386500 | Stress-induced protein kinase 1 (KIN1)-related |
|  |  |  | *Lcu.2RBY.2g094350* | Chr2 | 604395182 | 604399250 | DNA-directed RNA polymerase III subunit RPC6 |
|  |  |  | *Lcu.2RBY.2g094360* | Chr2 | 604432252 | 604432985 | Uncharacterized protein |
| CHR3_90640061 | HSW | 9 | *Lcu.2RBY.3g014860* | Chr3 | 90151318 | 90154352 | Autophagy-related protein 8A–related |
|  |  |  | *Lcu.2RBY.3g014870* | Chr3 | 90156094 | 90157427 | SQUAMOSA promoter-binding-like protein 10–related |
|  |  |  | *Lcu.2RBY.3g014880* | Chr3 | 90266555 | 90267100 | GH08677P family protein |
|  |  |  | *Lcu.2RBY.3g014890* | Chr3 | 90292972 | 90293409 | Gag-polypeptide of LTR copia-type / DUF4219 domain protein |
|  |  |  | *Lcu.2RBY.3g014900* | Chr3 | 90587242 | 90589438 | Sulfate transporter 1.1–related |
|  |  |  | *Lcu.2RBY.3g014920* | Chr3 | 90594947 | 90597114 | Sulfate transporter 1.1–related |
|  |  |  | *Lcu.2RBY.3g014930* | Chr3 | 90597499 | 90598407 | Sulfate transporter 1.1–related |
|  |  |  | *Lcu.2RBY.3g014940* | Chr3 | 90818521 | 90819546 | Heat shock protein 90 / Endoplasmin homolog |
|  |  |  | *Lcu.2RBY.3g014950* | Chr3 | 90868878 | 90870129 | ZF-HD dimerization region protein (ZF-HD_dimer domain) |
| Chr3_106842007 | DF | 13 | *Lcu.2RBY.3g016910* | Chr3 | 106509395 | 106510053 | MADS-box protein family transcription factor |
|  |  |  | *Lcu.2RBY.3g016920* | Chr3 | 106576168 | 106583485 | Protein SMG7 (EST1C), involved in mRNA decay |
|  |  |  | *Lcu.2RBY.3g016930* | Chr3 | 106647368 | 106654631 | Non-specific phospholipase C2 |
|  |  |  | *Lcu.2RBY.3g016940* | Chr3 | 106652641 | 106654584 | ABC transporter G family member 23 |
|  |  |  | *Lcu.2RBY.3g016960* | Chr3 | 106791216 | 106793970 | Ferric reductase-like protein |
|  |  |  | *Lcu.2RBY.3g016970* | Chr3 | 106795574 | 106795993 | Zinc finger CCCH domain–containing protein 66 |
|  |  |  | *Lcu.2RBY.3g016980* | Chr3 | 106796713 | 106797928 | Zinc finger CCCH domain–containing protein 66 |
|  |  |  | *Lcu.2RBY.3g016990* | Chr3 | 106831691 | 106832550 | Uncharacterized protein |
|  |  |  | *Lcu.2RBY.3g017000* | Chr3 | 106903870 | 106904409 | Uncharacterized protein |
|  |  |  | *Lcu.2RBY.3g017010* | Chr3 | 106910547 | 106912458 | Uncharacterized protein |
|  |  |  | *Lcu.2RBY.3g017020* | Chr3 | 107071951 | 107074711 | Phospholipase A2 domain–containing protein |
|  |  |  | *Lcu.2RBY.3g017030* | Chr3 | 107090700 | 107097816 | E3 ubiquitin-protein ligase RBBP6 |
|  |  |  | *Lcu.2RBY.3g017050* | Chr3 | 107219318 | 107219672 | Serine carboxypeptidase (S10 family) |
| Chr3_126687055 | PPP | 4 | *Lcu.2RBY.3g019430* | Chr3 | 126256234 | 126257292 | 60S ribosomal protein L35 |
|  |  |  | *Lcu.2RBY.3g019470* | Chr3 | 126886314 | 126891072 | Uncharacterized protein |
|  |  |  | *Lcu.2RBY.3g019480* | Chr3 | 127055475 | 127063741 | Serine/threonine protein phosphatase |
|  |  |  | *Lcu.2RBY.3g019490* | Chr3 | 127106859 | 127109036 | Leucine-rich repeat (LRR) protein |
| Chr3_163495081 | PPP | 1 | *Lcu.2RBY.3g024650* | Chr3 | 163870393 | 163880359 | Protein DEK |
| Chr3_181072527 | PH | 13 | *Lcu.2RBY.3g026740* | Chr3 | 180634978 | 180638799 | Oligopeptide transporter-related protein |
|  |  |  | *Lcu.2RBY.3g026750* | Chr3 | 180740363 | 180743527 | Uncharacterized protein |
|  |  |  | *Lcu.2RBY.3g026760* | Chr3 | 180751131 | 180754458 | Multicopper oxidase / laccase-16 |
|  |  |  | *Lcu.2RBY.3g026790* | Chr3 | 181027532 | 181030132 | DNAJ (HSP40) homolog protein |
|  |  |  | *Lcu.2RBY.3g026810* | Chr3 | 181214966 | 181220140 | Beta-D-glucan exohydrolase-like protein |
|  |  |  | *Lcu.2RBY.3g026830* | Chr3 | 181307404 | 181312323 | Mitochondrial Fe/S cluster exporter (ABC transporter) |
|  |  |  | *Lcu.2RBY.3g026850* | Chr3 | 181335203 | 181338790 | Uncharacterized protein |
|  |  |  | *Lcu.2RBY.3g026860* | Chr3 | 181376478 | 181378295 | MYB-like DNA-binding protein |
|  |  |  | *Lcu.2RBY.3g026880* | Chr3 | 181394344 | 181401426 | Peptidase M41 / AAA domain-containing protein |
|  |  |  | *Lcu.2RBY.3g026890* | Chr3 | 181547454 | 181550957 | Phosphomethylpyrimidine synthase |
|  |  |  | *Lcu.2RBY.3g026900* | Chr3 | 181553343 | 181553735 | Uncharacterized protein |
|  |  |  | *Lcu.2RBY.3g026910* | Chr3 | 181554298 | 181555525 | Uncharacterized protein |
|  |  |  | *Lcu.2RBY.3g026920* | Chr3 | 181554841 | 181555125 | Thylakoid soluble phosphoprotein TSP9 |
| Chr3_184872087 | DF | 1 | *Lcu.2RBY.3g027230* | Chr3 | 184536241 | 184537125 | Negative regulator of vesicle formation–related protein |
| Chr3_274815239 | DM | 14 | *Lcu.2RBY.3g041560* | Chr3 | 274324400 | 274325073 | Uncharacterized protein |
|  |  |  | *Lcu.2RBY.3g041570* | Chr3 | 274360405 | 274361133 | Zinc knuckle (CCHC) domain-containing protein |
|  |  |  | *Lcu.2RBY.3g041580* | Chr3 | 274388079 | 274402385 | MORC family ATPase |
|  |  |  | *Lcu.2RBY.3g041610* | Chr3 | 274497285 | 274497590 | RNA recognition motif (RRM) protein |
|  |  |  | *Lcu.2RBY.3g041620* | Chr3 | 274519475 | 274521866 | Polygalacturonase / pectinase |
|  |  |  | *Lcu.2RBY.3g041630* | Chr3 | 274576098 | 274581611 | Uncharacterized protein |
|  |  |  | *Lcu.2RBY.3g041640* | Chr3 | 274589492 | 274591486 | Glutaredoxin-C1 |
|  |  |  | *Lcu.2RBY.3g041660* | Chr3 | 274680356 | 274685745 | Anthranilate phosphoribosyltransferase (TrpD) |
|  |  |  | *Lcu.2RBY.3g041670* | Chr3 | 274762415 | 274768807 | PX domain / zinc-RING-containing protein |
|  |  |  | *Lcu.2RBY.3g041680* | Chr3 | 274768848 | 274770992 | Uncharacterized protein |
|  |  |  | *Lcu.2RBY.3g041690* | Chr3 | 274815005 | 274818583 | Solute carrier family 35 protein |
|  |  |  | *Lcu.2RBY.3g041700* | Chr3 | 274896698 | 274898256 | MYB-like transcription factor MYB98 |
|  |  |  | *Lcu.2RBY.3g041710* | Chr3 | 275019521 | 275024490 | Anion exchange protein |
|  |  |  | *Lcu.2RBY.3g041720* | Chr3 | 275174152 | 275176541 | Iron–sulfur cluster assembly protein NIFU |
| Chr4_121690483 | HSW | 2 | *Lcu.2RBY.4g019970* | Chr4 | 121207805 | 121220085 | Conserved oligomeric Golgi complex subunit 7 |
|  |  |  | *Lcu.2RBY.4g020000* | Chr4 | 121737706 | 121740959 | Glycerol-3-phosphate-transporting ATPase |
| Chr4_128357096 | HSW | 6 | *Lcu.2RBY.4g020600* | Chr4 | 127866432 | 127867993 | Expansin A20 |
|  |  |  | *Lcu.2RBY.4g020610* | Chr4 | 127871915 | 127893166 | Sel1-like repeat / tetratricopeptide-like protein |
|  |  |  | *Lcu.2RBY.4g020620* | Chr4 | 128110514 | 128110960 | Vesicle-fusing ATPase |
|  |  |  | *Lcu.2RBY.4g020630* | Chr4 | 128111509 | 128112569 | Aldehyde oxidase |
|  |  |  | *Lcu.2RBY.4g020640* | Chr4 | 128380482 | 128382960 | Expansin B1-related protein |
|  |  |  | *Lcu.2RBY.4g020670* | Chr4 | 128536312 | 128537852 | Acyl-CoA N-acyltransferase–like protein |
| Chr4_139205447 | HSW | 3 | *Lcu.2RBY.4g021620* | Chr4 | 139288480 | 139295494 | Acid phosphatase–related / metallophosphoesterase domain-containing protein |
|  |  |  | *Lcu.2RBY.4g021640* | Chr4 | 139580554 | 139589479 | Acid phosphatase–related / metallophosphoesterase domain-containing protein |
|  |  |  | *Lcu.2RBY.4g021650* | Chr4 | 139696636 | 139697031 | Protein phosphatase 2C (PP2C)-related protein |
| Chr4_338897743 | HSW | 21 | *Lcu.2RBY.4g050560* | Chr4 | 338609709 | 338610448 | Uncharacterized protein |
|  |  |  | *Lcu.2RBY.4g050570* | Chr4 | 338642920 | 338643315 | RNA-directed DNA polymerase / reverse transcriptase / RNase H |
|  |  |  | *Lcu.2RBY.4g050580* | Chr4 | 338665958 | 338672427 | Uncharacterized conserved protein |
|  |  |  | *Lcu.2RBY.4g050600* | Chr4 | 338749294 | 338753912 | Ras GTPase-activating protein–binding / nuclear transport factor 2–RRM protein |
|  |  |  | *Lcu.2RBY.4g050610* | Chr4 | 338799366 | 338807391 | Asparagine synthase |
|  |  |  | *Lcu.2RBY.4g050620* | Chr4 | 338887966 | 338890218 | L-ascorbate oxidase |
|  |  |  | *Lcu.2RBY.4g050640* | Chr4 | 338983515 | 338986770 | Multicopper oxidase (Cu-oxidase family) |
|  |  |  | *Lcu.2RBY.4g050650* | Chr4 | 339004015 | 339006459 | Duplicated SANT DNA-binding domain protein |
|  |  |  | *Lcu.2RBY.4g050660* | Chr4 | 339007537 | 339008300 | WAT1-related protein |
|  |  |  | *Lcu.2RBY.4g050670* | Chr4 | 339044964 | 339049482 | PUR family transcriptional regulator |
|  |  |  | *Lcu.2RBY.4g050680* | Chr4 | 339055716 | 339062131 | Uncharacterized protein |
|  |  |  | *Lcu.2RBY.4g050690* | Chr4 | 339073400 | 339077889 | Uncharacterized protein |
|  |  |  | *Lcu.2RBY.4g050700* | Chr4 | 339078312 | 339082086 | Pyruvate kinase–related protein |
|  |  |  | *Lcu.2RBY.4g050710* | Chr4 | 339155095 | 339158002 | Pyruvate kinase |
|  |  |  | *Lcu.2RBY.4g050720* | Chr4 | 339164762 | 339167787 | Adenylyl-sulfate reductase (thioredoxin-dependent) |
|  |  |  | *Lcu.2RBY.4g050730* | Chr4 | 339190707 | 339191406 | Protein phosphatase 2C-related protein |
|  |  |  | *Lcu.2RBY.4g050740* | Chr4 | 339191442 | 339192056 | Mediator of RNA polymerase II transcription subunit 37E-related |
|  |  |  | *Lcu.2RBY.4g050750* | Chr4 | 339252978 | 339253928 | Uncharacterized protein |
|  |  |  | *Lcu.2RBY.4g050770* | Chr4 | 339278245 | 339278553 | Uncharacterized protein |
|  |  |  | *Lcu.2RBY.4g050780* | Chr4 | 339326971 | 339338300 | Glucosidase II subunit beta |
|  |  |  | *Lcu.2RBY.4g050790* | Chr4 | 339353920 | 339357878 | Antitermination NusB domain–containing protein |
| Chr4_348520593 | DF | 11 | *Lcu.2RBY.4g052390* | Chr4 | 348029570 | 348038065 | Abscisate beta-glucosyltransferase (ABA-GTase) |
|  |  |  | *Lcu.2RBY.4g052400* | Chr4 | 348031102 | 348031437 | Uncharacterized protein |
|  |  |  | *Lcu.2RBY.4g052410* | Chr4 | 348087041 | 348091690 | Inosine-5-monophosphate dehydrogenase-related |
|  |  |  | *Lcu.2RBY.4g052430* | Chr4 | 348217316 | 348218341 | Plant mobile domain-containing protein |
|  |  |  | *Lcu.2RBY.4g052440* | Chr4 | 348330848 | 348331492 | RNA-directed DNA polymerase (retrotransposon-related) |
|  |  |  | *Lcu.2RBY.4g052460* | Chr4 | 348482188 | 348488214 | Polyadenylate-binding protein |
|  |  |  | *Lcu.2RBY.4g052470* | Chr4 | 348521850 | 348529160 | E3 ubiquitin-protein ligase MARCH6 |
|  |  |  | *Lcu.2RBY.4g052480* | Chr4 | 348703920 | 348709130 | Filament-like plant protein |
|  |  |  | *Lcu.2RBY.4g052490* | Chr4 | 348709149 | 348710144 | Ribosomal protein S16e |
|  |  |  | *Lcu.2RBY.4g052500* | Chr4 | 348732888 | 348734683 | Caffeoyl-CoA O-methyltransferase |
|  |  |  | *Lcu.2RBY.4g052530* | Chr4 | 348917134 | 348927437 | RING finger domain-containing protein |
| Chr4_447360607 | HSW | 21 | *Lcu.2RBY.4g073070* | Chr4 | 447013912 | 447019855 | Lipoxygenase 6, chloroplastic |
|  |  |  | *Lcu.2RBY.4g073080* | Chr4 | 447038767 | 447042717 | Uncharacterized protein |
|  |  |  | *Lcu.2RBY.4g073090* | Chr4 | 447043567 | 447050562 | Cell division cycle 2-like protein (CDC2L) |
|  |  |  | *Lcu.2RBY.4g073100* | Chr4 | 447108065 | 447110369 | Protein of unknown family (F12A21.28-related) |
|  |  |  | *Lcu.2RBY.4g073110* | Chr4 | 447127238 | 447135272 | ATP-dependent DNA helicase Q4 (RECQL4) |
|  |  |  | *Lcu.2RBY.4g073130* | Chr4 | 447169057 | 447173418 | Polynucleotide 5′-hydroxyl kinase |
|  |  |  | *Lcu.2RBY.4g073140* | Chr4 | 447187416 | 447189722 | Amino acid transporter |
|  |  |  | *Lcu.2RBY.4g073150* | Chr4 | 447192183 | 447195424 | Ankyrin repeat and protein kinase domain-containing protein |
|  |  |  | *Lcu.2RBY.4g073180* | Chr4 | 447295455 | 447295893 | NADH-ubiquinone reductase complex I subunit (MLRQ) |
|  |  |  | *Lcu.2RBY.4g073190* | Chr4 | 447311308 | 447317654 | Xenobiotic/steroid-transporting ATPase |
|  |  |  | *Lcu.2RBY.4g073200* | Chr4 | 447349875 | 447351148 | DUF4283 domain-containing protein |
|  |  |  | *Lcu.2RBY.4g073210* | Chr4 | 447356017 | 447358277 | Putative phage-type endonuclease |
|  |  |  | *Lcu.2RBY.4g073220* | Chr4 | 447412386 | 447417896 | F-box domain-containing protein |
|  |  |  | *Lcu.2RBY.4g073230* | Chr4 | 447539398 | 447543851 | Signal recognition particle subunit SRP72 |
|  |  |  | *Lcu.2RBY.4g073240* | Chr4 | 447578403 | 447580142 | Salutaridinol 7-O-acetyltransferase |
|  |  |  | *Lcu.2RBY.4g073260* | Chr4 | 447669139 | 447670514 | Vinorine synthase |
|  |  |  | *Lcu.2RBY.4g073280* | Chr4 | 447721621 | 447722620 | Uncharacterized protein |
|  |  |  | *Lcu.2RBY.4g073290* | Chr4 | 447771807 | 447774754 | RING finger protein 41-like |
|  |  |  | *Lcu.2RBY.4g073300* | Chr4 | 447830525 | 447831301 | F23H11.23 protein-related |
|  |  |  | *Lcu.2RBY.4g073310* | Chr4 | 447842852 | 447843438 | 25S rRNA (adenine-N1)-methyltransferase |
|  |  |  | *Lcu.2RBY.4g073320* | Chr4 | 447843497 | 447843789 | Ribosomal RNA-processing protein 8 |
| Chr4_466290148 | HSW | 24 | *Lcu.2RBY.4g076930* | Chr4 | 465828014 | 465829053 | Uncharacterized protein |
|  |  |  | *Lcu.2RBY.4g076940* | Chr4 | 465832071 | 465835322 | DUF3593 protein |
|  |  |  | *Lcu.2RBY.4g076950* | Chr4 | 465832071 | 465835322 | WRKY transcription factor |
|  |  |  | *Lcu.2RBY.4g076960* | Chr4 | 465849563 | 465850768 | Uncharacterized protein |
|  |  |  | *Lcu.2RBY.4g076970* | Chr4 | 465866151 | 465867320 | Uncharacterized protein |
|  |  |  | *Lcu.2RBY.4g076980* | Chr4 | 465895458 | 465896586 | Uncharacterized protein |
|  |  |  | *Lcu.2RBY.4g076990* | Chr4 | 465895511 | 465895915 | Uncharacterized protein |
|  |  |  | *Lcu.2RBY.4g077000* | Chr4 | 465898081 | 465901792 | Uncharacterized protein |
|  |  |  | *Lcu.2RBY.4g077010* | Chr4 | 465898081 | 465900073 | Uncharacterized protein |
|  |  |  | *Lcu.2RBY.4g077020* | Chr4 | 465939837 | 465940550 | Proteinase inhibitor (S8/I9) |
|  |  |  | *Lcu.2RBY.4g077060* | Chr4 | 466345296 | 466345742 | Reverse transcriptase / RNase H |
|  |  |  | *Lcu.2RBY.4g077080* | Chr4 | 466407892 | 466413192 | Endo-1,4-β-glucanase |
|  |  |  | *Lcu.2RBY.4g077100* | Chr4 | 466470320 | 466472232 | Ribosomal protein L27e |
|  |  |  | *Lcu.2RBY.4g077110* | Chr4 | 466472813 | 466477353 | LRR cysteine-containing protein |
|  |  |  | *Lcu.2RBY.4g077120* | Chr4 | 466488166 | 466493750 | Uncharacterized protein |
|  |  |  | *Lcu.2RBY.4g077130* | Chr4 | 466492782 | 466500596 | Phosphatidylinositol synthase / LCFA-CoA ligase |
|  |  |  | *Lcu.2RBY.4g077140* | Chr4 | 466516191 | 466520169 | FYTT (Forty-two-three protein) |
|  |  |  | *Lcu.2RBY.4g077170* | Chr4 | 466529965 | 466533047 | Nucleoside diphosphate kinase-related |
|  |  |  | *Lcu.2RBY.4g077180* | Chr4 | 466542108 | 466545837 | Uncharacterized protein |
|  |  |  | *Lcu.2RBY.4g077190* | Chr4 | 466552519 | 466556043 | Uncharacterized protein |
|  |  |  | *Lcu.2RBY.4g077200* | Chr4 | 466562607 | 466563034 | Uncharacterized protein |
|  |  |  | *Lcu.2RBY.4g077230* | Chr4 | 466754214 | 466757094 | PPR / DYW deaminase protein |
|  |  |  | *Lcu.2RBY.4g077240* | Chr4 | 466757164 | 466757493 | Uncharacterized protein |
|  |  |  | *Lcu.2RBY.4g077250* | Chr4 | 466766204 | 466769215 | DnaJ homolog subfamily C member 2 |
| Chr5_11821350 | DF | 35 | *Lcu.2RBY.5g006720* | Chr5 | 11313689 | 11326216 | DNA polymerase alpha subunit A (POLA1) |
|  |  |  | *Lcu.2RBY.5g006730* | Chr5 | 11419485 | 11422580 | Protein kinase domain /Serine-threonine/tyrosine-protein kinase catalytic domain / Serine/threonine/dual specificity protein kinase |
|  |  |  | *Lcu.2RBY.5g006740* | Chr5 | 11430519 | 11434213 | F14J22.5 PROTEIN-RELATED |
|  |  |  | *Lcu.2RBY.5g006750* | Chr5 | 11441432 | 11442726 | Uncharacterized protein |
|  |  |  | *Lcu.2RBY.5g006770* | Chr5 | 11446554 | 11448438 | MITOGEN-ACTIVATED KINASE KINASE KINASE /MITOGEN-ACTIVATED PROTEIN KINASE KINASE KINASE |
|  |  |  | *Lcu.2RBY.5g006780* | Chr5 | 11454059 | 11455145 | PROTEIN RALF-LIKE 26-RELATED |
|  |  |  | *Lcu.2RBY.5g006790* | Chr5 | 11488973 | 11494493 | MITOGEN-ACTIVATED PROTEIN KINASE KINASE KINASE 19-RELATED |
|  |  |  | *Lcu.2RBY.5g006800* | Chr5 | 11513204 | 11513581 | MITOGEN-ACTIVATED PROTEIN KINASE KINASE KINASE 19-RELATED |
|  |  |  | *Lcu.2RBY.5g006810* | Chr5 | 11513697 | 11514370 | MITOGEN-ACTIVATED PROTEIN KINASE KINASE KINASE 19-RELATED |
|  |  |  | *Lcu.2RBY.5g006820* | Chr5 | 11549346 | 11550675 | TRANSCRIPTION FACTOR HEC1 |
|  |  |  | *Lcu.2RBY.5g006870* | Chr5 | 11624124 | 11627356 | PTHR21493//PTHR21493:SF142 - CGI-141-RELATED/LIPASE CONTAINING PROTEIN |
|  |  |  | *Lcu.2RBY.5g006880* | Chr5 | 11646660 | 11649866 | LIPASE CONTAINING PROTEIN |
|  |  |  | *Lcu.2RBY.5g006890* | Chr5 | 11656432 | 11660832 | RELATED/LIPASE CONTAINING PROTEIN |
|  |  |  | *Lcu.2RBY.5g006900* | Chr5 | 11703066 | 11709245 | MSI2 PROTEIN-RELATED |
|  |  |  | *Lcu.2RBY.5g006910* | Chr5 | 11709106 | 11711394 | Remorin-terminal region (Remorin_C) |
|  |  |  | *Lcu.2RBY.5g006920* | Chr5 | 11718587 | 11719274 | Uncharacterized protein |
|  |  |  | *Lcu.2RBY.5g006930* | Chr5 | 11784138 | 11789428 | zeaxanthin epoxidase |
|  |  |  | *Lcu.2RBY.5g006940* | Chr5 | 11785799 | 11786131 | Uncharacterized protein |
|  |  |  | *Lcu.2RBY.5g006950* | Chr5 | 11849180 | 11849719 | Uncharacterized protein |
|  |  |  | *Lcu.2RBY.5g006960* | Chr5 | 11849784 | 11850374 | Uncharacterized protein |
|  |  |  | *Lcu.2RBY.5g006970* | Chr5 | 11898795 | 11901926 | Uncharacterized protein |
|  |  |  | *Lcu.2RBY.5g006980* | Chr5 | 11902564 | 11904949 | Domain of unknown function (DUF3411) |
|  |  |  | *Lcu.2RBY.5g006990* | Chr5 | 11904480 | 11910960 | Arginine/serine-rich 16 (SFRS16) |
|  |  |  | *Lcu.2RBY.5g007000* | Chr5 | 11928608 | 11930495 | Uncharacterized protein |
|  |  |  | *Lcu.2RBY.5g007010* | Chr5 | 11997317 | 12000244 | HEAT SHOCK TRANSCRIPTION FACTOR // HEAT STRESS TRANSCRIPTION FACTOR B-1 |
|  |  |  | *Lcu.2RBY.5g007020* | Chr5 | 12000695 | 12002706 | Uncharacterized protein |
|  |  |  | *Lcu.2RBY.5g007030* | Chr5 | 12029116 | 12032339 | Uncharacterized protein |
|  |  |  | *Lcu.2RBY.5g007040* | Chr5 | 12053327 | 12055111 | Uncharacterized protein |
|  |  |  | *Lcu.2RBY.5g007060* | Chr5 | 12069004 | 12070137 | EF-HAND CALCIUM-BINDING DOMAIN CONTAINING PROTEIN // CALCIUM-BINDING PROTEIN CML24-RELATED |
|  |  |  | *Lcu.2RBY.5g007090* | Chr5 | 12129351 | 12130494 | PTHR31722//PTHR31722:SF5 - FAMILY NOT NAMED |
|  |  |  | *Lcu.2RBY.5g007150* | Chr5 | 12174012 | 12176783 | CYCLIN-DEPENDENT KINASE INHIBITOR 6 |
|  |  |  | *Lcu.2RBY.5g007120* | Chr5 | 12189204 | 12195628 | PROTEASE FAMILY M24 METHIONYL AMINOPEPTIDASE%2C AMINOPEPTIDASE P // METHIONINE AMINOPEPTIDASE 1D%2C MITOCHONDRIAL |
|  |  |  | *Lcu.2RBY.5g007130* | Chr5 | 12200172 | 12236911 | Ca2+-independent phospholipase A2 |
|  |  |  | *Lcu.2RBY.5g007140* | Chr5 | 12239717 | 12240963 | AMP-ACTIVATED PROTEIN KINASE%2C GAMMA REGULATORY SUBUNIT // CBS DOMAIN-CONTAINING PROTEIN CBSCBSPB1-RELATED |
|  |  |  | *Lcu.2RBY.5g007150* | Chr5 | 12269434 | 12271712 | AMP-ACTIVATED PROTEIN KINASE%2C GAMMA REGULATORY SUBUNIT // CBS DOMAIN-CONTAINING PROTEIN CBSCBSPB1-RELATED |
| Chr5_342836807 | DF | 10 | *Lcu.2RBY.5g047160* | Chr5 | 342453503 | 342455195 | UDP-GLYCOSYLTRANSFERASE |
|  |  |  | *Lcu.2RBY.5g047180* | Chr5 | 342511112 | 342512990 | UDP-GLYCOSYLTRANSFERASE |
|  |  |  | *Lcu.2RBY.5g047190* | Chr5 | 342513025 | 342519821 | RNA-binding motif protein |
|  |  |  | *Lcu.2RBY.5g047210* | Chr5 | 342665135 | 342666034 | NADH dehydrogenase / Type I dehydrogenase |
|  |  |  | *Lcu.2RBY.5g047220* | Chr5 | 342666111 | 342666526 | protein_coding_gene |
|  |  |  | *Lcu.2RBY.5g047240* | Chr5 | 342666604 | 342669896 | NADH dehydrogenase (ubiquinone) Fe-S protein 2 (NDUFS2 |
|  |  |  | *Lcu.2RBY.5g047250* | Chr5 | 342891879 | 342894245 | DEHYDRODOLICHYL DIPHOSPHATE SYNTHASE // DEHYDRODOLICHYL DIPHOSPHATE SYNTHASE 1 |
|  |  |  | *Lcu.2RBY.5g047270* | Chr5 | 343162560 | 343168605 | DNA damage-binding protein 2 (DDB2) |
|  |  |  | *Lcu.2RBY.5g047280* | Chr5 | 343175177 | 343194016 | Iron-chelate-transporting ATPase |
|  |  |  | *Lcu.2RBY.5g047290* | Chr5 | 343194183 | 343198445 | Ca2+:H+ antiporter |
| Chr5_138162617 | HSW | 6 | *Lcu.2RBY.5g026890* | Chr5 | 137953419 | 137979707 | EamA-like transporter family protein |
|  |  |  | *Lcu.2RBY.5g026900* | Chr5 | 138043933 | 138045085 | Transcription factor IIIA |
|  |  |  | *Lcu.2RBY.5g026910* | Chr5 | 138059421 | 138059663 | (R)-mandelonitrile lyase |
|  |  |  | *Lcu.2RBY.5g026920* | Chr5 | 138059836 | 138060525 | (R)-mandelonitrile lyase |
|  |  |  | *Lcu.2RBY.5g026930* | Chr5 | 138089123 | 138090652 | Cytochrome P450 89A2-related protein |
|  |  |  | *Lcu.2RBY.5g026940* | Chr5 | 138226349 | 138228568 | Gnk2-homologous / homeodomain-like protein |
| Chr5_413492767 | HSW | 21 | *Lcu.2RBY.5g056400* | Chr5 | 413034705 | 413040662 | Uncharacterized protein |
|  |  |  | *Lcu.2RBY.5g056410* | Chr5 | 413052365 | 413056372 | Signal peptidase complex subunit 2 (SPCS2) |
|  |  |  | *Lcu.2RBY.5g056420* | Chr5 | 413071379 | 413071579 | Uncharacterized protein |
|  |  |  | *Lcu.2RBY.5g056430* | Chr5 | 413076089 | 413081689 | Peroxisomal adenine nucleotide transporter (SLC25A17) |
|  |  |  | *Lcu.2RBY.5g056440* | Chr5 | 413098004 | 413100243 | Uncharacterized protein |
|  |  |  | *Lcu.2RBY.5g056450* | Chr5 | 413121021 | 413122321 | NAD(P)H oxidase / thyroid oxidase-like protein |
|  |  |  | *Lcu.2RBY.5g056460* | Chr5 | 413249114 | 413251016 | Shikimate O-hydroxycinnamoyltransferase |
|  |  |  | *Lcu.2RBY.5g056470* | Chr5 | 413321491 | 413322171 | Armadillo/beta-catenin-like repeat protein |
|  |  |  | *Lcu.2RBY.5g056480* | Chr5 | 413394119 | 413395119 | Fasciclin domain-containing protein |
|  |  |  | *Lcu.2RBY.5g056490* | Chr5 | 413394900 | 413399623 | 26S proteasome regulatory subunit N8 |
|  |  |  | *Lcu.2RBY.5g056500* | Chr5 | 413416899 | 413417928 | Homeobox domain-containing protein |
|  |  |  | *Lcu.2RBY.5g056510* | Chr5 | 413445886 | 413455878 | Translation initiation factor 3 subunit F |
|  |  |  | *Lcu.2RBY.5g056520* | Chr5 | 413495387 | 413498164 | Aquaporin PIP1-4-related protein |
|  |  |  | *Lcu.2RBY.5g056530* | Chr5 | 413629418 | 413633533 | Glycosyltransferase family 64 protein (EPC1) |
|  |  |  | *Lcu.2RBY.5g056540* | Chr5 | 413633302 | 413635413 | Uncharacterized protein |
|  |  |  | *Lcu.2RBY.5g056550* | Chr5 | 413640068 | 413640799 | Pentatricopeptide repeat (PPR) protein |
|  |  |  | *Lcu.2RBY.5g056580* | Chr5 | 413706752 | 413708816 | Auxin response factor 17 |
|  |  |  | *Lcu.2RBY.5g056590* | Chr5 | 413734241 | 413738407 | Hs1pro-1 domain-containing protein |
|  |  |  | *Lcu.2RBY.5g056600* | Chr5 | 413757542 | 413763879 | Inositol transporter 4-related protein |
|  |  |  | *Lcu.2RBY.5g056610* | Chr5 | 413763953 | 413764624 | Serine/threonine protein kinase-related |
|  |  |  | *Lcu.2RBY.5g056620* | Chr5 | 413781832 | 413785097 | Large subunit ribosomal protein LP0 |
| Chr5_471647151 | HSW | 36 | *Lcu.2RBY.5g072880* | Chr5 | 471196180 | 471198418 | E3 ubiquitin ligase |
|  |  |  | *Lcu.2RBY.5g072890* | Chr5 | 471204871 | 471208386 | Uncharacterized protein |
|  |  |  | *Lcu.2RBY.5g072900* | Chr5 | 471214003 | 471218677 | B3 DNA-binding domain protein |
|  |  |  | *Lcu.2RBY.5g072920* | Chr5 | 471225531 | 471231333 | B3 domain-containing protein REM16 |
|  |  |  | *Lcu.2RBY.5g072950* | Chr5 | 471246866 | 471261877 | B3 domain-containing protein REM16 |
|  |  |  | *Lcu.2RBY.5g072960* | Chr5 | 471322451 | 471327794 | Uncharacterized protein |
|  |  |  | *Lcu.2RBY.5g072970* | Chr5 | 471328330 | 471331678 | B3 domain-containing protein REM16 |
|  |  |  | *Lcu.2RBY.5g072990* | Chr5 | 471372070 | 471375633 | Transcription repressor KAN1-related |
|  |  |  | *Lcu.2RBY.5g073000* | Chr5 | 471403233 | 471408837 | Serine/threonine protein kinase |
|  |  |  | *Lcu.2RBY.5g073010* | Chr5 | 471410636 | 471413125 | F-box and WD40 domain protein |
|  |  |  | *Lcu.2RBY.5g073020* | Chr5 | 471499308 | 471504994 | Cysteine-rich receptor-like protein kinase 28 |
|  |  |  | *Lcu.2RBY.5g073030* | Chr5 | 471522967 | 471526090 | Cysteine-rich receptor-like protein kinase 28 |
|  |  |  | *Lcu.2RBY.5g073040* | Chr5 | 471537864 | 471539499 | Cysteine-rich repeat secretory protein |
|  |  |  | *Lcu.2RBY.5g073050* | Chr5 | 471571479 | 471575987 | Cysteine-rich receptor-like protein kinase 28 |
|  |  |  | *Lcu.2RBY.5g073060* | Chr5 | 471614685 | 471616615 | Hydroperoxide dehydratase / isomerase |
|  |  |  | *Lcu.2RBY.5g073070* | Chr5 | 471652349 | 471669141 | Vps52 / exocyst component-related protein |
|  |  |  | *Lcu.2RBY.5g073080* | Chr5 | 471685646 | 471689803 | Alkaline ceramidase-related protein |
|  |  |  | *Lcu.2RBY.5g073090* | Chr5 | 471692560 | 471694806 | Ribosomal protein S9, chloroplastic |
|  |  |  | *Lcu.2RBY.5g073100* | Chr5 | 471708041 | 471711381 | MATE efflux family protein |
|  |  |  | *Lcu.2RBY.5g073110* | Chr5 | 471712213 | 471716825 | F-box domain protein |
|  |  |  | *Lcu.2RBY.5g073120* | Chr5 | 471717759 | 471718151 | GRF zinc finger protein |
|  |  |  | *Lcu.2RBY.5g073140* | Chr5 | 471779093 | 471787037 | Inositol-1,4,5-trisphosphate 5-phosphatase |
|  |  |  | *Lcu.2RBY.5g073160* | Chr5 | 471892035 | 471895463 | Histone deacetylase complex subunit SAP30L |
|  |  |  | *Lcu.2RBY.5g073170* | Chr5 | 471917815 | 471922053 | mRNA methyltransferase |
|  |  |  | *Lcu.2RBY.5g073180* | Chr5 | 471945105 | 471948590 | Brassinazole-resistant 1-related protein |
|  |  |  | *Lcu.2RBY.5g073190* | Chr5 | 471971117 | 471972730 | Peroxisomal biogenesis protein (peroxin) |
|  |  |  | *Lcu.2RBY.5g073200* | Chr5 | 471979857 | 471985474 | Lysophosphatidylcholine acyltransferase |
|  |  |  | *Lcu.2RBY.5g073210* | Chr5 | 471992263 | 471995432 | Putative thiol peptidase family protein |
|  |  |  | *Lcu.2RBY.5g073220* | Chr5 | 472035384 | 472038439 | Reticulon / arabinosyltransferase |
|  |  |  | *Lcu.2RBY.5g073230* | Chr5 | 472042667 | 472048031 | Protein phosphatase 2C (PTC7 homolog) |
|  |  |  | *Lcu.2RBY.5g073240* | Chr5 | 472063093 | 472065883 | Uncharacterized protein |
|  |  |  | *Lcu.2RBY.5g073250* | Chr5 | 472069658 | 472072152 | Chloroplast outer envelope pore protein |
|  |  |  | *Lcu.2RBY.5g073260* | Chr5 | 472079599 | 472083277 | DUF620 domain-containing protein |
|  |  |  | *Lcu.2RBY.5g073270* | Chr5 | 472114540 | 472123864 | GRB10 INTERACTING GYF PROTEIN/GYF domain-containing protein |
|  |  |  | *Lcu.2RBY.5g073280* | Chr5 | 472124619 | 472127916 | Heat shock protein 70 |
|  |  |  | *Lcu.2RBY.5g073300* | Chr5 | 472129992 | 472131518 | Protein phosphatase 2A regulatory subunit B |
| Chr6_200603138 | DF | 4 | *Lcu.2RBY.6g025820* | Chr6 | 200598619 | 200599010 | Uncharacterized protein |
|  |  |  | *Lcu.2RBY.6g025870* | Chr6 | 200674490 | 200675835 | Ribosomal protein L10e/L16 (Longin-like domain) |
|  |  |  | *Lcu.2RBY.6g025900* | Chr6 | 200896127 | 200900959 | Dolichol-phosphate mannosyltransferase subunit 1 |
|  |  |  | *Lcu.2RBY.6g025940* | Chr6 | 201042524 | 201045821 | Mitochondrial phosphate transporter (SLC25 family) |
| Chr6_294199001 | DM | 5 | *Lcu.2RBY.6g041210* | Chr6 | 293943012 | 293946814 | Uncharacterized protein |
|  |  |  | *Lcu.2RBY.6g041220* | Chr6 | 293949065 | 293953953 | Uncharacterized protein |
|  |  |  | *Lcu.2RBY.6g041250* | Chr6 | 294241467 | 294243783 | Probable lipid transfer protein (LTP) |
|  |  |  | *Lcu.2RBY.6g041270* | Chr6 | 294336769 | 294337594 | Uncharacterized protein |
|  |  |  | *Lcu.2RBY.6g041280* | Chr6 | 294590804 | 294606415 | Uncharacterized protein |
| Chr6_4338291 | DM | 35 | *Lcu.2RBY.6g000990* | Chr6 | 3830350 | 3843010 | Casein kinase-like protein |
|  |  |  | *Lcu.2RBY.6g001000* | Chr6 | 3843628 | 3845939 | Uncharacterized protein |
|  |  |  | *Lcu.2RBY.6g001020* | Chr6 | 3892943 | 3895648 | Uncharacterized protein |
|  |  |  | *Lcu.2RBY.6g001040* | Chr6 | 3921387 | 3921609 | Uncharacterized protein |
|  |  |  | *Lcu.2RBY.6g001030* | Chr6 | 3921700 | 3921968 | Uncharacterized protein |
|  |  |  | *Lcu.2RBY.6g001050* | Chr6 | 3936730 | 3937240 | Uncharacterized protein |
|  |  |  | *Lcu.2RBY.6g001060* | Chr6 | 3946022 | 3949065 | Legumain (LGMN) |
|  |  |  | *Lcu.2RBY.6g001070* | Chr6 | 4047816 | 4050723 | High mobility group B protein 10-related |
|  |  |  | *Lcu.2RBY.6g001080* | Chr6 | 4150324 | 4156093 | Transcription initiation factor TFIID subunit 10 (TAF10) |
|  |  |  | *Lcu.2RBY.6g001090* | Chr6 | 4163088 | 4188505 | Callose synthase |
|  |  |  | *Lcu.2RBY.6g001100* | Chr6 | 4209425 | 4215871 | WDSAM1 protein |
|  |  |  | *Lcu.2RBY.6g001110* | Chr6 | 4214192 | 4216931 | Oligopeptide transporter-related protein |
|  |  |  | *Lcu.2RBY.6g001120* | Chr6 | 4219690 | 4224517 | Myo-inositol transporter (MFS family) |
|  |  |  | *Lcu.2RBY.6g001130* | Chr6 | 4237075 | 4239241 | Anaphase-promoting complex subunit 11 |
|  |  |  | *Lcu.2RBY.6g001140* | Chr6 | 4270366 | 4272238 | Peptidase of plants and bacteria |
|  |  |  | *Lcu.2RBY.6g001150* | Chr6 | 4273804 | 4274204 | MYB transcription factor |
|  |  |  | *Lcu.2RBY.6g001160* | Chr6 | 4277110 | 4277157 | Uncharacterized protein |
|  |  |  | *Lcu.2RBY.6g001170* | Chr6 | 4283971 | 4288865 | Disease resistance / LRR family protein |
|  |  |  | *Lcu.2RBY.6g001180* | Chr6 | 4289488 | 4292849 | Nitric-oxide synthase-like protein |
|  |  |  | *Lcu.2RBY.6g001190* | Chr6 | 4304930 | 4310443 | Cytochrome-b5 reductase |
|  |  |  | *Lcu.2RBY.6g001210* | Chr6 | 4368931 | 4371061 | Uncharacterized protein |
|  |  |  | *Lcu.2RBY.6g001220* | Chr6 | 4375345 | 4378180 | Ras-related protein Rab-11A |
|  |  |  | *Lcu.2RBY.6g001230* | Chr6 | 4465622 | 4469770 | CDP-diacylglycerol–glycerol-3-phosphate phosphatidyltransferase |
|  |  |  | *Lcu.2RBY.6g001240* | Chr6 | 4470302 | 4470716 | Transcription initiation factor IIA subunit 2 |
|  |  |  | *Lcu.2RBY.6g001250* | Chr6 | 4545719 | 4547865 | Pirin-related protein |
|  |  |  | *Lcu.2RBY.6g001260* | Chr6 | 4552156 | 4555002 | Small GTP-binding protein |
|  |  |  | *Lcu.2RBY.6g001270* | Chr6 | 4566749 | 4576135 | Collagenase-like metalloprotease |
|  |  |  | *Lcu.2RBY.6g001280* | Chr6 | 4576194 | 4577836 | Pollen Ole e 1 allergen / extensin family protein |
|  |  |  | *Lcu.2RBY.6g001290* | Chr6 | 4580330 | 4581598 | Pollen Ole e 1 allergen / extensin family protein |
|  |  |  | *Lcu.2RBY.6g001300* | Chr6 | 4590414 | 4590842 | Cation/H⁺ antiporter-related protein |
|  |  |  | *Lcu.2RBY.6g001310* | Chr6 | 4610252 | 4614484 | Low PSII accumulation 3 protein |
|  |  |  | *Lcu.2RBY.6g001320* | Chr6 | 4634514 | 4637817 | XS/XH domain-containing protein |
|  |  |  | *Lcu.2RBY.6g001330* | Chr6 | 4693126 | 4694221 | SAUR family protein |
|  |  |  | *Lcu.2RBY.6g001340* | Chr6 | 4726827 | 4728519 | Bidirectional sugar transporter SWEET15 |
|  |  |  | *Lcu.2RBY.6g001350* | Chr6 | 4814628 | 4816099 | Bidirectional sugar transporter SWEET15 |
| Chr6_109937017 | HSW | 7 | *Lcu.2RBY.6g014380* | Chr6 | 109808052 | 109808273 | Uncharacterized protein |
|  |  |  | *Lcu.2RBY.6g014390* | Chr6 | 109873790 | 109888188 | Importin beta family protein |
|  |  |  | *Lcu.2RBY.6g014400* | Chr6 | 109918370 | 109918876 | Uncharacterized protein |
|  |  |  | *Lcu.2RBY.6g014410* | Chr6 | 109932760 | 109935959 | Tryptophan/tyrosine permease / ORC subunit 6–related protein |
|  |  |  | *Lcu.2RBY.6g014420* | Chr6 | 110076568 | 110077189 | Uncharacterized protein |
|  |  |  | *Lcu.2RBY.6g014450* | Chr6 | 110194820 | 110197998 | Amino acid transporter–related protein |
|  |  |  | *Lcu.2RBY.6g014480* | Chr6 | 110356801 | 110363193 | 20S proteasome subunit beta 2 (PSMB7) |
| Chr6_110350822 | HSW | 10 | *Lcu.2RBY.6g014390* | Chr6 | 109873790 | 109888188 | Importin beta family protein |
|  |  |  | *Lcu.2RBY.6g014400* | Chr6 | 109918370 | 109918876 | Uncharacterized protein |
|  |  |  | *Lcu.2RBY.6g014410* | Chr6 | 109932760 | 109935959 | Tryptophan/tyrosine permease / ORC subunit 6–related protein |
|  |  |  | *Lcu.2RBY.6g014420* | Chr6 | 110076568 | 110077189 | Uncharacterized protein |
|  |  |  | *Lcu.2RBY.6g014450* | Chr6 | 110194820 | 110197998 | Amino acid transporter–related protein |
|  |  |  | *Lcu.2RBY.6g014480* | Chr6 | 110356801 | 110363193 | 20S proteasome subunit beta 2 (PSMB7) |
|  |  |  | *Lcu.2RBY.6g014490* | Chr6 | 110465233 | 110482865 | Coatomer subunit delta |
|  |  |  | *Lcu.2RBY.6g014500* | Chr6 | 110540844 | 110547063 | U4/U6 snRNP protein PRP31 (PRPF31) |
|  |  |  | *Lcu.2RBY.6g014510* | Chr6 | 110623113 | 110627431 | Glucose-6-phosphate 1-dehydrogenase (G6PD) |
|  |  |  | *Lcu.2RBY.6g014520* | Chr6 | 110772881 | 110786276 | GPI inositol-deacylase |
| Chr6_117959608 | HSW | 6 | *Lcu.2RBY.6g015520* | Chr6 | 117715208 | 117715659 | GAG–POL-related retrotransposon protein |
|  |  |  | *Lcu.2RBY.6g015530* | Chr6 | 117716088 | 117716172 | Uncharacterized protein |
|  |  |  | *Lcu.2RBY.6g015550* | Chr6 | 117784586 | 117785023 | GAG/POL/ENV polyprotein-related |
|  |  |  | *Lcu.2RBY.6g015560* | Chr6 | 117801017 | 117801433 | RNA-directed DNA polymerase / RNase H (retrotransposon-related) |
|  |  |  | *Lcu.2RBY.6g015580* | Chr6 | 118182238 | 118216461 | Peptide-transporting ATPase |
|  |  |  | *Lcu.2RBY.6g015610* | Chr6 | 118333653 | 118339058 | RNA-directed DNA methylation 4-related protein |
| Chr6_128041250 | HSW | 5 | *Lcu.2RBY.6g016500* | Chr6 | 127669913 | 127670534 | Uncharacterized protein |
|  |  |  | *Lcu.2RBY.6g016510* | Chr6 | 127698417 | 127700343 | E3 ubiquitin ligase–interacting protein |
|  |  |  | *Lcu.2RBY.6g016520* | Chr6 | 127791290 | 127793453 | Scarecrow-like protein 22–related |
|  |  |  | *Lcu.2RBY.6g016540* | Chr6 | 127800820 | 127810852 | Leucine-rich repeat, cysteine-containing protein |
|  |  |  | *Lcu.2RBY.6g016550* | Chr6 | 127816932 | 127817986 | Ulp1 protease family protein (C48 peptidase) |
| Chr6_224444128 | HSW | 11 | *Lcu.2RBY.6g029490* | Chr6 | 224671013 | 224673519 | GDP-mannose transporter (GONST1/VRG4) |
|  |  |  | *Lcu.2RBY.6g029510* | Chr6 | 224719907 | 224721679 | Trihelix transcription factor GT-3A–related |
|  |  |  | *Lcu.2RBY.6g029520* | Chr6 | 224741018 | 224743056 | Uncharacterized protein |
|  |  |  | *Lcu.2RBY.6g029540* | Chr6 | 224830486 | 224833701 | Fatty acid desaturase (Δ12-desaturase) |
|  |  |  | *Lcu.2RBY.6g029550* | Chr6 | 224868801 | 224872410 | Plant transposase (En/Spm family) |
|  |  |  | *Lcu.2RBY.6g029560* | Chr6 | 224873856 | 224874029 | Thymidine kinase |
|  |  |  | *Lcu.2RBY.6g029570* | Chr6 | 224875369 | 224886653 | Copine / BONZAI 3 protein |
|  |  |  | *Lcu.2RBY.6g029580* | Chr6 | 224894525 | 224903060 | Syntaxin-binding protein 5 (STXBP5) |
|  |  |  | *Lcu.2RBY.6g029590* | Chr6 | 224926775 | 224931733 | Acyl-CoA oxidase 4, peroxisomal |
|  |  |  | *Lcu.2RBY.6g029600* | Chr6 | 224931622 | 224932980 | CREB-related transcription factor |
|  |  |  | *Lcu.2RBY.6g029610* | Chr6 | 224932338 | 224932676 | Lactaldehyde dehydrogenase |
| Chr6_253032265 | HSW | 5 | *Lcu.2RBY.6g034130* | Chr6 | 252693661 | 252696961 | Vestitone reductase |
|  |  |  | *Lcu.2RBY.6g034140* | Chr6 | 252852519 | 252853276 | Uncharacterized protein |
|  |  |  | *Lcu.2RBY.6g034160* | Chr6 | 252976976 | 252980589 | Vestitone reductase |
|  |  |  | *Lcu.2RBY.6g034170* | Chr6 | 253025289 | 253027932 | Vestitone reductase |
|  |  |  | *Lcu.2RBY.6g034180* | Chr6 | 253227873 | 253247364 | Myosin-10–related protein |
| Chr6_344313403 | PPP and YPP | 13 | *Lcu.2RBY.6g050480* | Chr6 | 344010906 | 344011551 | Uncharacterized protein |
|  |  |  | *Lcu.2RBY.6g050510* | Chr6 | 344218788 | 344221616 | RNA polymerase subunit RPB1 (N-terminal domain) |
|  |  |  | *Lcu.2RBY.6g050520* | Chr6 | 344238864 | 344244001 | Serine/threonine protein kinase NEK5 |
|  |  |  | *Lcu.2RBY.6g050530* | Chr6 | 344245088 | 344248293 | DnaJ homolog subfamily C member 9 (DNAJC9) |
|  |  |  | *Lcu.2RBY.6g050540* | Chr6 | 344253968 | 344256366 | Lysophosphatidic acid acyltransferase (AGPAT) |
|  |  |  | *Lcu.2RBY.6g050550* | Chr6 | 344316573 | 344318983 | Uncharacterized protein |
|  |  |  | *Lcu.2RBY.6g050560* | Chr6 | 344319665 | 344321660 | Phosphoglycerate mutase |
|  |  |  | *Lcu.2RBY.6g050580* | Chr6 | 344445944 | 344448190 | Uncharacterized protein |
|  |  |  | *Lcu.2RBY.6g050590* | Chr6 | 344458117 | 344461771 | RNA polymerase subunit RPB6 |
|  |  |  | *Lcu.2RBY.6g050600* | Chr6 | 344473137 | 344477911 | Nucleolar protein GAR2-like |
|  |  |  | *Lcu.2RBY.6g050610* | Chr6 | 344533722 | 344537721 | Exopolygalacturonosidase |
|  |  |  | *Lcu.2RBY.6g050620* | Chr6 | 344538798 | 344546658 | tRNA splicing endonuclease |
|  |  |  | *Lcu.2RBY.6g050640* | Chr6 | 344705218 | 344711549 | bHLH transcription factor BHLH4 |
| Chr6_262648494 | YPP | 11 | *Lcu.2RBY.6g035280* | Chr6 | 262165245 | 262166446 | Plastocyanin-like copper-binding protein |
|  |  |  | *Lcu.2RBY.6g035290* | Chr6 | 262332654 | 262341836 | C2 calcium/lipid-binding endonuclease/exonuclease/phosphatase-related protein |
|  |  |  | *Lcu.2RBY.6g035300* | Chr6 | 262375915 | 262376305 | RNA-directed DNA polymerase / reverse transcriptase |
|  |  |  | *Lcu.2RBY.6g035310* | Chr6 | 262409589 | 262415002 | Glyoxylate reductase (NADP⁺-dependent) |
|  |  |  | *Lcu.2RBY.6g035320* | Chr6 | 262414214 | 262420461 | Polygalacturonate 4-α-galacturonosyltransferase-related protein |
|  |  |  | *Lcu.2RBY.6g035340* | Chr6 | 262583597 | 262586562 | AGAMOUS-like MADS-box transcription factor |
|  |  |  | *Lcu.2RBY.6g035350* | Chr6 | 262769643 | 262778354 | MADS-box transcription factor SOC1-like |
|  |  |  | *Lcu.2RBY.6g035360* | Chr6 | 262850404 | 262850781 | Uncharacterized protein |
|  |  |  | *Lcu.2RBY.6g035370* | Chr6 | 262852313 | 262853924 | Uncharacterized protein |
|  |  |  | *Lcu.2RBY.6g035380* | Chr6 | 262961332 | 262964000 | CRS2-associated factor 1, mitochondrial |
|  |  |  | *Lcu.2RBY.6g035390* | Chr6 | 262964364 | 262967987 | Subtilase family protease |
| Chr7_9794560 | HSW | 24 | *Lcu.2RBY.7g005280* | Chr7 | 9334121 | 9338728 | Regulator of chromosome condensation (RCC2) |
|  |  |  | *Lcu.2RBY.7g005290* | Chr7 | 9354664 | 9362169 | Apoptosis inhibitor 5-related protein |
|  |  |  | *Lcu.2RBY.7g005300* | Chr7 | 9377515 | 9379737 | MDC16-related protein |
|  |  |  | *Lcu.2RBY.7g005310* | Chr7 | 9496557 | 9499154 | Charged multivesicular body protein (CHMP2A) |
|  |  |  | *Lcu.2RBY.7g005320* | Chr7 | 9499174 | 9499641 | RING-H2 finger protein (ATL33-related) |
|  |  |  | *Lcu.2RBY.7g005330* | Chr7 | 9545147 | 9554550 | MYB family transcription factor (TRFL6-related) |
|  |  |  | *Lcu.2RBY.7g005350* | Chr7 | 9627572 | 9630813 | Reticulocalbin |
|  |  |  | *Lcu.2RBY.7g005360* | Chr7 | 9656746 | 9662916 | Reticulocalbin-related protein |
|  |  |  | *Lcu.2RBY.7g005370* | Chr7 | 9657895 | 9658362 | Zinc-binding reverse transcriptase-like protein |
|  |  |  | *Lcu.2RBY.7g005380* | Chr7 | 9709400 | 9712179 | Reticulocalbin-related protein |
|  |  |  | *Lcu.2RBY.7g005400* | Chr7 | 9721949 | 9722941 | Leucine-rich repeat-containing protein |
|  |  |  | *Lcu.2RBY.7g005410* | Chr7 | 9724315 | 9726835 | Uncharacterized protein |
|  |  |  | *Lcu.2RBY.7g005420* | Chr7 | 9727931 | 9730580 | Reticulocalbin-related protein |
|  |  |  | *Lcu.2RBY.7g005430* | Chr7 | 9735035 | 9741145 | Leucine-rich repeat-containing protein |
|  |  |  | *Lcu.2RBY.7g005470* | Chr7 | 9948991 | 9949335 | EARLY FLOWERING 4 |
|  |  |  | *Lcu.2RBY.7g005480* | Chr7 | 9950613 | 9957777 | Uncharacterized protein |
|  |  |  | *Lcu.2RBY.7g005490* | Chr7 | 9995690 | 10019002 | TIR–NB-ARC–LRR disease resistance protein |
|  |  |  | *Lcu.2RBY.7g005500* | Chr7 | 10035261 | 10044097 | Helicase SEN1 |
|  |  |  | *Lcu.2RBY.7g005510* | Chr7 | 10120485 | 10123968 | Metallophosphoesterase-related protein |
|  |  |  | *Lcu.2RBY.7g005520* | Chr7 | 10126219 | 10130796 | Transcription initiation factor TAF12 |
|  |  |  | *Lcu.2RBY.7g005530* | Chr7 | 10193338 | 10206667 | Transcription initiation factor TAF12 |
|  |  |  | *Lcu.2RBY.7g005540* | Chr7 | 10207264 | 10208438 | MYB-like DNA-binding protein |
|  |  |  | *Lcu.2RBY.7g005550* | Chr7 | 10220581 | 10223963 | Autophagy-related protein ATG12 |
|  |  |  | *Lcu.2RBY.7g005560* | Chr7 | 10226493 | 10229767 | AP2-like ethylene-responsive transcription factor |
| Chr7_42734754 | DM | 1 | *Lcu.2RBY.7g011600* | Chr7 | 42261383 | 42262063 | RNA helicase (EC 3.6.4.13) |
| Chr7_295399575 | DF | 11 | *Lcu.2RBY.7g039780* | Chr7 | 295223003 | 295225235 | NAC domain-containing protein |
|  |  |  | *Lcu.2RBY.7g039790* | Chr7 | 295258679 | 295259101 | Lysosomal amino acid transporter |
|  |  |  | *Lcu.2RBY.7g039800* | Chr7 | 295259636 | 295260552 | DUF309 domain-containing protein |
|  |  |  | *Lcu.2RBY.7g039810* | Chr7 | 295413230 | 295414268 | Uncharacterized protein |
|  |  |  | *Lcu.2RBY.7g039820* | Chr7 | 295526730 | 295528295 | GRAS domain transcription factor |
|  |  |  | *Lcu.2RBY.7g039830* | Chr7 | 295528187 | 295539911 | Serine/threonine protein kinase |
|  |  |  | *Lcu.2RBY.7g039840* | Chr7 | 295803140 | 295804769 | Amino acid transporter |
|  |  |  | *Lcu.2RBY.7g039850* | Chr7 | 295827208 | 295827843 | Uncharacterized protein |
|  |  |  | *Lcu.2RBY.7g039860* | Chr7 | 295828754 | 295829146 | Inositol 5-phosphatase |
|  |  |  | *Lcu.2RBY.7g039870* | Chr7 | 295829386 | 295829892 | Uncharacterized protein |
|  |  |  | *Lcu.2RBY.7g039880* | Chr7 | 295881562 | 295883567 | Receptor-like protein kinase (TMK1-related) |
| Chr7_297717438 | PH | 8 | *Lcu.2RBY.7g039990* | Chr7 | 297340004 | 297340396 | GRF zinc finger protein |
|  |  |  | *Lcu.2RBY.7g040000* | Chr7 | 297419806 | 297421419 | Uncharacterized protein |
|  |  |  | *Lcu.2RBY.7g040020* | Chr7 | 297477153 | 297480983 | Aspartyl protease family protein |
|  |  |  | *Lcu.2RBY.7g040030* | Chr7 | 297944036 | 297947291 | Uncharacterized protein |
|  |  |  | *Lcu.2RBY.7g040040* | Chr7 | 297969361 | 297969639 | Rho/Rac/CDC GTPase-activating protein |
|  |  |  | *Lcu.2RBY.7g040050* | Chr7 | 297993184 | 297994208 | RNA recognition motif–containing protein |
|  |  |  | *Lcu.2RBY.7g040060* | Chr7 | 298081436 | 298085203 | Aspartyl protease family protein |
|  |  |  | *Lcu.2RBY.7g040070* | Chr7 | 298086022 | 298087905 | Peroxidase 64 |
| Chr7_337510839 | HSW | 3 | *Lcu.2RBY.7g044650* | Chr7 | 337377296 | 337378375 | Protein FAM86A |
|  |  |  | *Lcu.2RBY.7g044660* | Chr7 | 337460155 | 337461310 | Protein FAM86A |
|  |  |  | *Lcu.2RBY.7g044680* | Chr7 | 337561643 | 337563289 | Tubby-related / Tubby-like F-box protein 11 |
| Chr7_338463151 | DM | 7 | *Lcu.2RBY.7g044700* | Chr7 | 338080452 | 338088033 | VAM6 / VPS39-like protein |
|  |  |  | *Lcu.2RBY.7g044710* | Chr7 | 338095648 | 338096097 | Uncharacterized protein |
|  |  |  | *Lcu.2RBY.7g044730* | Chr7 | 338141645 | 338142793 | Protein EFR3 homolog |
|  |  |  | *Lcu.2RBY.7g044740* | Chr7 | 338144903 | 338147636 | MATE efflux family protein (ALF5-related) |
|  |  |  | *Lcu.2RBY.7g044760* | Chr7 | 338363632 | 338367692 | Retinaldehyde-binding protein–related |
|  |  |  | *Lcu.2RBY.7g044770* | Chr7 | 338380473 | 338381765 | Long-chain fatty-acid–ACP ligase |
|  |  |  | *Lcu.2RBY.7g044780* | Chr7 | 338412981 | 338413837 | Alternative splicing factor (SR protein) |
| Chr7_407008237 | DM | 5 | *Lcu.2RBY.7g051600* | Chr7 | 406615599 | 406616909 | Glycosyltransferase 6-related protein |
|  |  |  | *Lcu.2RBY.7g051640* | Chr7 | 406775486 | 406777360 | OXA1 family membrane protein |
|  |  |  | *Lcu.2RBY.7g051680* | Chr7 | 407029967 | 407030712 | Uncharacterized protein |
|  |  |  | *Lcu.2RBY.7g051700* | Chr7 | 407062303 | 407065079 | Ribosomal protein S11 / glyoxalase-like protein |
|  |  |  | *Lcu.2RBY.7g051710* | Chr7 | 407139062 | 407139707 | Uncharacterized protein |

****
